# TC2N maintains stem cell-like characteristics to accelerate lung carcinogenesis by blockade of dual specificity protein phosphatase 3

**DOI:** 10.1186/s13578-025-01348-3

**Published:** 2025-01-23

**Authors:** Jing Gu, Yang-fan Lv, Ji-ying Xia, Fu-hai Bai, Ji Gong, Guang-qiang Pan, Bo Liu, Lu Huang, Qiao-nan Guo, Xiang-lin Hao

**Affiliations:** 1 Department of Pathology, Xinqiao Hospital, Third Military Medical University, 183 Xinqiao Street, Shapingba District, Chongqing, 400037 PR China; 2 Institute of Toxicology, College of Preventive Medicine, Third Military Medical University, Chongqing, 400038 PR China; 3 Department of Anesthesiology, Xinqiao Hospital, Third Military Medical University, Chongqing, 400037 PR China

**Keywords:** TC2N, Lung cancer, DUSP3 phosphatase, Urethane, Tumorigenesis, Stemness

## Abstract

**Background:**

Tandem C2 domains, nuclear (TC2N) is a protein that has been characterized to contain C2A domain, C2B domain, and a short C-terminus with a WHXL motif. In previous studies, we have uncovered the oncogenic role and mechanisms of TC2N in lung cancer: TC2N achieves this by inhibiting the p53 signaling pathway and activating the NF-kappaB signaling pathway. Beyond that, its precise function in tumorigenesis is not fully understood.

**Methods:**

TC2N-engineered mice model was used to assess the effect of TC2N knockout on normal lung and urethane-induced carcinogenesis. Tumor tissues of 395 lung cancer patients were subjected to tissue microarray and further assessed the associations of TC2N expression with tumor differentiation degree. The protein levels of TC2N and stem cell markers in cell lines and tissue specimens were monitored by WB and immunohistochemistry. In vitro cell assays were performed to assess the effect of TC2N ectopic expression on the stem cell-like characteristics of lung cancer cells. The downstream signaling pathway or target molecule of TC2N was mined using a combination of transcriptomics and proteomics, and the underlying mechanism was explored by WB and co-IP assays.

**Results:**

Herein, TC2N appeared to have a strong effect in promoting lung tumorigenesis caused by urethane, whereas it seemed to lose its function in the normal lung. Meanwhile, we found that the functional differences of TC2N between lung tumor and normal lung were linked to its potential role in cancer cell stemness. Function-wise, TC2N overexpression maintained stem-like properties of lung cancer cell. Mechanism-wise, TC2N upregulated the phosphorylation of EGFR, ERK, STAT3 and FAK1 to activate these signaling pathways by the inhibition of DUSP3 phosphatase via a dual mechanism. Firstly, TC2N competes with EGFR, ERK, STAT3 and FAK1 for binding to DUSP3. This competition prevents these signaling molecules from being dephosphorylated by DUSP3, resulting in their sustained activation. Secondly, TC2N bind to DUSP3 and restrict the enzyme’s ability to dephosphorylate the signaling molecules.

**Conclusions:**

Overall, this study revealed a previously unknown role and mechanism of TC2N in the regulation of tumorigenesis and stemness in lung cancer cells.

**Supplementary Information:**

The online version contains supplementary material available at 10.1186/s13578-025-01348-3.

## Background

Despite advancements in cancer pathophysiology, diagnosis, prognosis, and therapy, cancer remains a leading cause of death worldwide, contributing to a decrease in life expectancy [1, 2]. Tumorigenesis, considered a “developmental disease,” involves a dedifferentiation process where dysregulation of self-renewal and differentiation leads to malignant cell transformation and the formation of hyper-proliferative, aberrantly differentiated cancerous tissue [3]. Tumor-initiating cells (TICs), also known as cancer stem cells (CSCs), are minor subpopulations of cancer cells with unique abilities for self-renewal and initiating tumorigenesis [4, 5]. Targeting CSCs is seen as a promising strategy for achieving precision medicine in cancer treatment [6]. In the context of cancer development and progression, CSCs can differentiate into various cell types, each with different pathological characteristics, differentiation levels, clinical stages, and drug susceptibilities, which can contribute to treatment failures [7]. While the abnormal activation of JAK/STAT, Hedgehog, WNT, Notch, and PI3K/PTEN signaling pathways has been linked to cancer cell stemness [8], further exploration of these mechanisms is needed to identify new molecular targets for precision medicine.

Previously, we identified a novel differentially expressed gene in lung cancer called Tandem C2 domains, nuclear (TC2N), and conducted a systematic study on its expression patterns, biological functions, and molecular mechanisms. Specifically, we confirmed that TC2N acts as a candidate oncogene in lung cancer by promoting cell proliferation and resisting apoptosis through the inhibition of the p53 signaling pathway, while facilitating metastasis via the activation of the NF-κB signaling pathway. Furthermore, we demonstrated that TC2N represses proliferation in breast cancer by strengthening the PI3K/AKT signaling pathway and inhibits metastasis through blocking fatty acid synthesis [9–11]. Subsequently, we and other researchers both domestically and internationally have gradually revealed the oncogenic roles and mechanisms of TC2N in malignancies such as liver cancer, gastric cancer, and glioma, establishing the significant position of this gene in cancer [12–16]. Additionally, C2 domain-containing proteins have been shown to influence cell stemness and differentiation [17–21]. As a member of the C2 domain protein family, the role of TC2N in stem cells had not been previously reported.

In this study, we investigated the impact of TC2N on tumorigenesis and stemness. Knockout experiments in mice demonstrated that the absence of TC2N delayed the onset of lung tumors induced by urethane treatment. Conversely, overexpression of TC2N in lung cancer cells enhanced their stemness properties. Mechanistically, TC2N promoted the phosphorylation and activation of EGFR, ERK, STAT3, and FAK1 pathways by inhibiting DUSP3 phosphatase. Clinically, high TC2N expression was associated with lower differentiation degree (DD) in lung cancer and correlated with a poorer prognosis for patients. These findings shed light on the promoting role of TC2N in tumorigenesis and cancer cell stemness, offering a comprehensive understanding of TC2N in cancer biology.

## Methods

### Cell culture

Two human lung cancer cell lines A549 and H522 were purchased from Cellcook Biotechnology Co., Ltd (Guangzhou, China) and maintained in RIPM-1640 medium containing 10% fetal bovine serum (Gibco). Cells with TC2N stable overexpression or knockdown were reutilized as described in our previous study [9, 10]. All cells were incubated at 37 °C in a humidified atmosphere with 5% CO_2_.

### Clinical specimen collection and tissue microarray (TMA) construction

A TMA contains 272 lung tumor samples (Cohort 1) used in our previous study [9] was retrospectively analyzed in the present study. Besides, another TMA (Cohort 2) contains a total of 395 lung cancer samples that collected from Xinqiao Hospital Affiliated to Third Military Medical University (Chongqing, China) from January, 2017 to December, 2021 was constructed by tissue microarray instrumentation as previously described [22]. Tumor DD information was obtained by retrospecting the patients’ electronic medical records. The Nottingham system was used to determine the tumor DD [23]. The Ethics Committees in Xinqiao Hospital Affiliated to Third Military Medical University (Chongqing, China) approved the collections of clinical specimens and clinicopathological information. Written-informed consent was obtained before the investigation. All procedures were conducted according to the provisions of the Helsinki Declaration in 1975. For tissue microarray construction, all specimens were fixed in 4% paraformaldehyde fix solution and then paraffin-embedded.

### TC2N knock-out mouse model and genotype identification

C57BL/6J (TC2N-knockout) engineered mice were generated through deletion of exon 3–8 of TC2N (ENSMUST00000162735.7) by a commercial supplier (Cyagen Biosciences, Santa Clara, CA, USA) using the CRISPR-Cas9 technique. Mice were maintained in a controlled environment (12-h light/dark cycle, ad libitum access to food and water). The genotype of the mice was determined by RT-PCR (PCR procedure: pre-denaturation at 95 °C for 5 min; 20 cycles of 98 °C for 30s, 65 °C for 30s with decreasing 0.5 °C each cycle, 72 °C for 45s; 20 cycles of 98 °C for 30s, 55 °C for 30s, 72 °C for 45s and extension of 5 min at 72 °C) and the primer sequences were listed as follows: Tc2n-KO-F- TATAGAAGCTCAGGCAGAGGCAG, Tc2n-KO-R- GGATGAGGGTCAGAGACACTAATG, Tc2n-wt-F- CCTGATTTCCAGGTGTGTTAGTG and Tc2n-wt-R- GACTCAACAAGCTGAAGAACTCCC. The PCR products were performed by electrophoresis as previously described [24]. The schematic diagram of TC2N gene knockout mouse construction and the genotype identification result were showed in Supplementary Figure S1. For carcinogenicity assay, TC2N+/+ (*n* = 5), TC2N-/+ (*n* = 5) and TC2N-/- (*n* = 5) mice were matched for age (6 weeks) and injected with urethane (1 g/kg in 100 µl saline) once a week for 10 consecutive weeks [25]. After 6 months of injection, all mice were sacrificed for lung tumor examinations. For evaluating the effect of TC2N deletion on normal lung, mice aged 6 months were euthanized by dislocation and then lung tissues were extracted for further examinations. The histological diagnosis of lung tumors was performed by two experienced pathologists. Mice experiments conformed to the Guide for Care and Use of Laboratory Animals, and were approved by the Ethics Committees in Xinqiao Hospital, Third Military Medical University (AMUWEC2020475).

### Vector construction and cell transfection

The coding sequences (CDS) of TC2N and the shRNA sequence were chemically synthesized and sequenced by VectorBuilder (Guangzhou, China), and then cloned into pLV[Exp]-CMV > T2A: Puro vectors to construct lentivirus. The specific sequences of the CDS region and shRNA are as previously described [9, 11]. The overexpression vector of DUSP3 and three shRNA interference sequences were chemically synthesized and sequenced by Tsingke Biotech, and then individually cloned into the pLVX-IRES-Puro and pLKO.1-puro vectors. The sequences of the three siRNAs are as follows: shRNA1: CCGGTCCACTGCCGGGAAGGTTATACTCGAGTATAACCTTCCCGGCAGTGGATTTTTT; shRNA2: CCGGCGTGAGGCAGAACCGTGAGATCTCGAGATCTCACGGTTCTGCCTCACGTTTTTT; shRNA3: CCGGGGTCCTTCATGCACGTCAACACTCGAGTGTTGACGTGCATGAAGGACCTTTTTT.

Cell Transfection was performed using Lipofectamine 3000 reagent according to the manufacturer’s instructions. Briefly, cells were seeded in a 6-well plate at a density of 3 × 10^^5^ cells per well and allowed to adhere overnight. For each well, a transfection mixture was prepared by diluting 2 µg of overexpression plasmid or shRNA of DUSP3 in 250 µl of Opti-MEM medium, and separately diluting 5 µl of Lipofectamine 3000 in 250 µl of Opti-MEM medium. The diluted DNA and Lipofectamine 3000 solutions were then combined and incubated at room temperature for 15 min to allow complex formation. The transfection mixture was added dropwise to the cells and gently mixed by rocking the plate back and forth. The cells were then incubated at 37 °C in a CO_2_ incubator for at least 24 h to allow for transfection.

### Hematoxylin and eosin (H&E) staining and immunohistochemical (IHC) analysis

Formalin-fixed paraffin-embedded specimens were sectioned at 5 μm for H&E staining or IHC. The sections were dewaxed three times in xylene and sequentially rehydrated in baths of decreasing methanol content (100/95/70/50%), and followed by incubation with 3% H_2_O_2_ in 90% methanol for 30 min to ensure neutralization of endogenous peroxidase. The slides were stained with hematoxylin and eosin for histological analysis. IHC was performed as described previously [26]. Briefly, the protein expression of TC2N, DUSP3, SOX2, OCT4 and NANOG were detected using their primary antibodies: 1:200 diluted rabbit polyclonal antibody (Cat. #: HPA027549; Sigma) for TC2N; 1:500 diluted rabbit polyclonal antibody (Cat. #: bs-6246R; Bioss) for DUSP3; 1:1000 diluted rabbit polyclonal antibody (Cat. #: 11064-1-AP; Proteintech) for SOX2; 1:1000 diluted rabbit polyclonal antibody (Cat. #: 60242-1-Ig; Proteintech) for OCT4; 1:1000 diluted rabbit polyclonal antibody (Cat. #: 14295-1-AP; Proteintech) for NANOG; 1:200 diluted rabbit polyclonal antibody (Cat. #: 10490-1-AP; Proteintech) for CC10; 1:200 diluted rabbit polyclonal antibody (Cat. #: 10774-1-AP; Proteintech) for SFTPC. A combined score of intensity and distribution was used to categorize the immunohistochemical staining for TC2N and DUSP3, as previously described by us and others [9, 27]. The original histological images were uploaded as supplementary materials.

### Transmission electron microscopy

Transmission electron microscope (TEM) processing was performed to detect ultra-structural features of lung cells according to the previous study [28]. Lung tissues of TC2N engineered mice were dissected and promptly fixed in cold glutaraldehyde (5%) for 24 h. After fixing, the tissue was washed by 0.1 ml PBS (pH 7.2) for 20 min, and post-fixed for 1.5 h with osmium tetroxide (1%). The post-fixed samples were then embedded in epoxy resin and semi-thin sections were prepared. Toluidine-blue stained semi-thin slides were examined and assessed. The ultra-thin sections were examined under a transmission electron microscope (HT7700, Hitachi, Japan).

### Sphere-formation assay

For the sphere-forming assay, single-cell suspensions were plated on 6-well ultralow attachment plates at 1000 cells per well in DMEM/F12 (Gibco) supplemented with B27, epidermal growth factor and basic fibroblast growth factor (R&D Systems). After 5 days for A549 and H522 cells of incubation, the spheres (determined as > 20 cells/spheroid) were counted under an inverted microscope at high magnification. The assays were independently repeated at least three times.

### Flow cytometric analysis

For analysis of CD133 expression, cells were incubated with fluorescein isothiocyanate (CoraLite^®^ Plus 488)-conjugated anti-human CD133 antibody (Cat. #: CL488-66666; Proteintech) according to the manufacturer’s protocol. After incubation for 1 h at 4 °C, the labeled cells were washed thrice with cold PBS and further analyzed using a FACSCalibur flow cytometer (BD Biosciences).

For analysis of side population cells, cells were harvested and resuspended in staining buffer at a concentration of 1 × 10^^6^ cells/ml. Hoechst 33,342 dye (Beyotime) was added at a final concentration of 5 µg/ml and the cells were then incubated at 37 °C for 90 min with intermittent mixing. Following the incubation, the cells were washed with ice-cold staining buffer and centrifuged at 300 g for 5 min. The cell pellet was resuspended in ice-cold staining buffer containing propidium iodide (PI) to exclude dead cells. Flow cytometry analysis was performed using a FACSCalibur flow cytometer (BD Biosciences) with UV excitation. Side population cells were identified based on their low Hoechst 33,342 fluorescence intensity using a 450/20 BP filter for Hoechst blue and a 675 LP filter for Hoechst red. The proportion of side population cells was calculated as a percentage of the total viable cell population. All experiments were performed in triplicate to ensure reproducibility of results.

### Soft agar clonogenicity assay

Single-cell suspensions were plated on 6-well plates at 500 cells per well in 1 ml of growth media containing 1.2% low-melt agarose and layered onto a 1 ml bed of growth media containing 0.7% low-melt agarose. Cells were fed daily with 200 µl of growth media. After 14 days of incubation, cell colony was stained with 0.05% crystal violet and counted under an inverted microscope.

### The public databases

The RNA-Seq data of lung cancer were directly downloaded from TCGA (https://www.cancer.gov/). The RNA expression profiles from GSE157011, GSE66863, GSE60644, GSE41271, GSE31210, GSE41271, GSE11969 and GSE68465 datasets were downloaded from GEO database (https://www.ncbi.nlm.nih.gov/geo/). Import all RNA sequencing and expression files into Excel to generate a new matrix file.

### Gene ontology (GO) analysis

For GO analysis, the RNA-Seq data from TCGA and GEO database were used to analyze the correlation between TC2N expression and all other genes in lung cancer patients. The R project was used to screen for co-expressed genes or proteins of TC2N based on the aforementioned public data, using the same R code as previously employed [29]. All co-expressed genes and DPPs of TC2N were used for GO analysis by using Gene Ontology Resource (http://geneontology.org/). Specifically, log in the website and input co-expressed genes or proteins into the textbox then click “launch” to proceed to the next page. Next, in the “Annotation Data Set” section, select “biological process” or “molecular function” or “cellular component” for data type and click “launch analysis” for getting GO results. The co-expressed genes of TC2N are listed in Supplementary Data S1.

### Gene set enrichment analysis (GSEA)

GSEA is a computational approach used to assess whether a predefined set of genes exhibits statistically significant and consistent differences between two biological states. The TCGA and GEO datasets can be organized into a ranked list based on their differential expression related to the phenotype. The GSEA software (v4.3.2) was downloaded from the official GSEA website (https://www.gsea-msigdb.org/gsea/index.jsp). Specifically, the RNA-Seq data was converted into a ‘GCT’ file (gene.GCT) and subsequently uploaded to the ‘Load data’ section for further analysis. In the ‘Run GSEA’ section, parameters were set, including the gene set database: C5.go.bp.v2023.1.Hs.symbols.gmt (curated) or C5.go.mf.v2023.1.Hs.symbols.gmt (curated); the number of permutations: 1,000; and phenotype labels: using TC2N or DUSP3 as the phenotype. A p-value < 0.05 and a false discovery rate (FDR) < 0.25 were considered significant. The statistical parameters for these processes from the TCGA and GEO datasets are detailed in Supplementary Data S2 and Supplementary Data S3.

### Phosphoproteomics analysis

For protein extraction and digestion, A549 cells with TC2N ectopic expression were first lysed using SDT (4%SDS, 100mM Tris-HCl, 1mM DTT, pH7.6) buffer. Then, trypsin was used to perform protein digestion according to filter-aided sample preparation (FASP) procedure described by Matthias Mann. The digest peptides of each sample were desalted on C18 Cartridges (Empore™ SPE Cartridges C18 (standard density), bed I.D. 7 mm, volume 3 ml, Sigma), concentrated by vacuum centrifugation and reconstituted in 40 µl of 0.1% (v/v) formic acid. For filter-aided sample preparation, 200 µg of proteins for each sample were incorporated into 30 µl SDT buffer. Next, UA buffer (8 M Urea, 150 mM Tris-HCl pH 8.0) was used to perform repeated ultrafiltration (Microcon units, 10 kD) for removing low-molecular-weight components, including detergent, DTT and so on. Then 100 µl iodoacetamide (100 mM IAA in UA buffer) was added to block reduced cysteine residues and the samples were incubated for 30 min in darkness. The filters were washed with 100 µl UA buffer three times and then 100 µl 25mM NH_4_HCO_3_ buffer twice. Finally, the protein suspensions were digested with 4 µg trypsin (Promega) in 40 µl 25mM NH_4_HCO_3_ buffer overnight at 37 °C, and the resulting peptides were collected as a filtrate. The peptides of each sample were desalted on C18 Cartridges (Empore™ SPE Cartridges C18 (standard density), bed I.D. 7 mm, volume 3 ml, Sigma), concentrated by vacuum centrifugation and reconstituted in 40 µl of 0.1% (v/v) formic acid. The peptide content was estimated by UV light spectral density at 280 nm using an extinctions coefficient of 1.1 of 0.1% (g/l) solution that was calculated on the basis of the frequency of tryptophan and tyrosine in vertebrate proteins.

For phosphopeptides enrichment, the enrichment of phosphopeptides was carried out using High-SelectTM Fe-NTA Phosphopeptides Enrichment Kit according to the manufacturer’s instructions (Thermo Scientific). After lyophilized, the phosphopeptides peptides were resuspended in 20 µL loading buffer (0.1% formic acid).

For LC-MS/MS analysis, the peptides were loaded on a C18-reversed phase analytical column (homemade, 25 cm long, 75 μm inner diameter, 1.9 μm, C18) in buffer A (0.1% Formic acid) and separated with a linear gradient of buffer B (84% acetonitrile and 0.1% Formic acid) at a flow rate of 300 nl/min. The mass spectrometer was operated in positive ion mode. The mass spectrometer collected ion mobility MS spectra over a mass range of m/z 100–1700 and 1/k0 of 0.6 to 1.6, and then performed 10 cycles of PASEF MS/MS with a target intensity of 1.5k and a threshold of 2500. Active exclusion was enabled with a release time of 0.4 min. Finally, The MS raw data for each sample were combined and searched using the MaxQuant software for identification and quantitation analysis. These identified DPPs are listed in Supplementary Data S4.

### Protein-protein docking analysis

Log in to the UniProt database (https://www.uniprot.org/), enter ‘TC2N’ or ‘DUSP3’ in the search box, and click ‘search’; Select the protein ID Q8N9U0 (TC2N) or P51452 (DUSP3) to access the corresponding protein information page; Click on the ‘Structure’ menu on the right side to download the protein files AF-Q8N9U0-F1 (TC2N) or 3F81 (DUSP3). Then, log in to the Z-dock website (http://zdock.umassmed.edu/), upload the AF-Q8N9U0-F1 and 3F81 files to the Input Protein 1 and Input Protein 2 text boxes, respectively, and click ‘submit’ to obtain the docking prediction results. Finally, import the predicted result files into the visualization website PDBePISA (https://www.ebi.ac.uk/msd-srv/prot_int/), and click ‘Launch PDBePisa’ for visual analysis. The estimated sequences of TC2N and DUSP3 interaction listed as Supplementary Data S5.

### Protein extraction and Western blot (WB) analysis

Cells or tissues were lysed in SDS lysis buffer (Beyotime, Shanghai, China) supplemented with PMSF (Beyotime, Shanghai, China) to extract total protein. The BCA quantification kit (Beyotime, Shanghai, China) was used to perform protein quantification. WB analysis was conducted according to the established protocols described previously [30]. Briefly, twenty micrograms of protein were run on 6% or 10% sodium dodecyl sulfate–polyacrylamide gel electrophoresis. The proteins on sodium dodecyl sulfate–polyacrylamide gel was transferred to polyvinylidene difluoride membrane (Millipore Corporation, Bedford, MA, USA). The membranes blocking with 5% milk for 2 h at room temperature with primary antibodies was incubated in incubator overnight at 4 °C. The proteins were incubated with the secondary antibody conjugated with horseradish peroxidase (HRP) and detected by chemiluminescence (Pierce, Rockford, IL, USA). Primary antibodies against TC2N (Cat. #: HPA027549) was purchased from Sigma-Aldrich. Flag (Cat. #:80010-1-RR and 66008-4-Ig), SOX2 (Cat. #: 11064-1-AP), OCT4 (Cat. #: 11263-1-AP), NANOG (Cat. #: 14295-1-AP), EGFR (Cat. #: 66455-1-Ig), ERK1/2 (Cat. #: 11257-1-AP), Phospho-ERK1/2 (Thr202/Tyr204) (Cat. #: 28733-1-AP), STAT3 (Cat. #: 10253-2-AP) and FAK1 (Cat. #: 12636-1-AP) were purchased from Proteintech Group (Wuhan, China). Phospho-STAT3 (Y705) (Cat. #: AP0070), Phospho-STAT3 (S727) (Cat. #: AP0705) and Phospho-FAK1 (Y576) (Cat. #: AP0536) were purchased from ABclonal Technology (Wuhan, China). DUSP3 (Cat. #: 4752), Phospho-EGFR (Y845) (Cat. #: 2231) and Phospho-EGFR (Y992) (Cat. #: 2235) were purchased from Cell Signaling Technology. Secondary antibodies (Cat. #: A0216 and A0208) were obtained from Beyotime. The WB experiment was repeated three times, and the original bands were uploaded as supplementary materials. Meanwhile, the quantitative and statistical analysis results of the WB bands are presented as Figure S3.

### Co-immunoprecipitation (Co-IP) assay

Immunoprecipitation assays were carried out using a protein A/G agarose bead (Beyotime, China) according to the manufacturer’s protocol. Briefly, total cell extracts were incubated with the indicated primary antibody and protein A/G agarose beads for 16 h at 4 °C. Next day, the immunoprecipitants were washed at least three times in SDS lysis buffer (Beyotime, Shanghai, China) and further being resolved by SDS-PAGE for WB analyses.

### DUSP3 phosphatase activity detection

For measuring the phosphatase activity of DUSP3, 10 µg of Flag-TC2N/Flag-CPNE1 and 5 µg of GST-DUSP3/GST-DUSP15 were incubated in binding buffer (50 mM Tris at pH 7.5, 5 mM MgCl_2_, 0.5 mM DTT, and 150 mM KCl) at 30 °C for 30 min to acquire purified proteins. After finishing the incubation, purified proteins were further incubated with 150 mM of the phosphatase substrate p-nitrophenyl phosphate (pNPP) (Sigma) dissolved in reaction buffer (1 M diethanolamine at pH 10.4 with 0.5 mM MgCl_2_) at 37 °C for 2 h. The changes of DUSP3 and DUSP15 phosphatase activity were assessed by analyzing the extent of hydrolyzation of the substrate at 405 nm using an ELISA instrument (Thermo Fisher).

### Statistical analysis

All replicates displayed in this paper are biological replicates; technical replicates (usually three) were performed and used to generate the means for each biological replicate. The sample sizes and number of replicates were indicated in the figure legends. Statistical analyses were performed using SPSS 19.0 software (SPSS, Inc., Chicago, IL, USA) and GraphPad Prism 9 software (La Jolla, CA, USA). All data were presented as means ± standard error of the mean (SEM). The differences between groups were assessed by Chi-square test, Student’s t-test (only two groups) or One-way ANOVA (three or four groups). The correlations were analyzed using Pearson and Spearman correlation test. Survival analyses were calculated by log-rank test Kaplan-Meier. Multivariate analysis of prognostic predictors was performed using Cox proportional hazard models. The p-values of < 0.05 was considered to be statistically significant.

## Results

### The knockout of TC2N delays urethane-induced lung tumorigenesis but does not have any observable impact on normal lung tissue

**Fig. 1:**
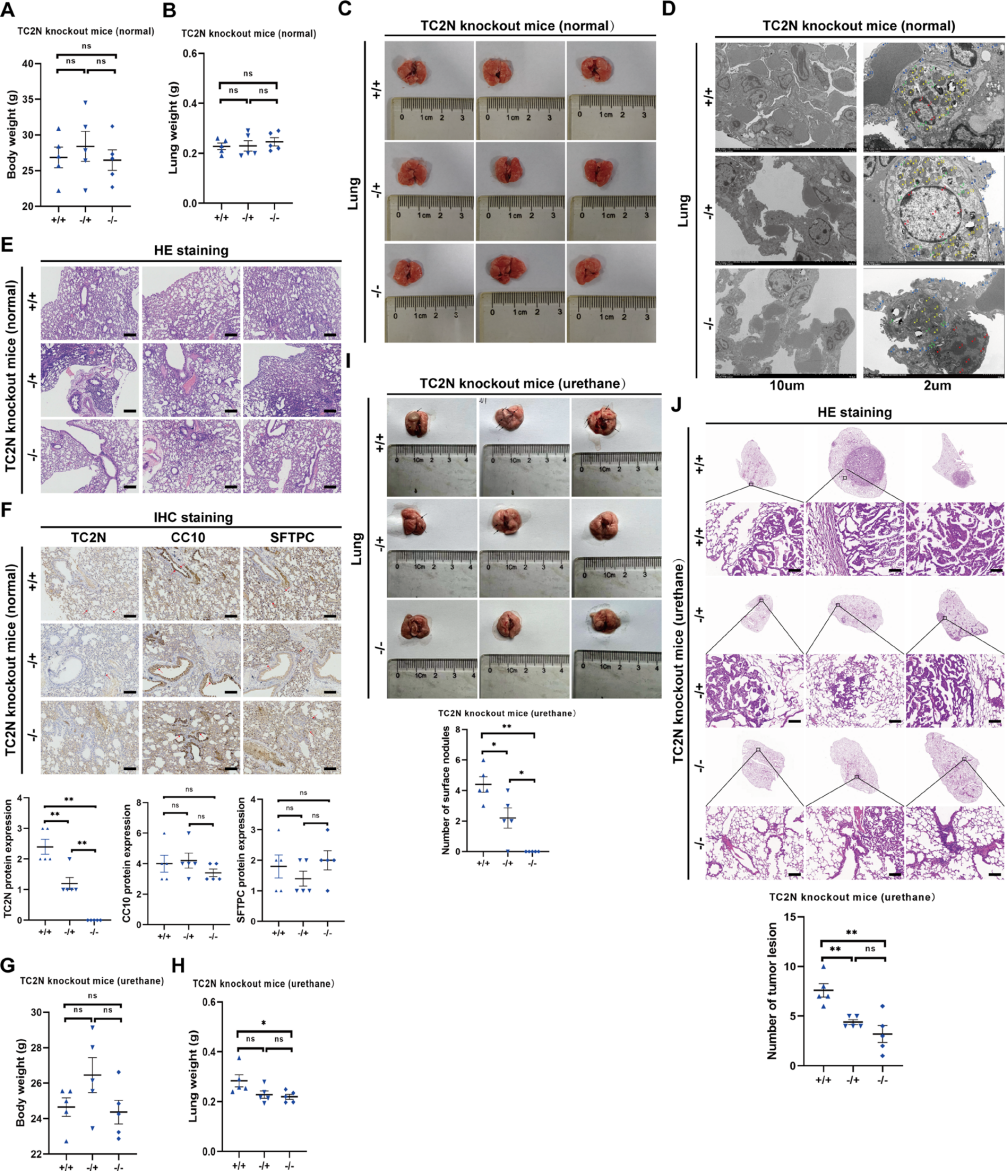
The knockout of TC2N delays urethane induced lung tumorigenesis but has no observable influence on normal lung. **A** Comparison of body weights from TC2N+/+, TC2N-/- and TC2N-/- mice. ns, no significance. **B** Comparison of lung weights from TC2N+/+, TC2N-/- and TC2N-/- mice. ns, no significance. **C** Lung tissues were exfoliated from 1 year old TC2N engineered mice and the photograph was taken. **D** Electron micrograph of the lung of different groups, showing no abnormalities. Red dot indicates cell; green dot indicates rough endoplasmic reticulum; yellow dot indicates mitochondria membrane; blue dot indicates the unbroken basement membrane. **E** Normal lung tissues were evaluated by HE staining in TC2N engineered mice. Scale bar represent 100 μm. **F** IHC evaluation of TC2N, CC10 and SFTPC expression in normal lung tissues of TC2N engineered mice without urethane exposure. Scale bar represent 100 μm. **G** Comparison of body weights from TC2N+/+, TC2N-/- and TC2N-/- mice with urethane exposure. ns, no significance. **H** Comparison of lung weights from TC2N+/+, TC2N-/- and TC2N-/- mice with urethane exposure. ns, no significance; *P < 0.05. **I** Lung tissues were exfoliated from TC2N engineered mice that injected with urethane. The photograph of lungs was taken. **J** Lung tumor was evaluated by HE staining in TC2N engineered mice (upper). Scale bar represent 100 μm. The number of tumor lesion in lung (lower). ns, no significance; **P < 0.01. Each group contains individual animal (*n* = 5)

To comprehensively understand the role of TC2N in normal lung physiology and lung tumorigenesis, we generated a TC2N knockout mouse model using CRISPR/Cas9 technology (Fig. S1A) and confirmed the genotypes of the mice through RT-PCR analysis (Fig. S1B). After being raised under normal conditions for one year, mice of each genotype were euthanized and sampled. The results showed no significant differences in body weight and lung tissue weight among the groups of mice (Fig. 1A and B). Upon general observation of lung tissues, all lungs exhibited a normal appearance with a bright red, smooth, and uniform surface (Fig. 1C). Ultrastructural analysis revealed that lung cells in each group displayed normal characteristics, including a normal cell nucleus (red dot) with even chromatin distribution, intact rough endoplasmic reticulum (green dot), intact mitochondrial membrane (yellow dot), unfractured crest, and an unbroken basement membrane (blue dot) without delamination (Fig. 1D). The pathological diagnosis results showed that no detectable lesions or spontaneous tumor were observed in the lung tissues of mice with different genotypes (Fig. 1E). Additionally, the expression of Clara cell marker CC10 (indicated by red arrows showing positive staining in bronchial epithelial cells) and type II alveolar epithelial cell marker SFTPC (indicated by red arrows showing positive staining in type II alveolar epithelial cells) was not affected as TC2N expression decreases (indicated by red arrows showing positive staining in the nuclei of normal lung cells, consistent with the previously reported subcellular localization in the nucleus [9, 31]) (Fig. 1F). These observations suggest that depletion of TC2N expression does not affect the normal physiological function of the lungs.

To investigate the pro-tumor activity of TC2N, we utilized urethane, an environmental carcinogen known to induce lung tumors in C57BL/6 mice [32], to establish a chemical carcinogenesis model. After a 6-month latency period following the last urethane injection, mice from each group were sacrificed for lung cancer assessment. Body weight differences among the groups were not significant (Fig. 1G), and there were no notable differences in lung weight except for slightly lower lung weight in TC2N-/- mice compared to TC2N+/+ mice on average (Fig. 1H). Gross observation of lung lesions revealed dark red lungs with rough surfaces, and the number of surface nodules in the TC2N+/+ group of mice was greater than that in the TC2N-/+ group, while no significant nodules were observed on the lung surfaces of the TC2N-/- group (Fig. 1I). Histological analysis of lung tissues stained with H&E showed that TC2N-/- mice exhibited smaller lung tumor nodules compared to TC2N-/+ and TC2N+/+ mice (Fig. 1J). Additionally, the mean number of tumor lesions decreased with TC2N depletion, although the differences between the -/+ and -/- groups were not statistically significant (Fig. 1J). These results indicate that TC2N plays a role in urethane-induced lung tumorigenesis but not in normal lung tissue. The underlying mechanisms by which TC2N promotes tumorigenesis and why it loses its function in normal lungs require further investigation.

### High TC2N expression is associated with the upregulation of stem cell markers in lung cancer, but not in normal lung tissue

**Fig. 2:**
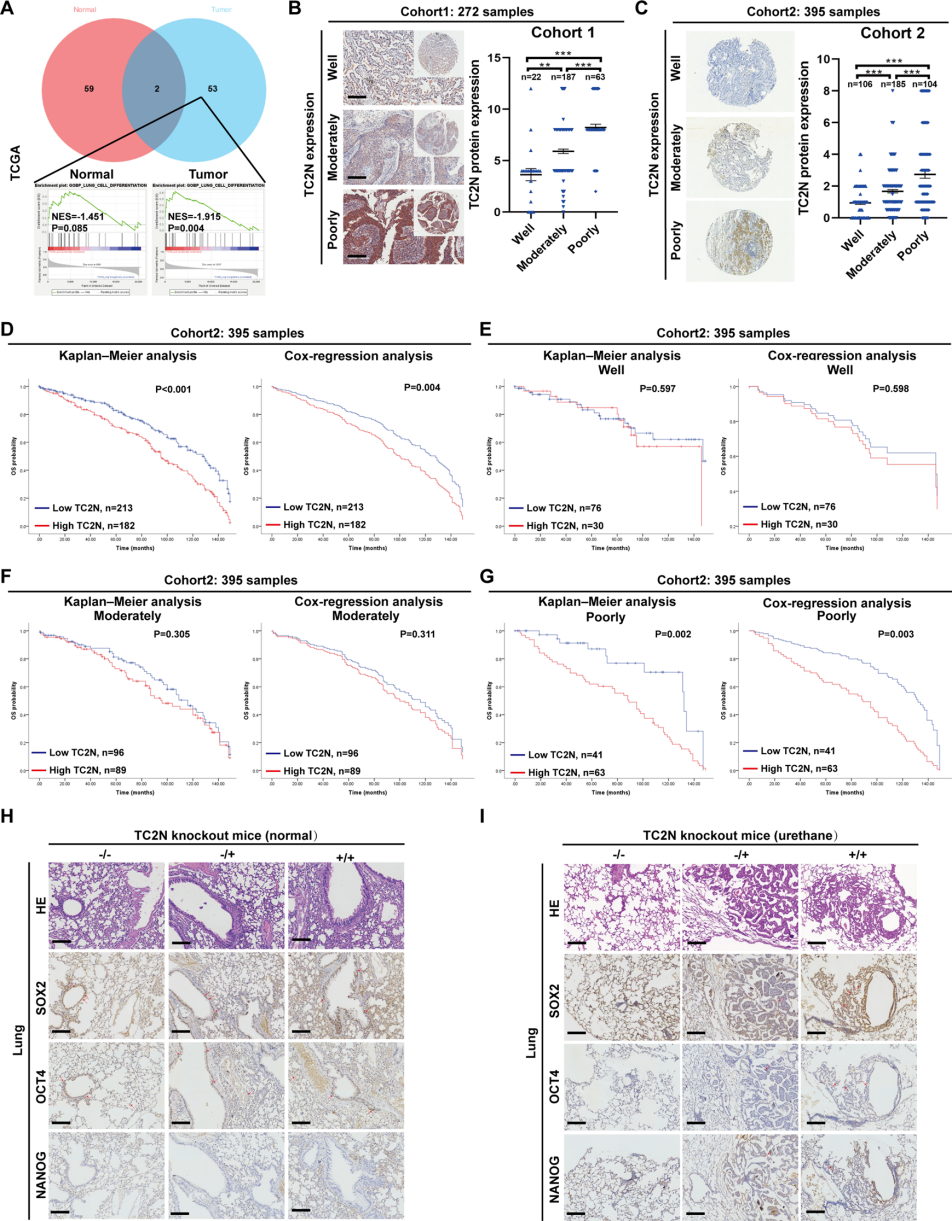
High TC2N expression represents the upregulated expression of stem cell markers in lung cancer, but not in normal lung. **A** Gene set enrichment analysis (GSEA) analysis of TCGA database revealed that TC2N was related to “LUNG CELL DIFFERENTIATION” process in lung cancer but not in normal lung. NES, Normalized Enrichment Score. **B** Cohort 1 showed that the protein expression of TC2N was associated to differentiation degree (well, moderately and poorly differentiated) of human lung tumor. Scale bar represent 100 μm. The P value was measured with Student’s t-tests. **P < 0.01, ***P < 0.001. **C** Cohort 2 verified that the protein expression of TC2N was associated to DD of human lung tumor. The P value was measured with Student’s t-tests. ***P < 0.001. **D** Kaplan–Meier and COX regression analyses showed that TC2N expression was associated with poor prognosis of lung cancer patients. **E** Survival analysis of TC2N expression in lung cancer patients with well tumor differentiation by Kaplan–Meier and COX regression analyses. **F** Survival analysis of TC2N expression in lung cancer patients with moderately tumor differentiation by Kaplan–Meier and COX regression analyses. **G** Survival analysis of TC2N expression in lung cancer patients with poorly tumor differentiation by Kaplan–Meier and COX regression analyses. **H** IHC evaluation of SOX2, OCT4 and NANOG expression in normal lung tissues of TC2N engineered mice without urethane exposure. Scale bar represent 100 μm. **I** IHC evaluation of SOX2, OCT4 and NANOG expression in lung tissues of TC2N engineered mice with urethane exposure. Scale bar represent 100 μm

To provide a reasonable explanation for the functional differences of TC2N between normal lung and tumors, we conducted Gene Set Enrichment Analysis (GSEA) using the TCGA database. The analysis revealed significant variations in TC2N-related biological processes between adjacent non-cancerous lung tissues and lung tumor tissues (Fig. 2A, Supplementary Data S2). Notably, the top-ranked biological process in the tumor group was “GOBP_LUNG_CELL_DIFFERENTIATION” (*P* = 0.004), while there was no significant difference in the non-cancerous group (*P* = 0.085) (Fig. 2A). This suggests that TC2N expression may be associated with the degree of tumor differentiation. To further investigate the relationship between TC2N expression and tumor differentiation in a clinical context, we performed a retrospective analysis on our previous Tissue Microarray (TMA) cohort (Cohort 1) consisting of 272 human lung tumor tissues [9]. As shown in Fig. 2B, TC2N expression significantly increased with decreasing tumor differentiation. These results were confirmed in Cohort 2 using another TC2N antibody, where TC2N expression was observed to increase with decreasing tumor differentiation (Fig. 2C). Subsequent Kaplan-Meier and COX regression analyses based on Cohort 2 demonstrated that high TC2N expression was associated with poor prognosis in human lung cancer (Fig. 2D). Additionally, we analyzed the association between TC2N expression and patient survival in different tumor differentiation groups. The results indicated that high TC2N expression was correlated with poor prognosis in patients with poorly differentiated lung tumors, but not in patients with well or moderately differentiated lung tumors (Fig. 2E and G). Given that cancer progression involves the loss of differentiation and acquisition of stem cell-like features [33], we examined the effect of TC2N expression on three stem cell markers (SOX2, OCT4, and NANOG) in normal lung (without urethane treatment) and urethane-induced lung tumor tissues. In normal mice lungs, there were no significant differences in the expression of stem cell markers (SOX2 expression indicated by red arrows showing nuclear staining in bronchus; OCT4 expression indicated by red arrows showing nuclear staining in bronchus and alveolus; No significant NANOG positive staining was observed in all groups) among the three genotypes (Fig. 2H). In urethane-induced lung tumors, nuclear positivity of SOX2, OCT4, and NANOG was observed in some cells in both TC2N+/+ and TC2N-/+ tumor groups (red arrows), while no significant staining signal was observed in the TC2N-/- tumor group (Fig. 2I). These findings suggest that TC2N may play a role in maintaining stemness in lung tumors.

### TC2N plays a positive role in modulating the stem cell-like phenotype in lung cancer cells

**Fig. 3:**
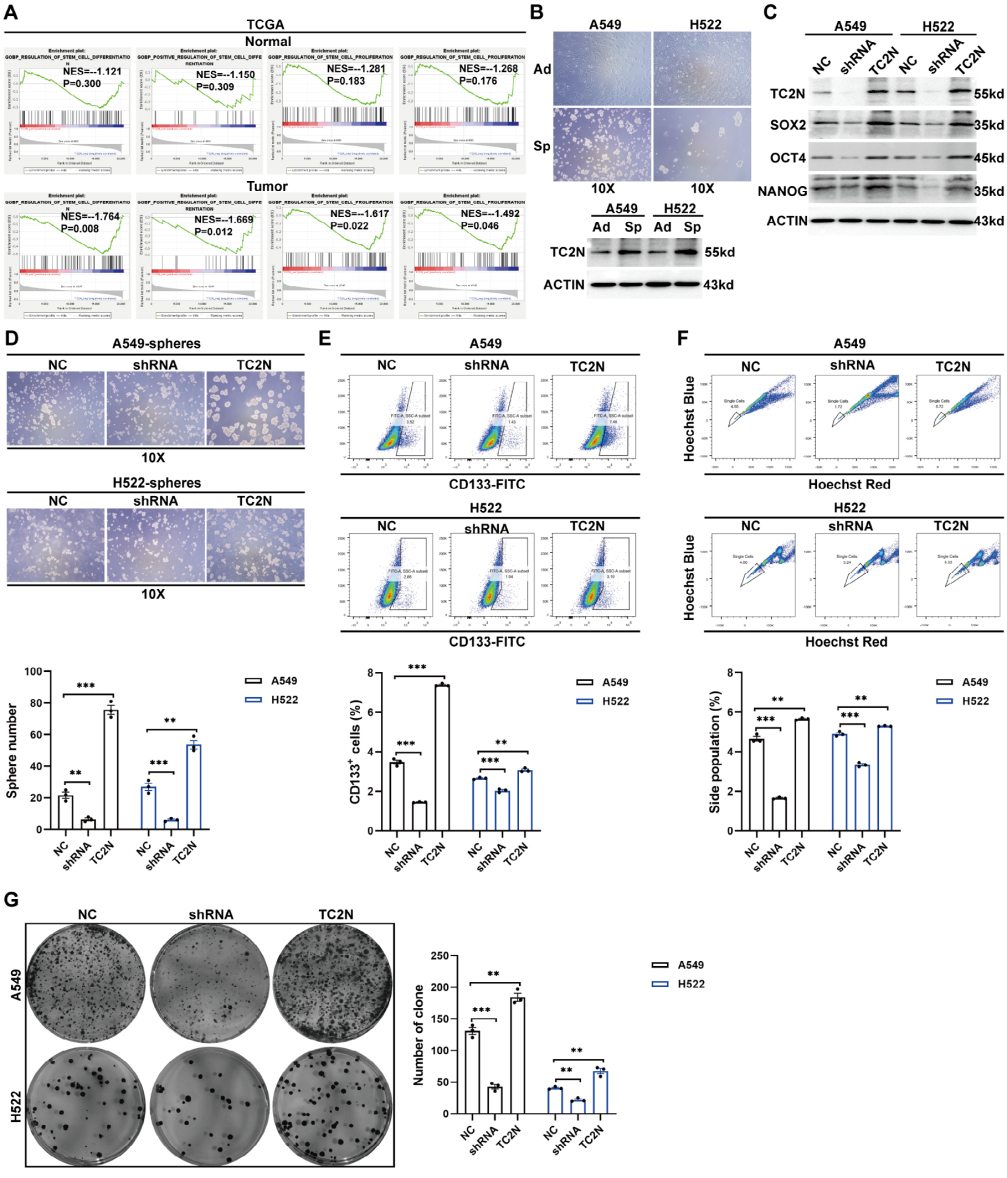
TC2N upregulates the CSC-like characteristics of lung cancer cells. **A** Gene set enrichment analysis (GSEA) analysis of TCGA database revealed that TC2N was related to “STEM CELL” relevant processes in lung cancer but not in normal lung. NES, Normalized Enrichment Score. **B** Cellular morphology of adherent lung cancer cells (Ad) compared to spheroid-forming lung cancer cells (Sp). Scale bars represent 120 μm (upper). WB analysis of TC2N expression in adherent and spheroid‐forming lung cancer cells (lower). **C** WB analysis of SOX2, OCT4 and NANOG expression in stable lung cancer cells. **D** The sphere formation ability of lung cancer cells with TC2N overexpression (TC2N group) and knockdown (shRNA group) was compared to that of the negative control (NC group). Scale bars represent 120 μm. Mean ± SEM. (*n* = 3). The P value was measured with Student’s t-tests. *P < 0.05, **P < 0.01. **E** The expression of CSCs marker CD133 (APC) in lung cancer cells with ectopic expression of TC2N (NC, shRNA and TC2N groups) were analyzed by flow cytometry. Mean ± SEM. (*n* = 3). The P value was measured with Student’s t-tests. **P < 0.01, ***P < 0.001. **F** The proportion of side population cells in lung cancer cells with ectopic expression of TC2N (NC, shRNA and TC2N groups) were analyzed by flow cytometry. Mean ± SEM. (*n* = 3). The P value was measured with Student’s t-tests. **P < 0.01, ***P < 0.001. **G** Soft agar colony formation ability of spheroid‐forming lung cancer cells with ectopic expression of TC2N (NC, shRNA and TC2N groups). Mean ± SEM. (*n* = 3). The P value was measured with Student’s t-tests. **P < 0.01, ***P < 0.001. The cell experiments were repeated 3 times independently

The aforementioned results have prompted us to further investigate the underlying role of TC2N in cancer stem cell-like characteristics. GSEA analysis of the TCGA database revealed significant differences in the enrichment of relevant biological processes related to STEM CELL between adjacent non-cancerous tissues and tumor tissues (Supplementary Data S2). As shown in Fig. 3A, the expression of TC2N in tumors, but not in normal tissues, was associated with multiple stem cell-related biological processes, primarily involving stem cell differentiation and proliferation. To validate this finding, we screened for genes with similar expression patterns to TC2N using the TCGA public database and five large sample GEO datasets (Supplementary Data S1) and performed GO analysis. Consistently, the co-expressed genes of TC2N were enriched in multiple “CELL DIFFERENTIATION” and “STEM CELL” processes (Fig. S2). Considering that we aim to elucidate the role of TC2N in tumor origin and that CSCs are key to tumor occurrence, we explored the relationship between TC2N expression and tumorigenesis in a CSC stemness point of view. Due to the known histological type of uranium-induced lung cancer being lung adenocarcinoma, we selected two lung adenocarcinoma cell lines, A549 and H522, for biological functional analysis in order to better replicate in vivo functionality. These two cell lines were cultured in serum-free suspension medium for 5 days to induce spheroids (Fig. 3B) and then the TC2N expression was detected. We observed that TC2N protein levels were upregulated in A549 and H522 cell spheres (Sp) compared with adherent cells (Ad) (Fig. 3B), hinting a potential role in CSC. We then utilized the same lentivirus to construct both overexpression and interference cell models in A549 and H522 cells to further evaluate the impact of differentially expressed TC2N on cell stemness. Subsequently, we assessed the expression levels of stem cell markers SOX2, OCT4 and NANOG in these cells. Consistently, silencing of TC2N significantly reduced the expression of these markers, which could be upregulated by overexpression of TC2N (Fig. 3C). Subsequently, we conducted spheroid formation assays, and the results showed that silencing TC2N in A549 and H522 cells significantly reduced both the number and size of spheres, whereas upregulation of TC2N in these cells resulted in the formation of much larger and significantly more spheres (Fig. 3D). This indicates that TC2N can promote spheroid formation and enhance the self-renewal capacity of the cells. Meanwhile, a lung CSC marker CD133 was monitored by flow cytometry analysis. The results showed that the percent of CD133-positive cells were decreased in cells with TC2N knockdown, and were increased in cells with TC2N overexpression (Fig. 3E). Furthermore, the side populations of A549 and H522 stable transfectants, which represented CSC-like cell populations were observed. As expected, cells with high expression of TC2N exhibited a higher proportion of side population cells, whereas cells with low expression of TC2N showed a decreased proportion of side population cells (Fig. 3F). Consistently, a cloning formation assay was performed to further evaluate the self-renewal capacity of CSCs, revealing that cell spheres with high TC2N expression formed a greater number of clones compared to those with low TC2N expression. (Fig. 3G). These observations indicated that TC2N plays a crucial role in maintaining the stemness of lung cancer cells.

### TC2N changes the phosphorylation status of multiple oncogenic proteins in lung cancer but not in normal lung

**Fig. 4:**
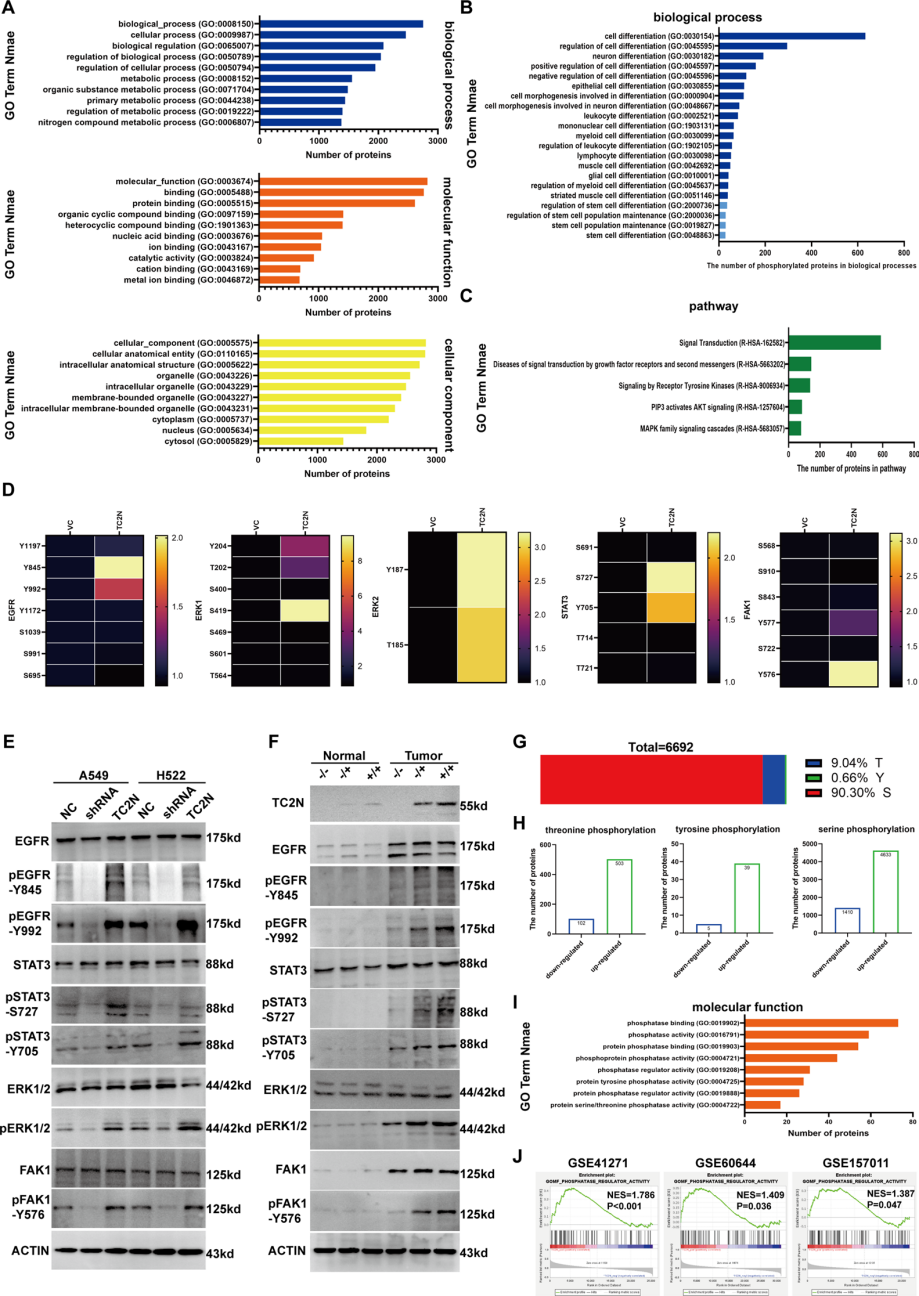
Analyzing the potential signaling pathways of TC2N in regulating cancer cell stemness. **A** The Gene ontology (GO) enrichment diagrams for the top ten biological processes (top), molecular functions (middle), and cellular components (bottom) enriched with differentially phosphorylated peptides (DPPs). **B** The GO enrichment diagrams for the stem cell and differentiation-related biological processes enriched with DPPs. **C** The GO enrichment diagrams for the stemness-related signaling pathways enriched with DPPs. **D** Phosphoproteomics identified the changed phosphorylation sites of EGFR, ERK1, ERK2, STAT3 and FAK1 after TC2N overexpression. **E** The phosphorylation levels of EGFR, STAT3, ERK1/2 and FAK1 were monitored by WB after knockdown or overexpression of TC2N in A549 and H522 cells. **F** The phosphorylation levels of EGFR, STAT3, ERK1/2 and FAK1 were monitored by WB in normal lung and lung tumor of TC2N engineered mice. **G** The percentage of threonine, tyrosine and serine residues of DPPs after TC2N overexpression. **H** The number of threonine, tyrosine and serine phosphorylation of proteins after TC2N overexpression. **I** Phosphoproteomics identified the phosphatase-related molecular functions. **J** Gene set enrichment analysis (GSEA) analysis of GEO datasets revealed that TC2N was related to “PHOSPHATASE” relevant processes in lung cancer. NES, Normalized Enrichment Score

The dysregulation of protein phosphorylation occurs during tumor initiation [34] and affects signaling cascades in cancer [35]. In our previous studies, we have uncovered the important role of TC2N in regulating the phosphorylation of several proteins, including p53, p65, p50, p55γ, and Akt [9–11]. To investigate this, we performed mass spectrometry-based phosphoproteomics analysis on A549 cells with TC2N overexpression. A total of 6,692 differentially phosphorylated peptides (DPPs) were identified (Supplementary Data S4). The enriched Gene Ontology (GO) biological processes, molecular functions, and cellular components of these DPPs are shown in Fig. 4A. Upon further analysis, we found that these DPPs were indeed associated with multiple stem cell processes (Fig. 4B). Additionally, these DPPs were enriched in various signaling pathways, including signal transduction, receptor tyrosine kinase, PIP3/Akt and MAPK cascades (Fig. 4C), which are highly correlated with stemness [36–39]. Notably, the phosphoproteomics analysis revealed that key sites of proteins such as EGFR, ERK1, ERK2, STAT3 and FAK1 were hyperphosphorylated upon TC2N overexpression (Fig. 4D). Western blot assays confirmed that TC2N depletion led to a decrease in the phosphorylation of these proteins, while TC2N overexpression increased their phosphorylation levels (Fig. 4E). In TC2N knockout mice, TC2N depletion inhibited the phosphorylation levels of these sites in urethane-induced lung tumors, but not in normal lungs without urethane treatment (Fig. 4F). Among the 6,692 DPPs, the proportion of phosphorylated threonine, tyrosine, and serine residues was 9.04%, 0.66%, and 90.30%, respectively (Fig. 4G). Furthermore, the number of upregulated phosphorylated proteins was higher than the number of downregulated phosphorylated proteins, regardless of residue types (Fig. 4H). Since TC2N does not possess a known kinase domain, we speculated that TC2N might regulate protein phosphorylation in a phosphatase-dependent manner. GO analysis revealed that the molecular function of the DPPs was enriched in multiple patterns related to “phosphatase activity” and “phosphatase binding” (Fig. 4I). These findings were further supported by GSEA analysis of three large independent sample GEO datasets (Fig. 4J, Supplementary Data S3).

### TC2N interacts with DUSP3 to block its hydrolysis

**Fig. 5:**
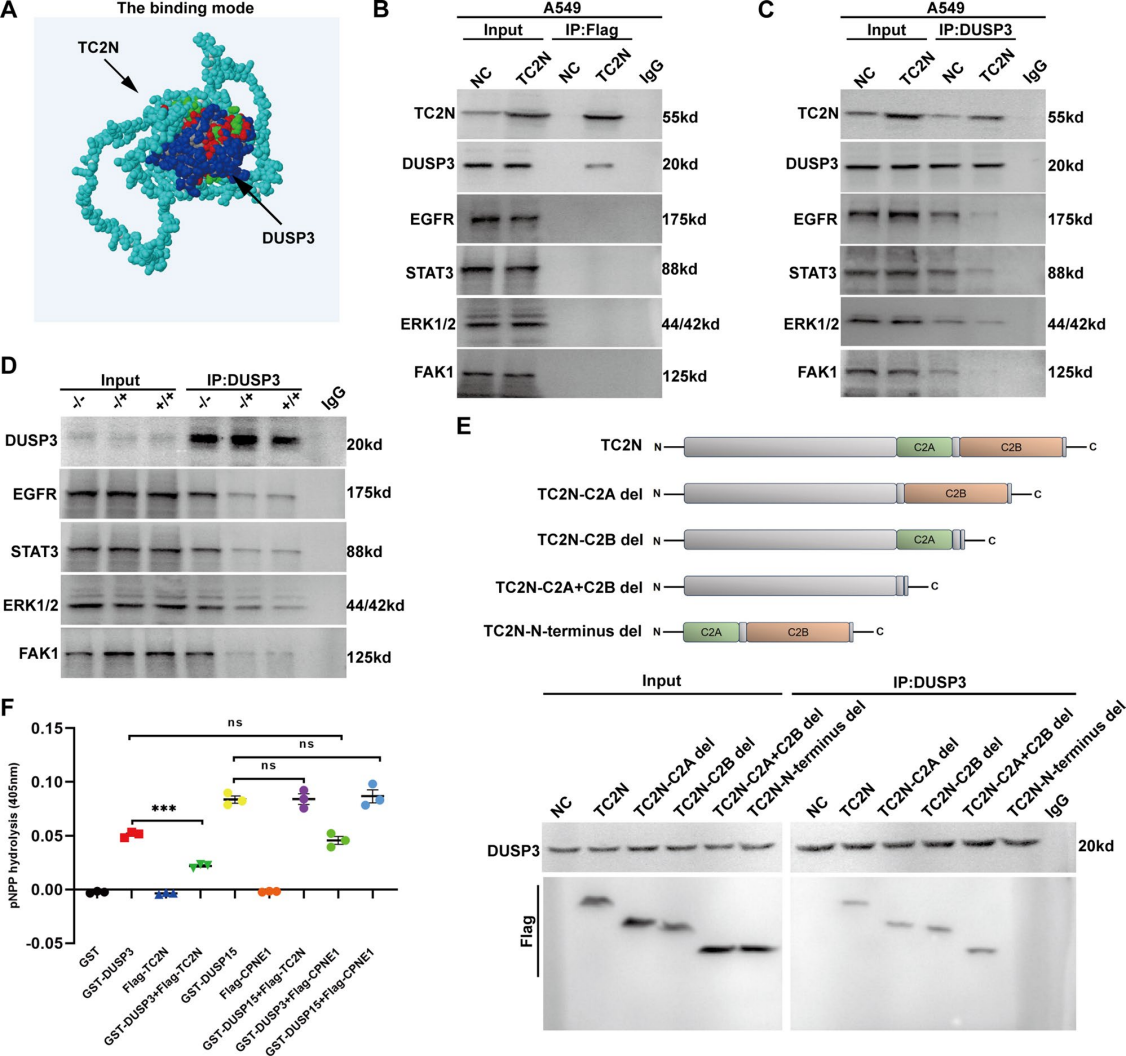
TC2N interacts with and blocks dual specificity protein phosphatase 3 to enhance CSC-like properties of lung cancer. **A** Z-dock mode of TC2N identified an interaction surface on DUSP3. The TC2N was represented in light blue; DUSP3 in dark blue. **B** The extracts from lung cancer cells were subjected to IP with anti-Flag antibody and further analyzed by WB with indicated antibodies. Normal IgG was used as a negative control. Whole-cell lysates were used as a positive control (Input). **C** The cell extracts from lung cancer cells were subjected to IP with anti-DUSP3 antibody and further analyzed by WB with indicated antibodies. Normal IgG was used as a negative control. Whole-cell lysates were used as a positive control (Input). **D** The extracts from lung tumor of TC2N engineered mice were subjected to IP with anti-DUSP3 antibody and further analyzed by WB with indicated antibodies. Normal IgG was used as a negative control. Whole-cell lysates were used as a positive control (Input). **E** The extracts from lung cancer cells stably expressing Flag-NC or Flag-TC2N (full-length and truncation) were subjected to IP with anti-DUSP3 antibody and further analyzed by WB with Flag antibodies. Normal IgG was used as a negative control. Whole-cell lysates were used as a positive control (Input). **F** In vitro phosphatase assay was performed to detect DUSP3 phosphatase activity using p-nitrophenyl phosphate (pNPP). values are shown as Mean ± SEM. (*n* = 3). Student’s t-tests, ns, no significance; ***P < 0.001

In previous studies, we identified dual specificity protein phosphatase 3 (DUSP3) as a potential interactor of TC2N [11]. DUSP3 is known to interact with and dephosphorylate proteins such as EGFR, ERK1, ERK2, STAT3, and FAK1 through its phosphatase activity [40–42]. We hypothesized that TC2N may enhance the phosphorylation of these proteins by blocking DUSP3 in lung cancer cells. After obtaining the protein sequences of TC2N and DUSP3, we conducted a protein docking analysis, which revealed that TC2N can interact with and wrap around DUSP3 (Fig. 5A). Subsequent immunoprecipitation (IP) assays confirmed the interaction between TC2N and DUSP3, but not with the aforementioned substrate proteins of DUSP3 (Fig. 5B), raising the possibility that TC2N might interact with DUSP3 to interfere with substrate for binding to DUSP3. Co-IP assays using DUSP3 antibody in stable lung cancer cell lines showed that TC2N overexpression reduced the interaction between endogenous DUSP3 and its substrates (Fig. 5C). These results were further confirmed in the urethane-induced lung tumor model, where TC2N deletion increased the interactions between endogenous DUSP3 and its substrates in lung tumor tissues of mice (Fig. 5D). To determine the key domain of TC2N responsible for its interaction with DUSP3, co-IP assays were performed using full-length TC2N and its truncations (C2A del, lack C2A domain of TC2N; C2B del, lack C2B domain of TC2N; C2A + C2B del, lack C2A and C2B domain of TC2N; N-terminus del, lack N-terminus of TC2N) tagged with Flag. The results indicated that all forms of TC2N could interact with DUSP3, except for the N-terminus deletion variant (Fig. 5E). This suggests that the key domain or segment responsible for the interaction between TC2N and DUSP3 is likely located at the N-terminus of TC2N. To evaluate the impact of TC2N on the activity of DUSP3, we conducted enzymatic activity assays using p-nitrophenyl phosphate (pNPP) as a substrate. The results demonstrated that TC2N could inhibit the hydrolytic activity of DUSP3 towards NPP, while it did not affect the hydrolytic activity of DUSP3’s homolog, DUSP15, towards nPP (Fig. 5F). Additionally, TC2N’s homolog, CPNE1, showed no significant impact on the hydrolytic activity of both DUSP3 and DUSP15 (Fig. 5F). These findings indicate that TC2N’s inhibitory effect on DUSP3 hydrolytic activity is specific.

### DUSP3 is the functional downstream target through which TC2N promotes the stemness of lung cancer cells

**Fig. 6:**
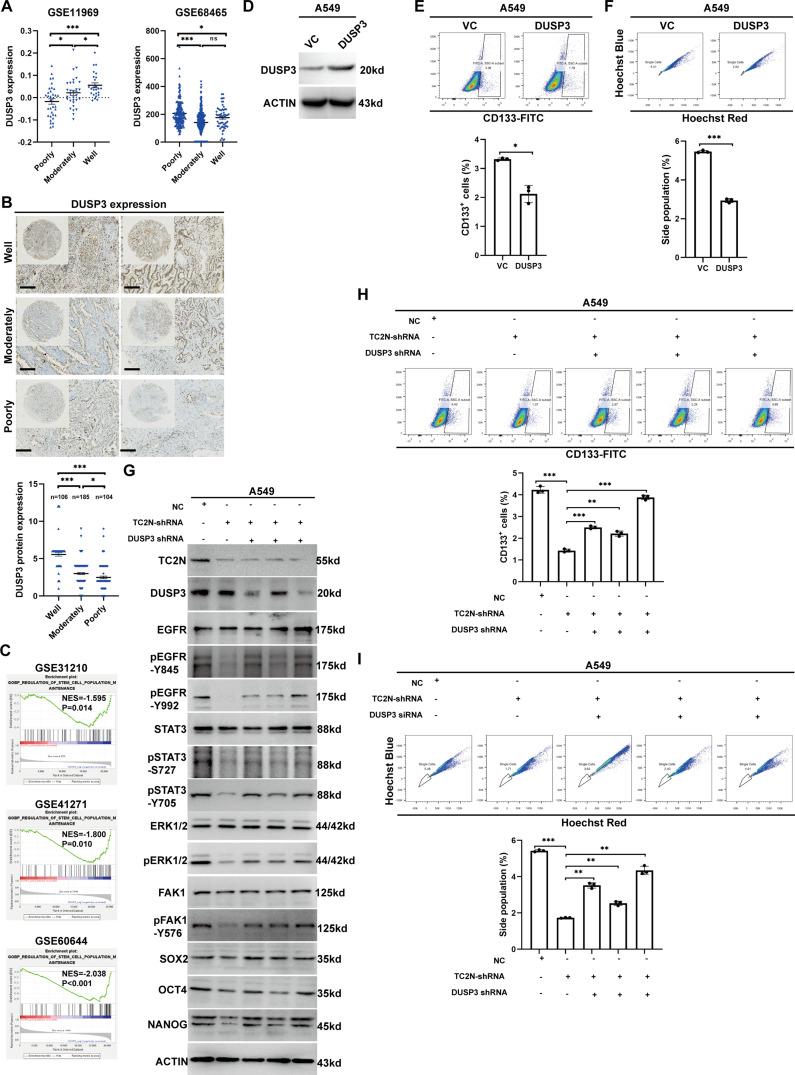
TC2N promotes the stemness of lung cancer cells by inhibiting DUSP3. **A** DUSP3 mRNA expression data in lung cancer tissues at different differentiation degree from GSE11969 and GSE68465 datasets. The P value was measured with Student’s t-tests. ns, no significance; *P < 0.05, ***P < 0.001. **B** DUSP3 protein expression data in lung cancer tissues at different differentiation degree from tissue microarray (TMA). The P value was measured with Student’s t-tests. *P < 0.05, ***P < 0.001. **C** Gene set enrichment analysis (GSEA) analysis of GEO datasets revealed that DUSP3 was related to stem cell-related processes in lung cancer. NES, Normalized Enrichment Score. **D** WB assays were conducted to examine the expression of DUSP3 in A549 cells with DUSP3 overexpression. **E** The proportion of CD133^+^ cells were examined to evaluate the effect of DUSP3 overexpression on self-renewal of lung cancer cells. Mean ± SEM. (*n* = 3). The P value was measured with Student’s t-tests. *P < 0.05. **F** The proportion of side population cells were examined to evaluate the effect of DUSP3 overexpression on self-renewal of lung cancer cells. Mean ± SEM. (*n* = 3). The P value was measured with Student’s t-tests. ***P < 0.001. **G** WB assays were conducted to examine the effect of DUSP3 ablation, using three independent shRNA fragments, on the phosphorylation of EGFR, ERK1, ERK2, STAT3, and FAK1, as well as the expression of stem cell markers in lung cancer cells with TC2N knockdown. **H** The proportion of CD133^+^ cells were examined to evaluate the effect of DUSP3 ablation (DUSP3 shRNA) on self-renewal of lung cancer cells with TC2N knockdown. Mean ± SEM. (*n* = 3). The P value was measured with Student’s t-tests. **P < 0.01, ***P < 0.001. **I** The proportion of side population cells were examined to evaluate the effect of DUSP3 ablation (DUSP3 shRNA) on self-renewal of lung cancer cells with TC2N knockdown. Mean ± SEM. (*n* = 3). The P value was measured with Student’s t-tests. **P < 0.01, ***P < 0.001

Analysis of two independent large sample lung cancer GEO datasets (GSE11969 and GSE68465) showed that DUSP3 expression was lower in well-differentiated tumors compared to poorly-differentiated tumors (Fig. 6A). This result was further validated by immunohistochemistry (IHC) analysis of DUSP3 protein expression in human lung cancer tissues from Cohort 2 TMA, which included well-differentiated, moderately differentiated, and poorly differentiated lung tumors. The results showed a decrease in DUSP3 expression in the order of well, moderately, and poorly differentiated tumor groups (Fig. 6B). Furthermore, GSEA analysis based on GSE31210, GSE41271, and GSE60644 confirmed the association of DUSP3 with “stem cell population maintenance” (Fig. 6C, Supplementary Data S3). To verify whether DUSP3 has a role in regulating cell stemness, we overexpressed DUSP3 in A549 cells (Fig. 6D) and assessed the proportions of CD133^+^ and side population cells using flow cytometry. We found that the upregulation of DUSP3 significantly reduced the proportions of CD133^+^ and side population cells (Fig. 6E and F). These findings suggest that DUSP3 serve as a candidate inhibitor of stemness.

Having established that TC2N is an upstream regulator of DUSP3, we conclude that TC2N plays a critical role in maintaining stemness by blocking DUSP3 using three independent shRNA fragments. As expected, interference with the DUSP3 expression abrogated the TC2N knockdown-induced dephosphorylation of EGFR, ERK1/2, STAT3 and FAK1, thereby restoring the expression of SOX2, OCT4, and NANOG in A549 cells (Fig. 6G). Moreover, DUSP3 silencing significantly reversed the inhibitory effects of TC2N knockdown on the proportion of CD133-positive cells and side population cells (Fig. 6H and I). These findings collectively suggest that TC2N promotes lung tumor progression by blocking DUSP3 and activating multiple oncogenic signaling pathways.

## Discussion

In order to explore whether TC2N involves in lung tumor initiation, a TC2N knockout mice model was developed and used to examine normal lung lesion or lung tumor. In both macro and micro aspects, we did not notice any lung injury and tumor formation in TC2N knockout mice. Does that mean TC2N has no impact on tumorigenesis? Contradictorily, we previously found that TC2N overexpression could accelerate cell proliferation of HBE, a human bronchial epithelial cell line [9]. As hyperproliferation is one of hallmarks of cancer cell [43], this side-fact indicate the potential effects of TC2N in tumorigenesis. Previously, we compared the expression of TC2N between the bronchial epithelial cell line HBE and multiple lung cancer cell lines and observed that TC2N expression was lower in HBE compared to the cancer cell lines [9]. Based on the extremely low expression of TC2N in normal lung tissue and adjacent non-cancerous tissue, we speculate that a sufficient level of TC2N expression or carcinogenic stimuli may be required to drive tumor initiation. Similarly, the lack of significant changes in stem cell marker expression in normal lung tissue after TC2N knockout may also be attributed to the low basal expression of TC2N. We theorize that only TC2N-knockin mice would exhibit spontaneous lung tumor formation, not the TC2N-knockout mice. In our subsequent studies, we plan to generate TC2N transgenic mice to further evaluate whether overexpression of TC2N can drive spontaneous lung tumor formation.

To evaluate the carcinogenic potential of TC2N, we used urethane, a tumor inducer, and found that TC2N knockout mice were more resistant to urethane-induced lung tumorigenesis compared to TC2N heterozygous and wild-type mice. In parallel with these results, deficiency in TC2N executed a negative effect on stem cell marker (SOX2, OCT4 and NANOG) expression in murine lung tumor. In vitro experiments have also confirmed that overexpression of TC2N can upregulate the expression of SOX2, OCT4 and NANOG, thereby promoting stemness. It is noteworthy that the analysis results based on the TCGA and GEO databases indicated that TC2N is associated not only with stemness but also with the processes of cell differentiation. Furthermore, histological results indicated that TC2N expression is low in well-differentiated tumor tissues and high in poorly differentiated tumor tissues, seeming to suggest a potential role for TC2N in regulating cell differentiation. However, this differentiation discrepancy involves two opposing processes: the differentiation of stem cells into tumor tissues and the dedifferentiation of differentiated tumor tissues back into stem cells [44]. Therefore, we cannot determine whether the current results are due to TC2N’s involvement in dedifferentiation or its inhibition of stem cell differentiation. Considering that both processes involve CSCs, we chose to analyze the role of TC2N in tumorigenesis from this perspective, leaving the question of whether TC2N regulates cell differentiation unclear. Certainly, we cannot exclude the potential role of TC2N in tumor differentiation/dedifferentiation. Moreover, the stemness of CSCs and the level of differentiation are inherently closely related factors; it is generally accepted that higher stemness correlates with lower differentiation degree, while lower stemness is associated with higher differentiation degree [45]. Therefore, we will conduct further research on this aspect in future studies.

How does TC2N regulate the expression of SOX2, OCT4 and NANOG? Through phosphoproteomics analysis, we have discovered that overexpression of TC2N leads to the enhanced phosphorylation of proteins such as EGFR, ERK, STAT3, and FAK1, which have been demonstrated to directly or indirectly promote the expression of stem cell markers, including SOX2, OCT4, and NANOG [46–49]. Actually, EGFR, ERK, STAT3, and FAK1 are also dominant players in tumorigenesis [50–54], we supported that TC2N upregulated phosphorylation levels of these oncoproteins, which in turn conferred oncogenic signals to promote tumor initiation. However, the mechanism involved needs to be clarified. DUSP3 phosphatase, also known as Vaccinia H1-Related Phosphatase (VHR), belongs to the dual-specificity protein phosphatases (DUSPs) family and is involved in dephosphorylating p-threonine (p-Thr), p-serine (p-Ser), and p-tyrosine (p-Tyr) residues of target proteins [55]. Because of its shallow and broad catalytic pocket, DUSP3 has a diversity of substrates and acts as a central regulator of a variety of biological processes, including cancer [56]. The emerging evidence suggested that this enzyme has double-faced role in different types of cancers. In prostate cancer and cervix cancer, DUSP3 serves as tumor-promoting phosphatase [27, 57, 58], oppositely, its execute a tumor suppressor function in breast and lung cancer [40]. Therefore, its role in cancer is complex and context-dependent. Regarding the mechanism of the tumor-promoting role of TC2N in lung cancer, we showed that the interaction between DUSP3 and TC2N blocked DUSP3’s interaction with its substrates and inhibited its enzymatic activity. To preliminarily analyze the potential interaction between TC2N and DUSP3, we conducted protein docking analysis to predict their potential binding sites. The results showed that TC2N can bind extensively and even envelop DUSP3. Meanwhile, the IP results indicated that the deletion of the C2A and/or C2B domains of TC2N does not impact its interaction with DUSP3, suggesting that these two domains are not essential for their binding. Instead, the N-terminus of TC2N plays a crucial role in facilitating this interaction. However, according to previous literature, the specific domains present in the N-terminus of TC2N have not been identified, and therefore, we are currently unable to elucidate the exact binding mode between TC2N and DUSP3. Based on the docking data and IP results, we speculate that TC2N may block the hydrolytic activity of DUSP3 through two possible mechanisms: (1) The N-terminus of TC2N contains a protein domain, such as a phosphatase domain, that can bind to and induce the dephosphorylation of residue 138 in DUSP3 (Supplementary Data S5), which is an active site activated by phosphorylation modification [59]; Our reasoning for this speculation is that C2 domains typically do not exist in isolation; many kinases and phosphatases contain C2 domains, which are crucial for their activity. Therefore, TC2N may also be a protein belonging to the kinase or phosphatase family. (2) The N-terminus of TC2N lacks a protein domain and acts merely as a molecular chaperone to encapsulate DUSP3, thereby preventing its interaction with substrates.

Before then, the previous research has shown that DUSP3 can regulate cell differentiation [60], but there has been no report on its connection with CSCs. In current study, we speculated that DUSP3 is involved in CSC-related processes based on the association between DUSP3 expression and lung tumor DD. Parallelly, we demonstrated that the inhibition of DUSP3 is crucial for TC2N to exert its role in promoting the stemness of lung cancer cells by either overexpressing DUSP3 alone in original cells or interfering with DUSP3 expression in TC2N-silenced cells. Interestingly, after interfering with DUSP3 expression, the phosphorylation levels of EGFR, STAT3, ERK1/2, and FAK1 did not fully recover (Fig. 6G), and the stemness of the cells was only partially restored (Fig. 6H and I). This may be due to TC2N influencing the phosphorylation of these molecules through other non-DUSP3-dependent mechanisms, or TC2N affecting cell stemness via other signaling pathways. Indeed, PI3K/Akt, WNT/β-catenin, NOTCH, Hedgehog signaling pathways and other phosphorylated proteins also contribute to stemness of cancer cell [61]. In this study, the impact of TC2N ectopic expression on phosphorylated proteins cannot be fully explained by DUSP3 alone. Based on the structural features of TC2N, we speculated that TC2N acts as a molecular chaperone, orchestrating or assisting other client proteins in modulating multiple intracellular signaling pathways. Therefore, we plan to focus our future work on investigating the protein structure and function of TC2N.

## Conclusions

**Fig. 7:**
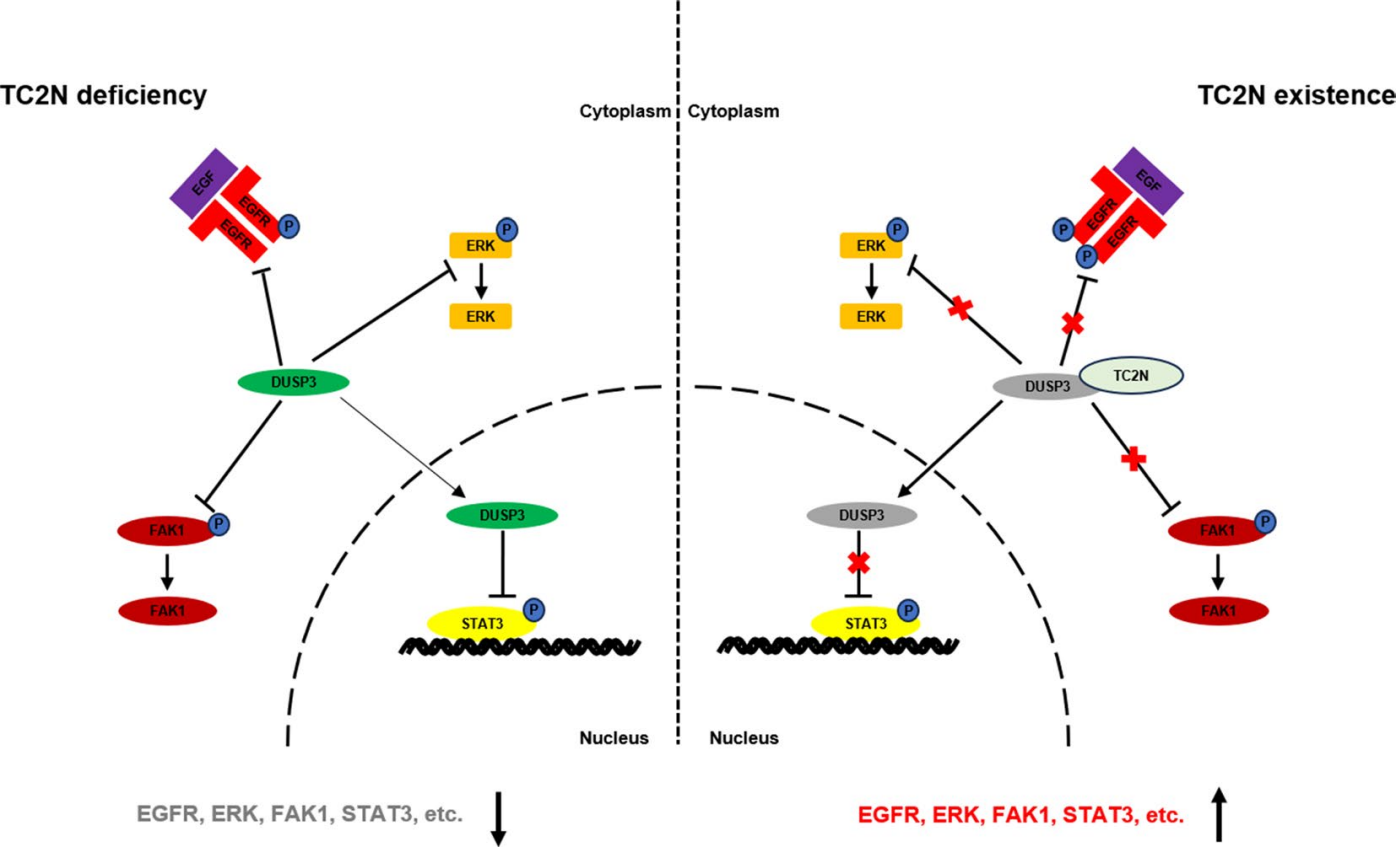
Schematic diagram of TC2N activates EGFR, ERK, STAT3 and FAK1 signaling through blockade of DUSP3 in lung cancer

Taken together, TC2N for the first time was revealed to promote tumorigenesis and augment stem-like properties. Our findings suggested that TC2N can reverse DUSP3-mediated inhibition of EGFR, ERK, STAT3 and FAK1 signaling (Fig. 7). This research provides a novel explanation of how TC2N promotes tumor occurrence and suggests that TC2N is a potential therapeutic strategy for lung cancer treatment.

## Electronic supplementary material

Below is the link to the electronic supplementary material.

Supplementary Material 1

Supplementary Material 2

Supplementary Material 3

Supplementary Material 4

Supplementary Material 5

Supplementary Material 6

Supplementary Material 7

## Data Availability

The datasets used and analyzed during the current study are available from the corresponding author on reasonable request.

## Abbreviations

TC2N: Tandem C2 domains, nuclear
DUSP3: Dual specificity protein phosphatase 3
CSCs: Cancer stem cells
TICs: Tumor-initiating cells
DD: Differentiation degree
TMA: Tissue microarray
H&E: Hematoxylin and Eosin
IHC: Immunohistochemical
TEM: Transmission electron microscope
GO: Gene ontology
GSEA: Gene set enrichment analysis
