# Supplementary material for: TC2N maintains stem cell-like characteristics to accelerate lung carcinogenesis by blockade of dual specificity protein phosphatase 3
Source: Cell Biosci. 2025 Jan 23;15:8. doi: 10.1186/s13578-025-01348-3 (PMC11758731; doi:10.1186/s13578-025-01348-3)

The triplicate of the WB original bands of TC2N in Figure 3B.

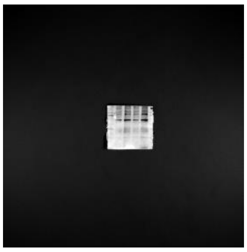

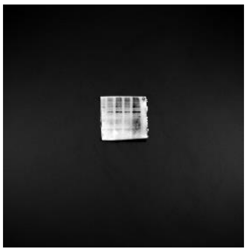

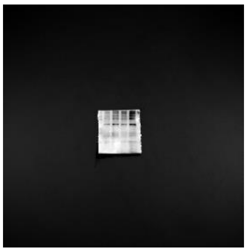

The triplicate of the WB original bands of ACTIN in Figure 3B.

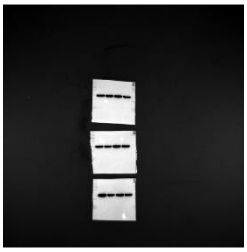

The triplicate of the WB original bands of TC2N in Figure 3C.

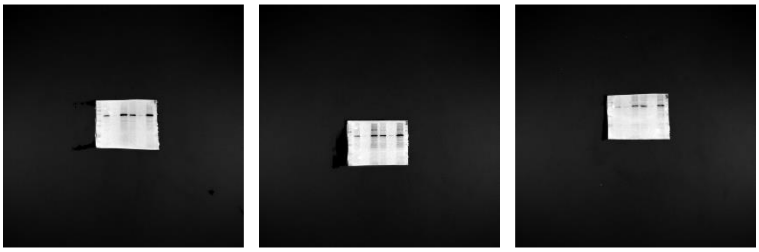

The triplicate of the WB original bands of SOX2 in Figure 3C.

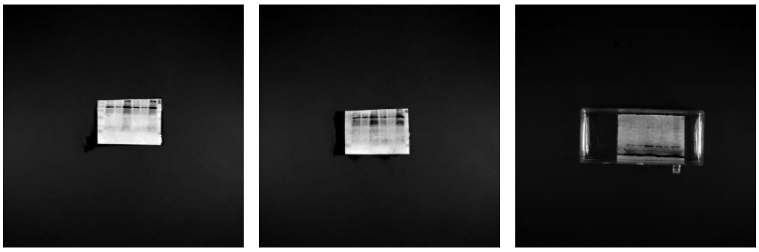

The triplicate of the WB original bands of OCT4 in Figure 3C.

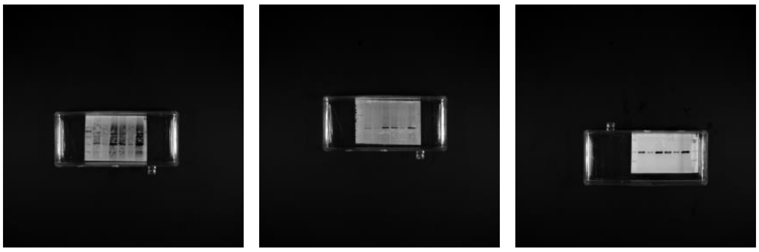

The triplicate of the WB original bands of NANOG in Figure 3C.

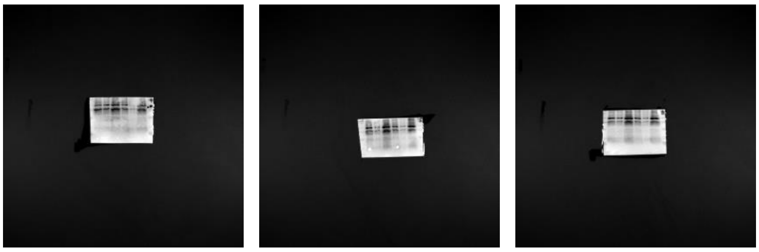

The triplicate of the WB original bands of ACTIN in Figure 3C.

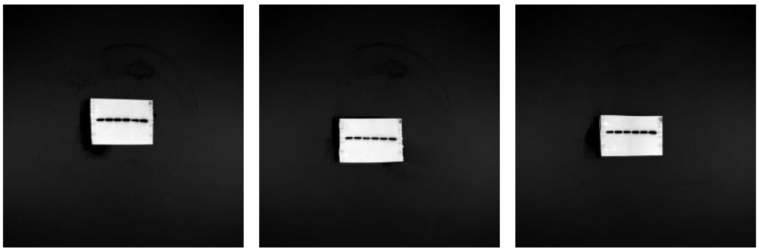

The triplicate of the WB original bands of EGFR in Figure 4E.

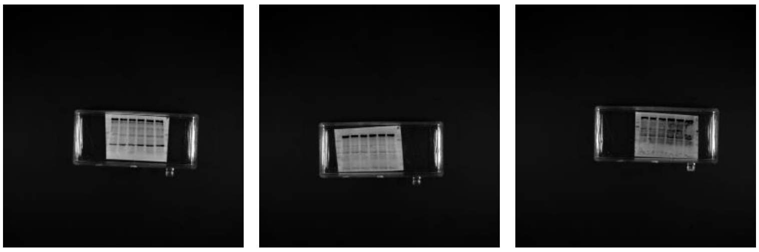

The triplicate of the WB original bands of pEGFR-Y845 in Figure 4E.

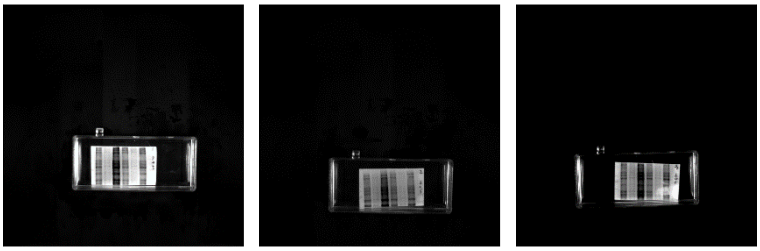

The triplicate of the WB original bands of pEGFR-Y992 in Figure 4E.

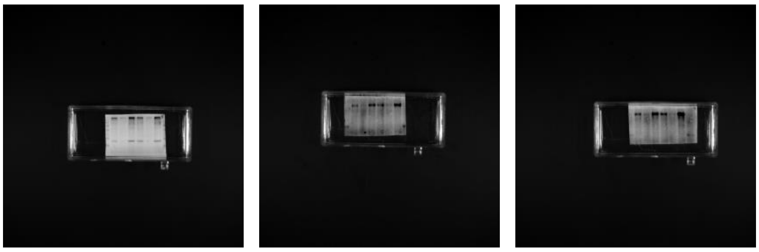

The triplicate of the WB original bands of STAT3 in Figure 4E.

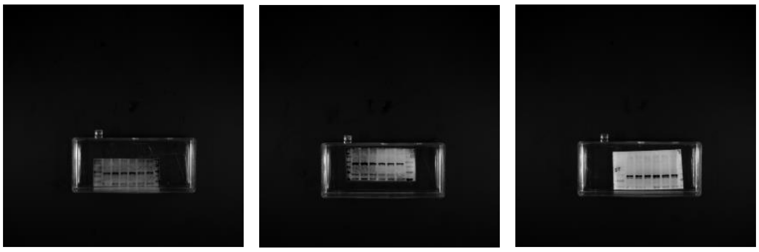

The triplicate of the WB original bands of pSTAT3-S727 in Figure 4E.

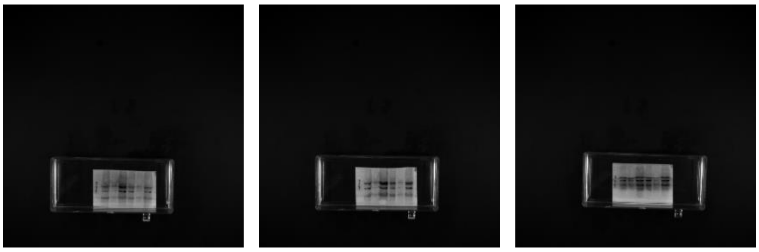

The triplicate of the WB original bands of pSTAT3-Y705 in Figure 4E.

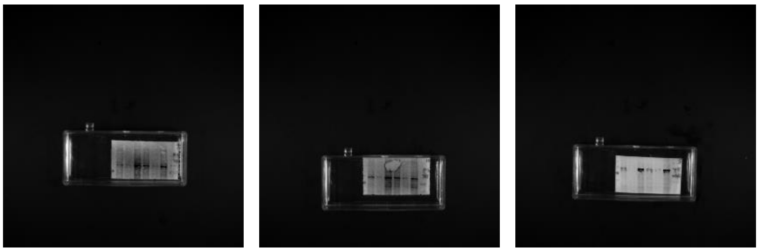

The triplicate of the WB original bands of ERK1/2 in Figure 4E.

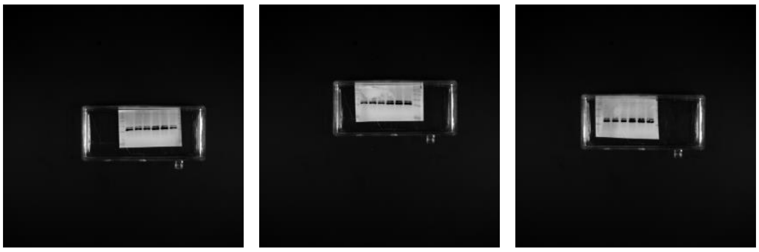

The triplicate of the WB original bands of pERK1/2 in Figure 4E.

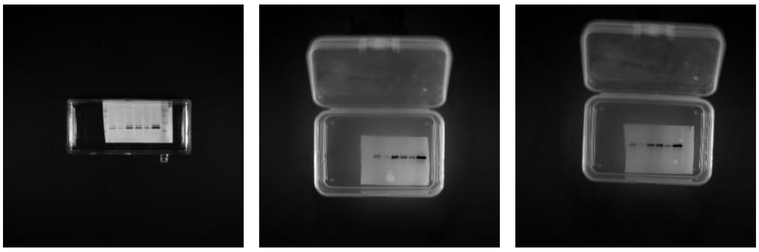

The triplicate of the WB original bands of FAK1 in Figure 4E.

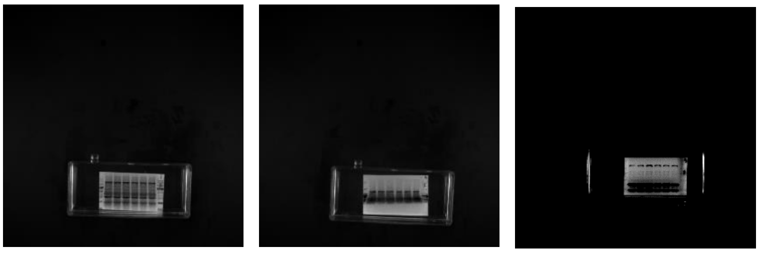

The triplicate of the WB original bands of pFAK1-Y576 in Figure 4E.

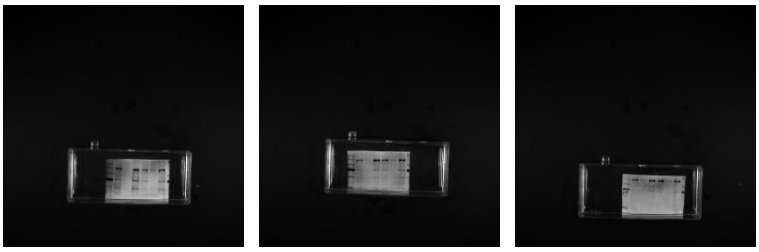

The triplicate of the WB original bands of ACTIN in Figure 4E.

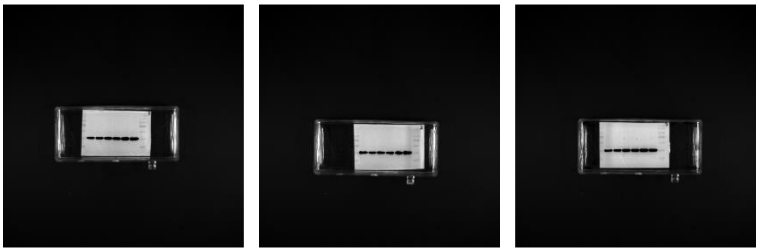

The triplicate of the WB original bands of TC2N in Figure 4F.

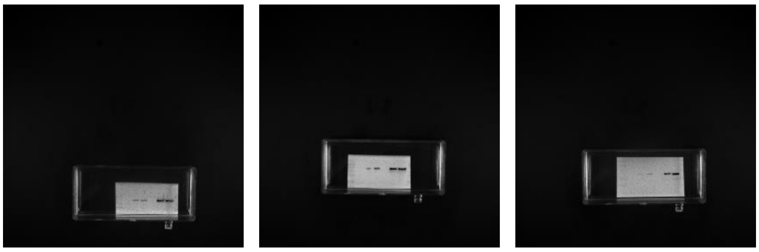

The triplicate of the WB original bands of EGFR in Figure 4F.

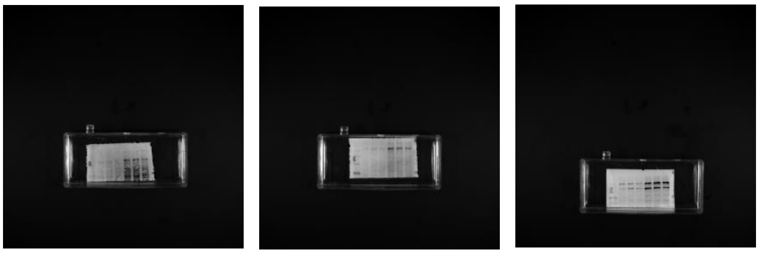

The triplicate of the WB original bands of pEGFR-Y845 in Figure 4F.

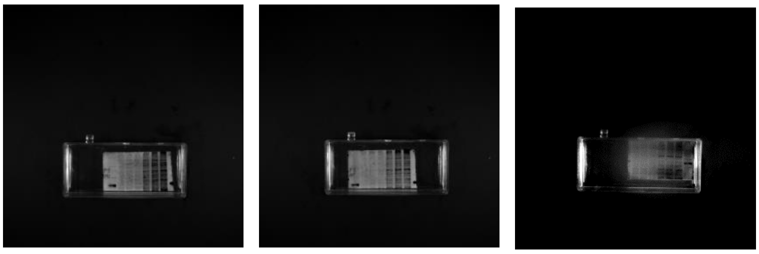

The triplicate of the WB original bands of pEGFR-Y992 in Figure 4F.

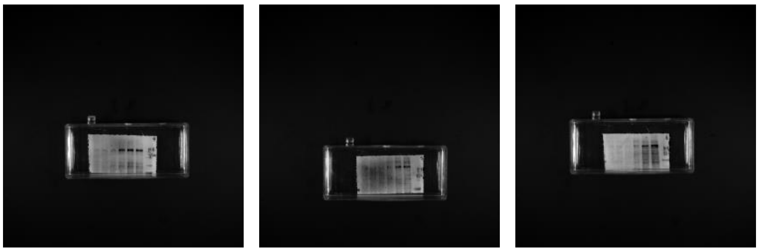

The triplicate of the WB original bands of STAT3 in Figure 4F.

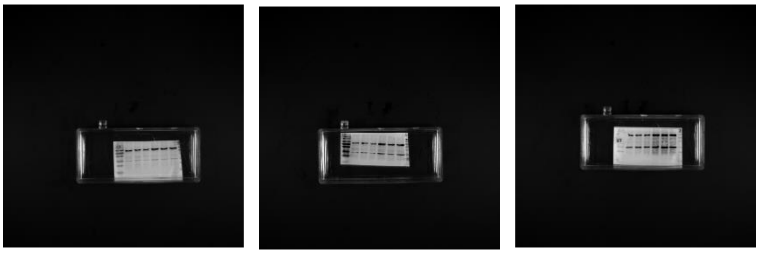

The triplicate of the WB original bands of pSTAT3-S727 in Figure 4F.

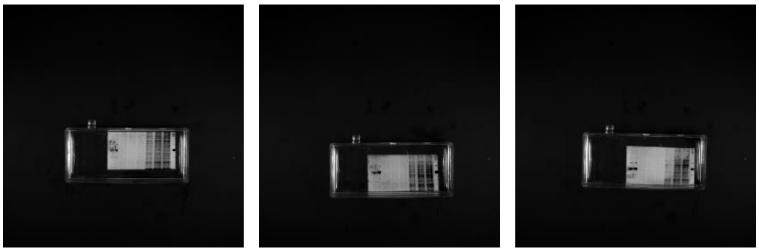

The triplicate of the WB original bands of pSTAT3-Y705 in Figure 4F.

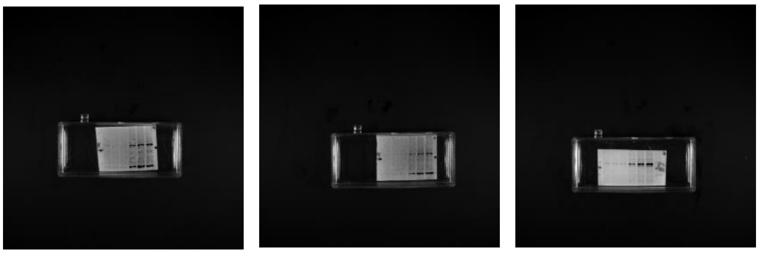

The triplicate of the WB original bands of ERK1/2 in Figure 4F.

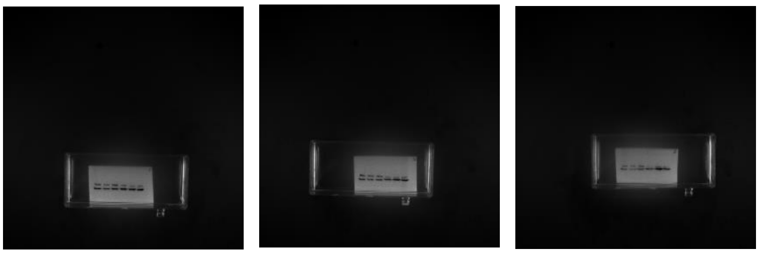

The triplicate of the WB original bands of pERK1/2 in Figure 4F.

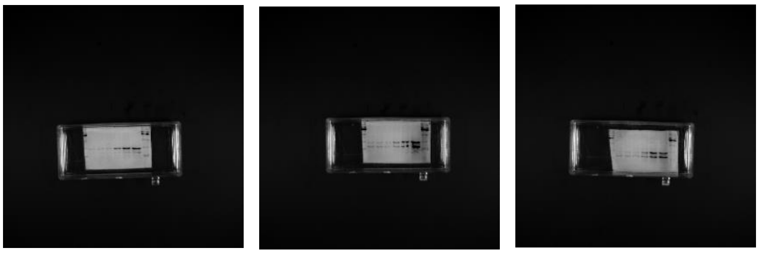

The triplicate of the WB original bands of FAK1 in Figure 4F.

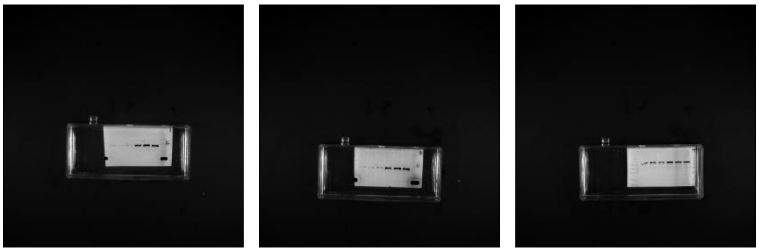

The triplicate of the WB original bands of pFAK1 in Figure 4F.

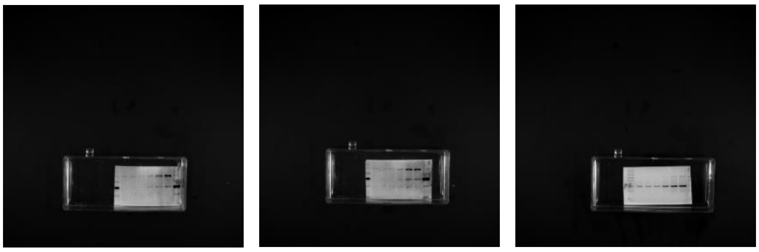

The triplicate of the WB original bands of ACTIN in Figure 4F.

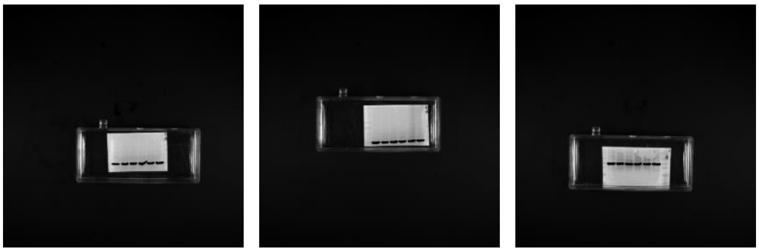

The triplicate of the WB original bands of TC2N in Figure 5B.

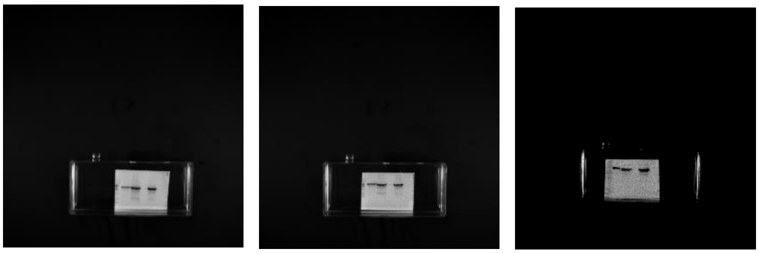

The triplicate of the WB original bands of DUSP3 in Figure 5B.

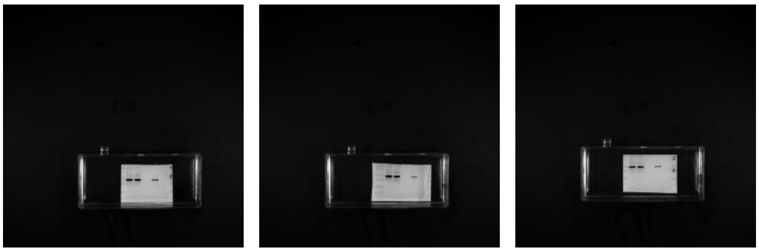

The triplicate of the WB original bands of EGFR in Figure 5B.

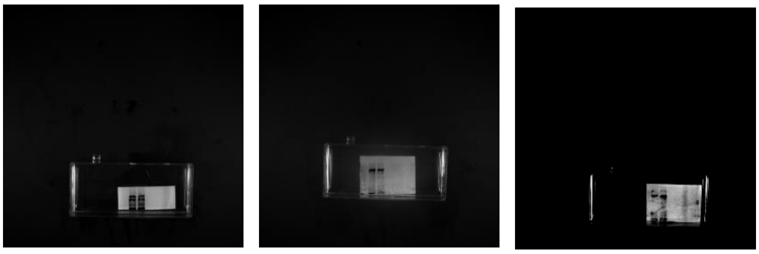

The triplicate of the WB original bands of STAT3 in Figure 5B.

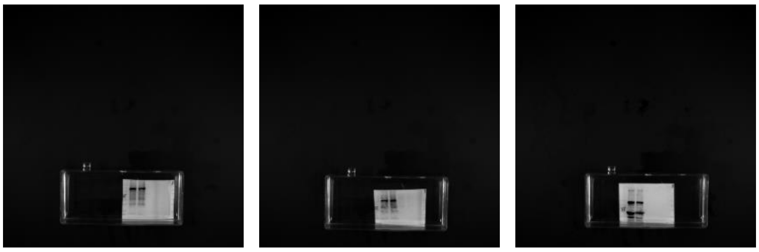

The triplicate of the WB original bands of ERK1/2 in Figure 5B.

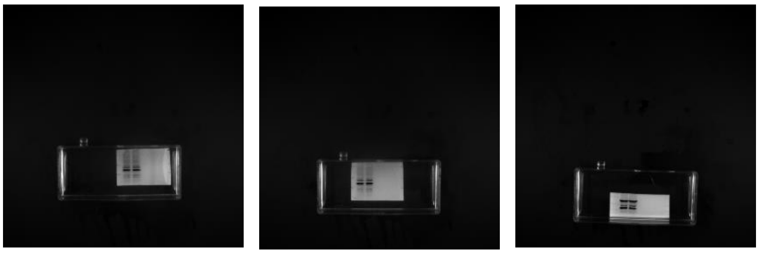

The triplicate of the WB original bands of FAK1 in Figure 5B.

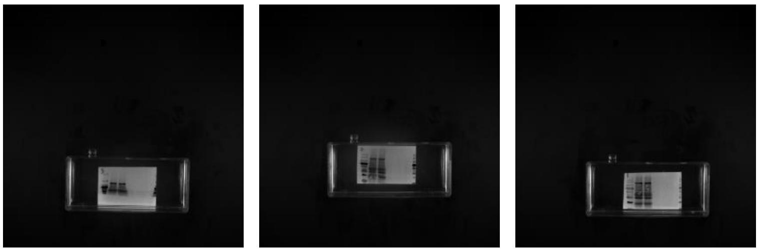

The triplicate of the WB original bands of TC2N in Figure 5C.

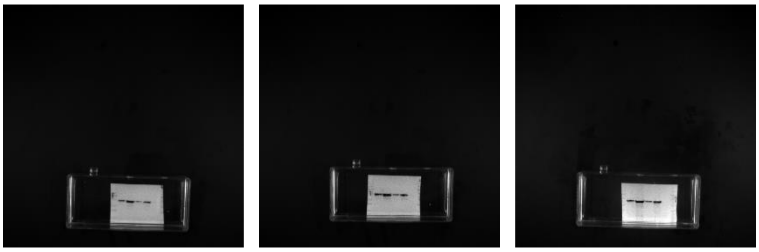

The triplicate of the WB original bands of DUSP3 in Figure 5C.

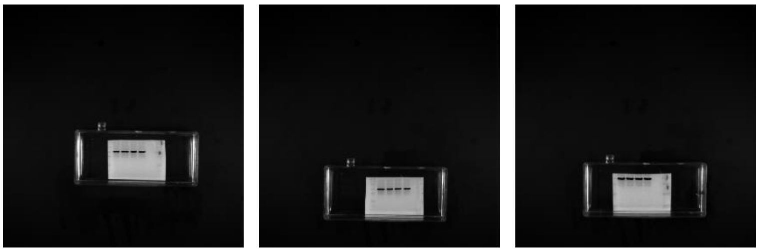

The triplicate of the WB original bands of EGFR in Figure 5C.

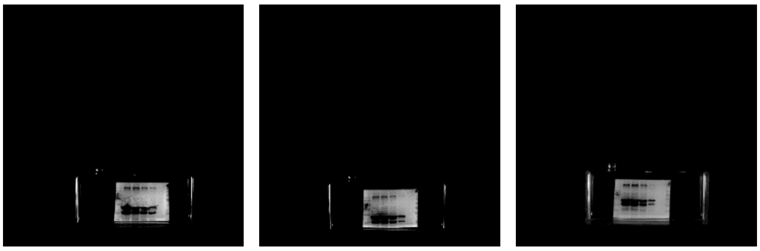

The triplicate of the WB original bands of STAT3 in Figure 5C.

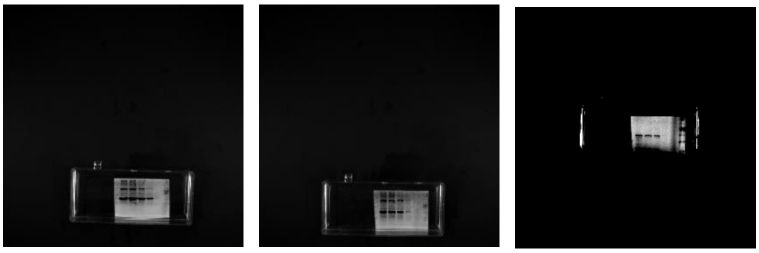

The triplicate of the WB original bands of ERK1/2 in Figure 5C.

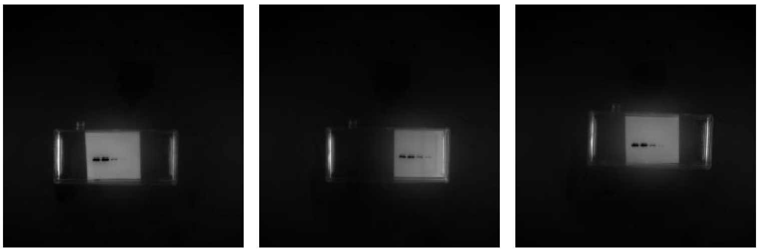

The triplicate of the WB original bands of FAK1 in Figure 5C.

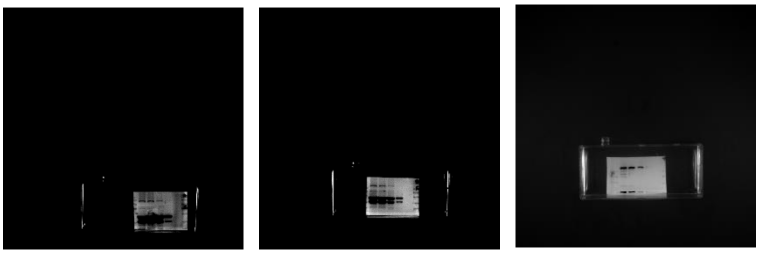

The triplicate of the WB original bands of DUSP3 in Figure 5D.

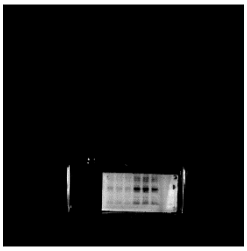
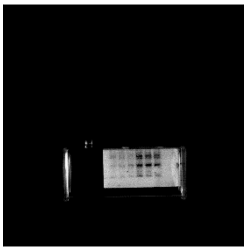
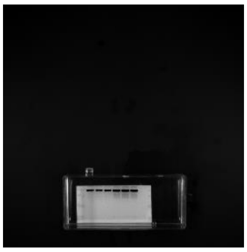

The triplicate of the WB original bands of EGFR in Figure 5D.

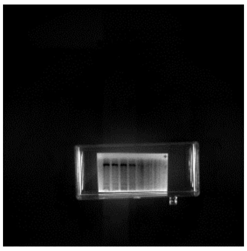
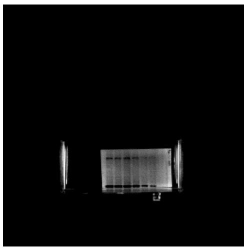
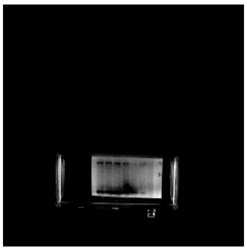

The triplicate of the WB original bands of STAT3 in Figure 5D.

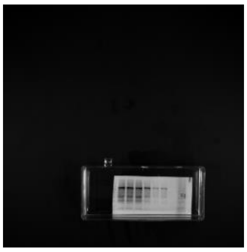
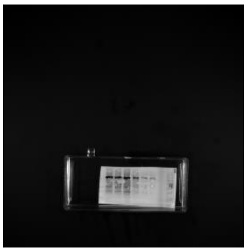
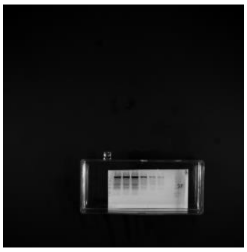

The triplicate of the WB original bands of ERK1/2 in Figure 5D.

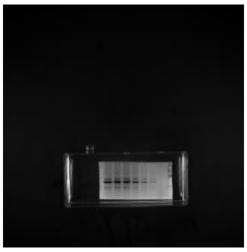
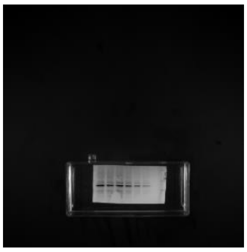
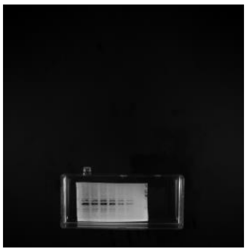

The triplicate of the WB original bands of FAK1 in Figure 5D.

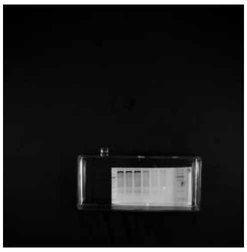
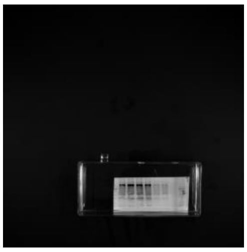
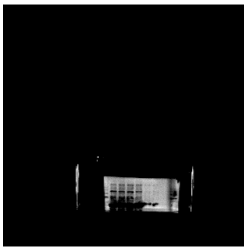

The triplicate of the WB original bands of DUSP3 in Figure 5E.

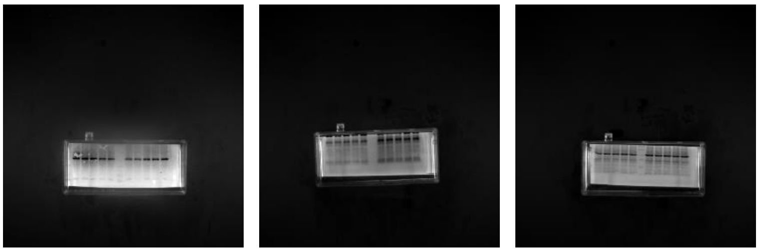

The triplicate of the WB original bands of Flag in Figure 5E.

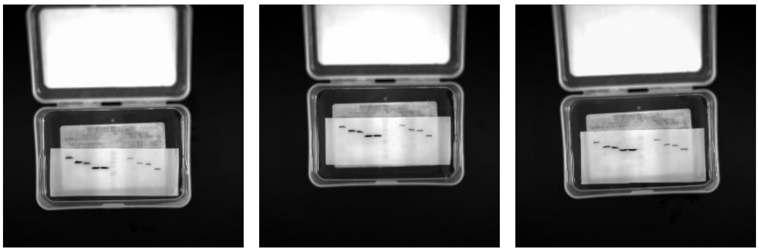

The triplicate of the WB original bands of DUSP3 in Figure 6D.

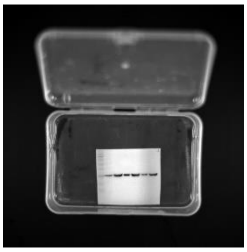

The triplicate of the WB original bands of ACTIN in Figure 6D.

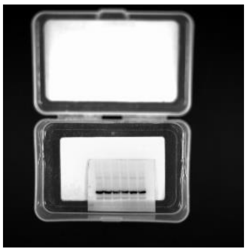

The triplicate of the WB original bands of TC2N in Figure 6G.

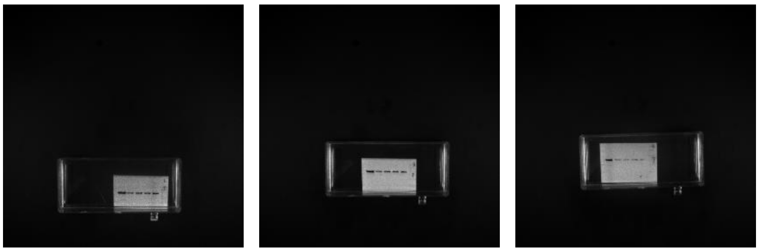

The triplicate of the WB original bands of DUSP3 in Figure 6G.

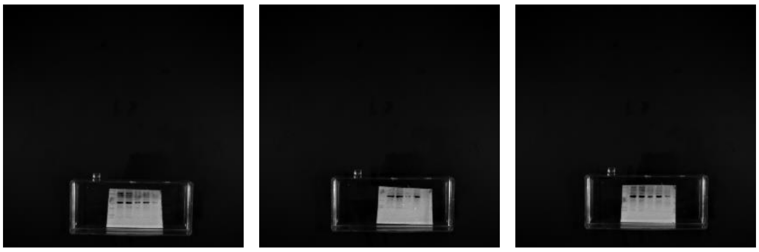

The triplicate of the WB original bands of EGFR in Figure 6G.

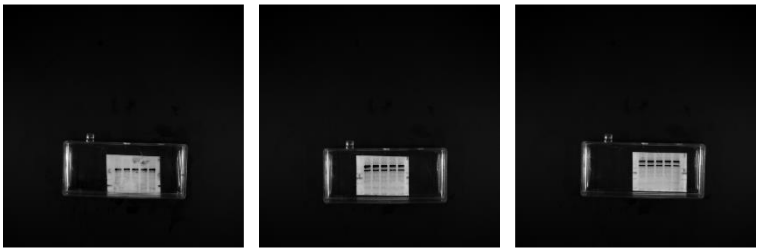

The triplicate of the WB original bands of pEGFR-Y845 in Figure 6G.

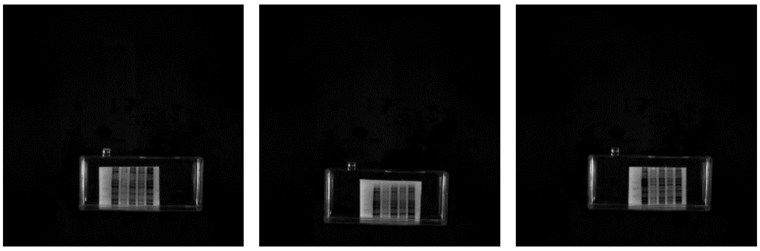

The triplicate of the WB original bands of pEGFR-Y992 in Figure 6G.

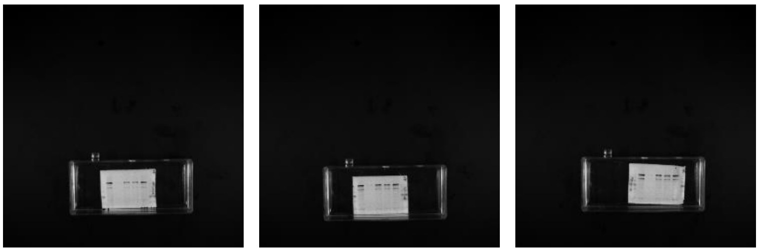

The triplicate of the WB original bands of STAT3 in Figure 6G.

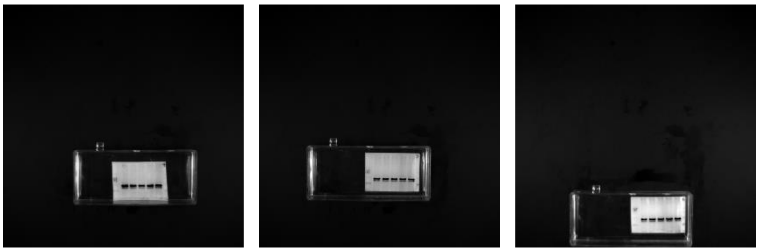

The triplicate of the WB original bands of pSTAT3-S727 in Figure 6G.

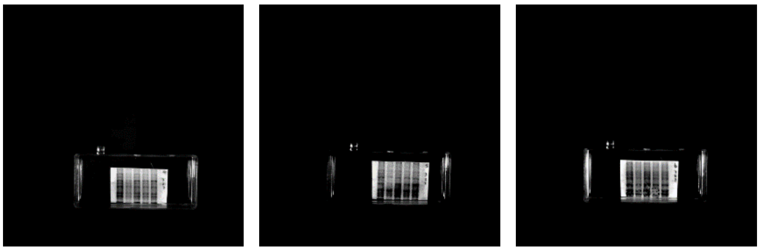

The triplicate of the WB original bands of pSTAT3-Y705 in Figure 6G.

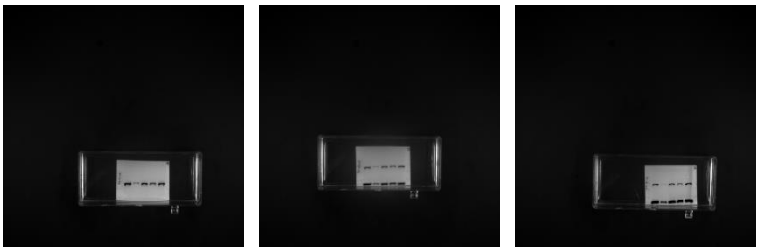

The triplicate of the WB original bands of ERK1/2 in Figure 6G.

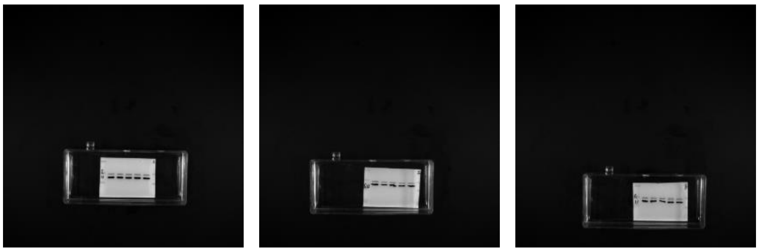

The triplicate of the WB original bands of pERK1/2 in Figure 6G.

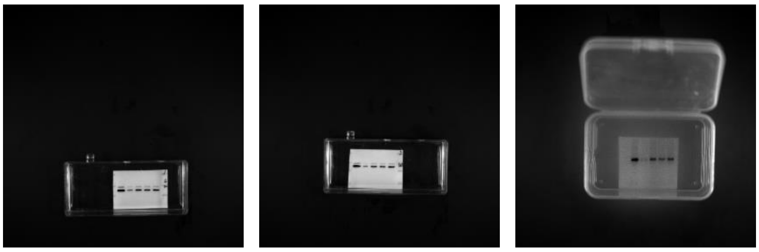

The triplicate of the WB original bands of FAK1 in Figure 6G.

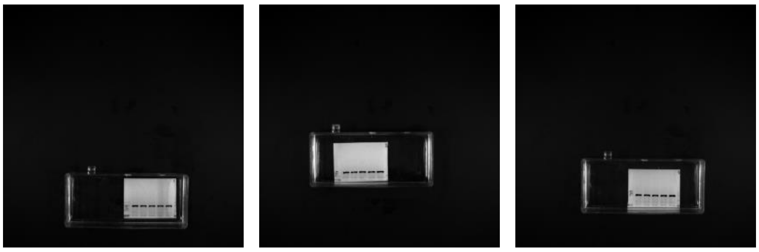

The triplicate of the WB original bands of pFAK1-Y576 in Figure 6G.

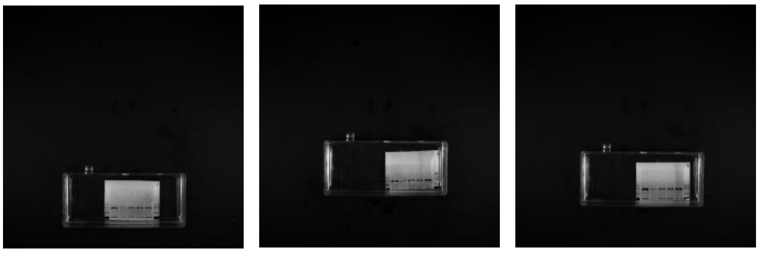

The triplicate of the WB original bands of SOX2 in Figure 6G.

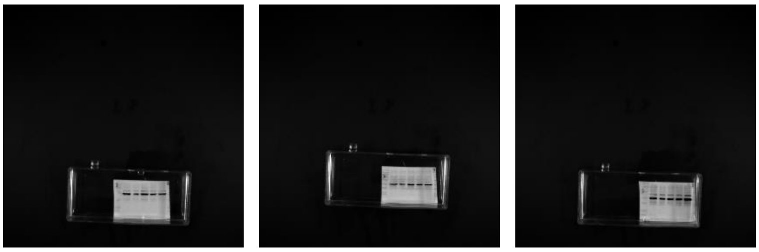

The triplicate of the WB original bands of OCT4 in Figure 6G.

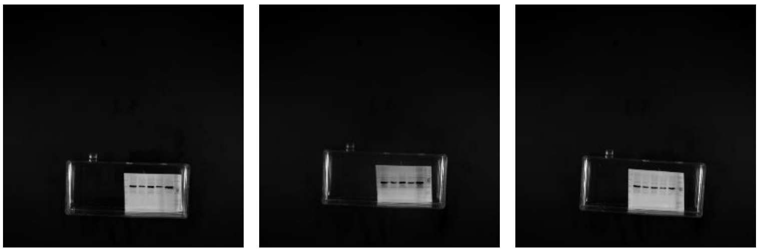

The triplicate of the WB original bands of NANOG in Figure 6G.

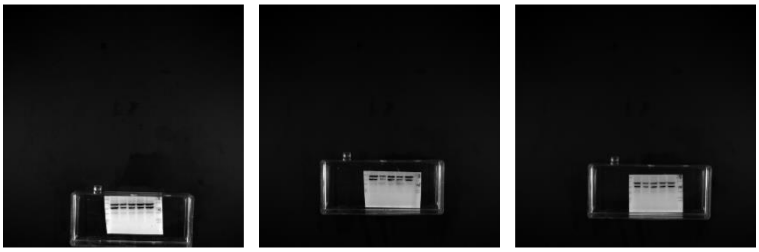

The triplicate of the WB original bands of ACTIN in Figure 6G.

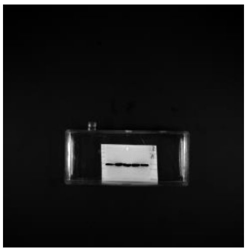

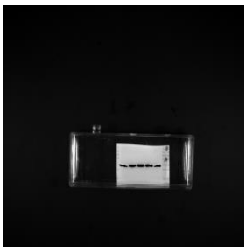

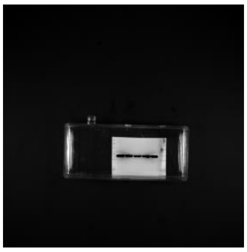

Supplement: Supplementary file 6 — Supplementary Material 6 [file 13578_2025_1348_MOESM6_ESM.pdf]
