# Supplementary material for: TC2N maintains stem cell-like characteristics to accelerate lung carcinogenesis by blockade of dual specificity protein phosphatase 3
Source: Cell Biosci. 2025 Jan 23;15:8. doi: 10.1186/s13578-025-01348-3 (PMC11758731; doi:10.1186/s13578-025-01348-3)

The original HE images in Figure 1E.

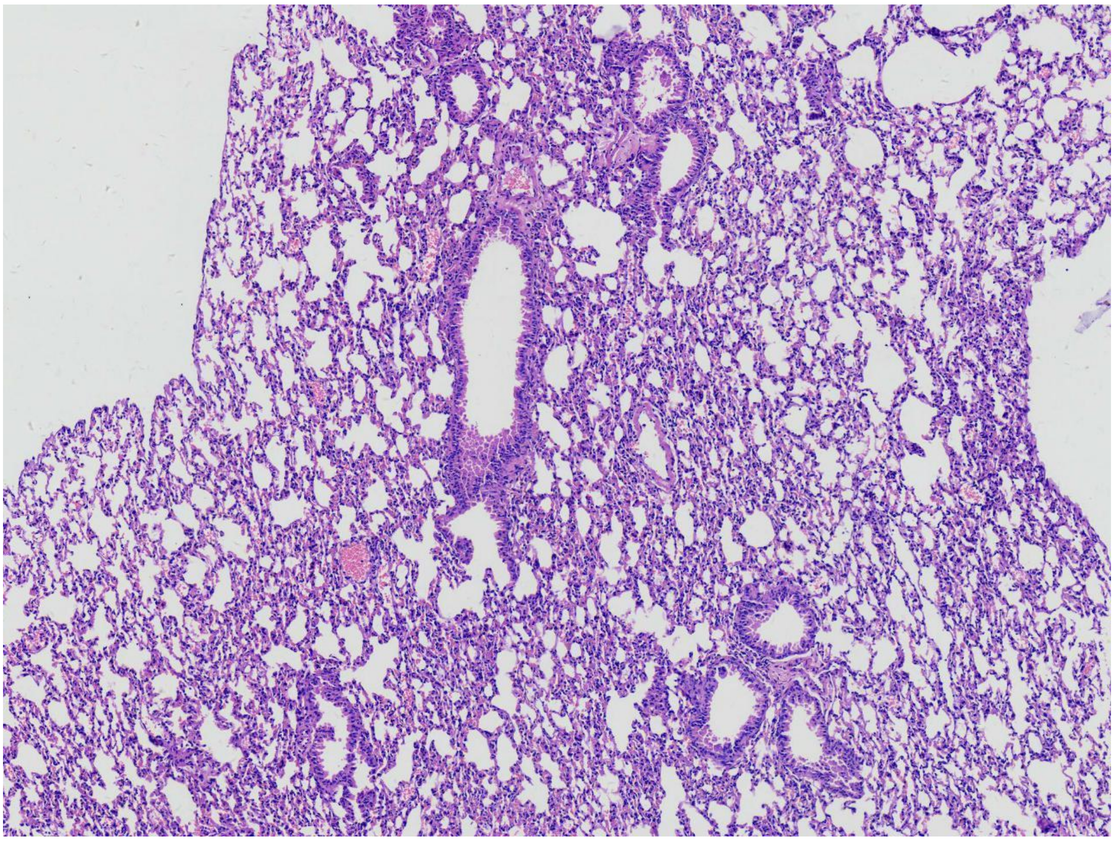

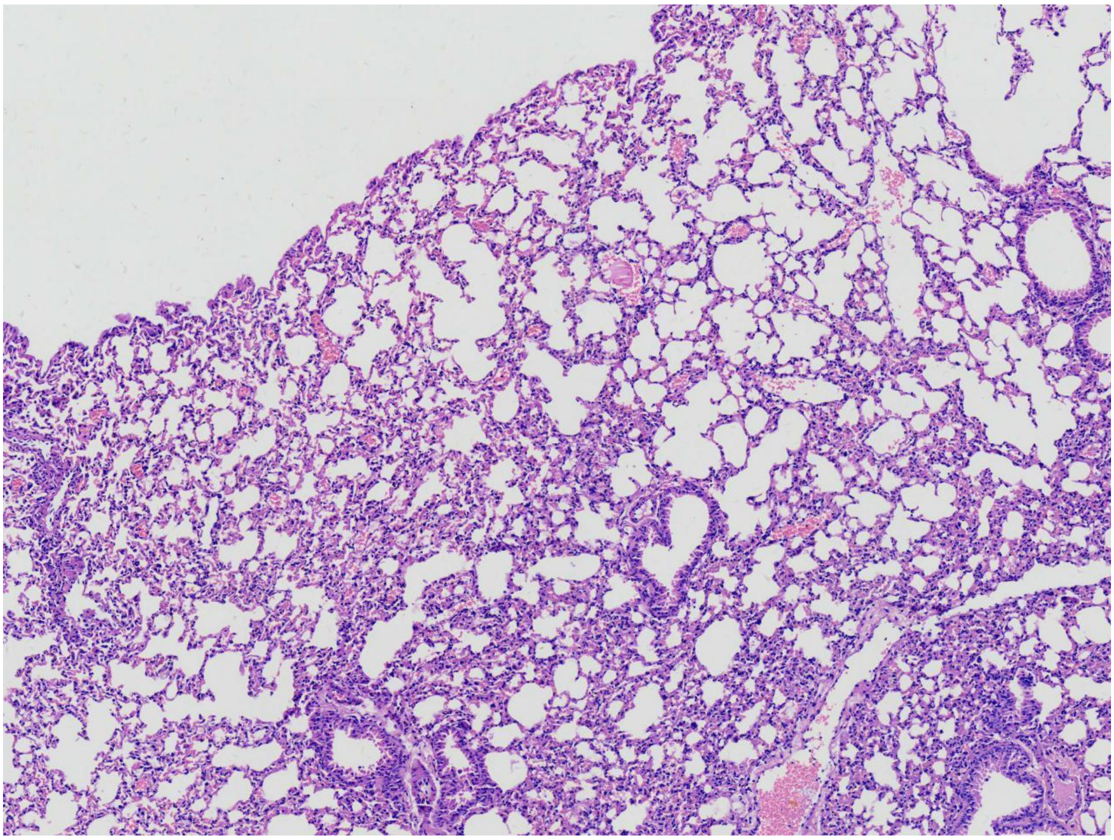

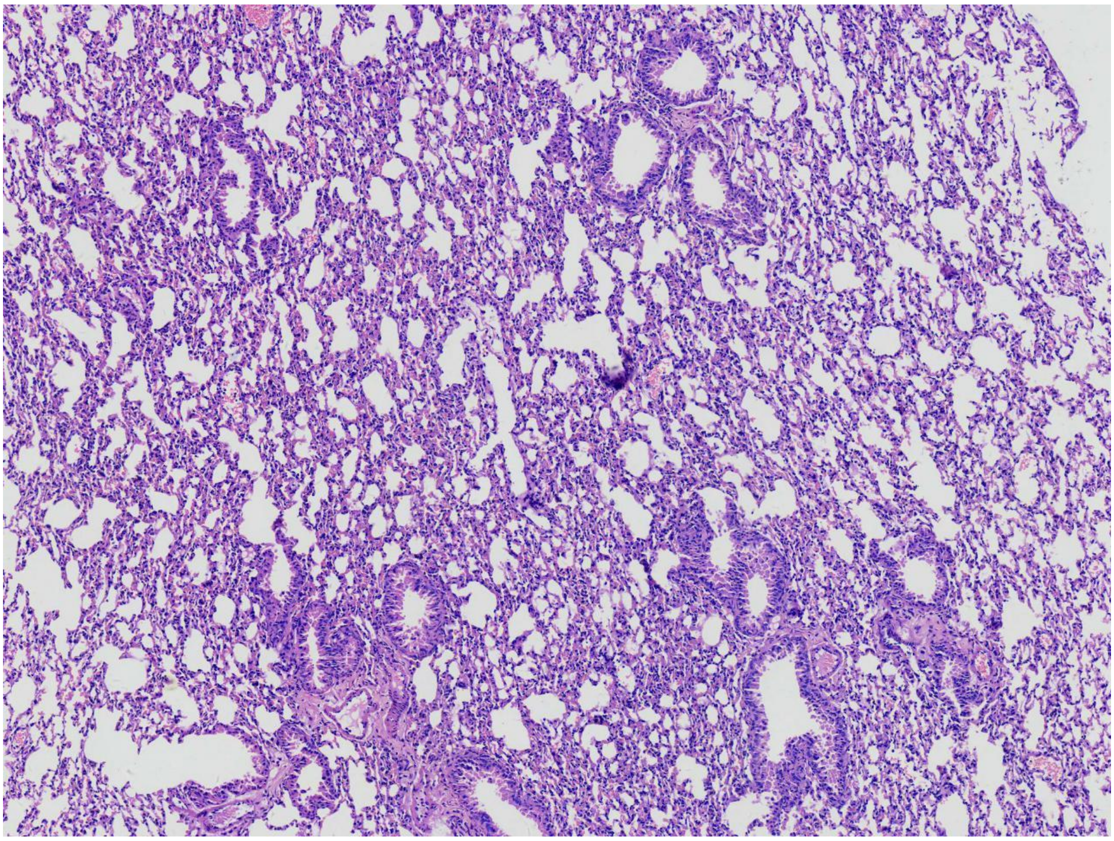

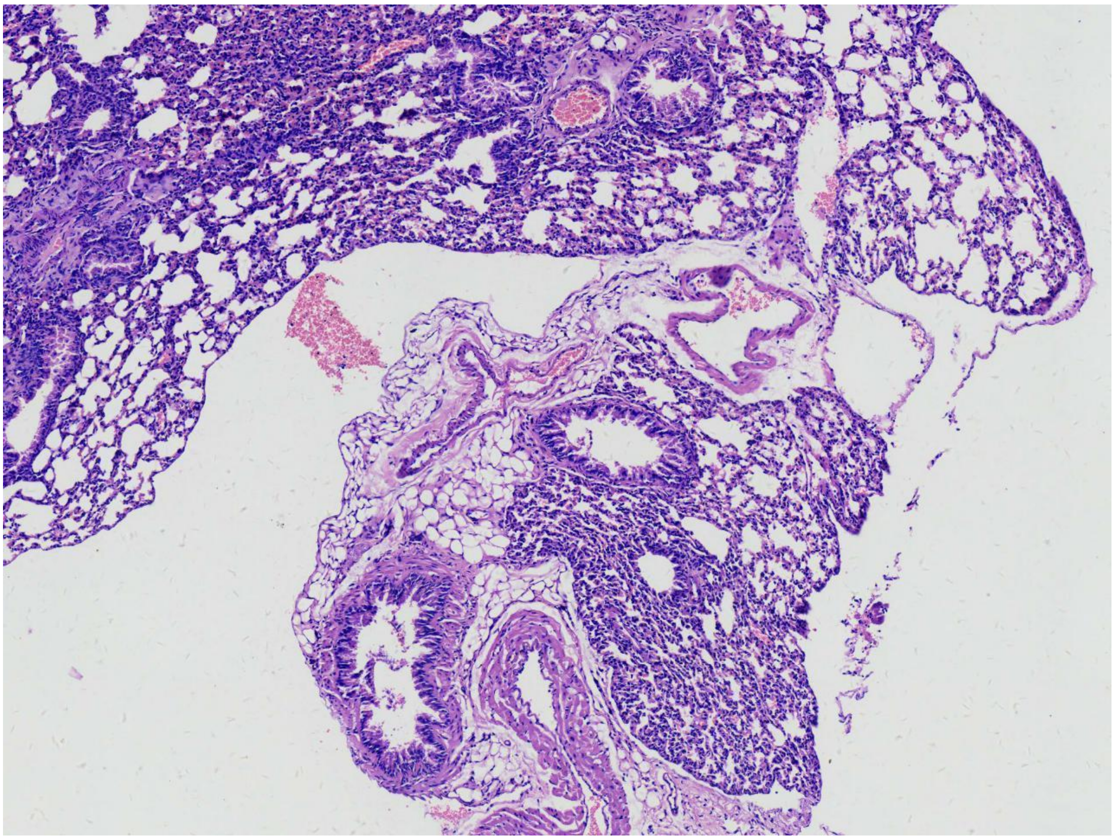

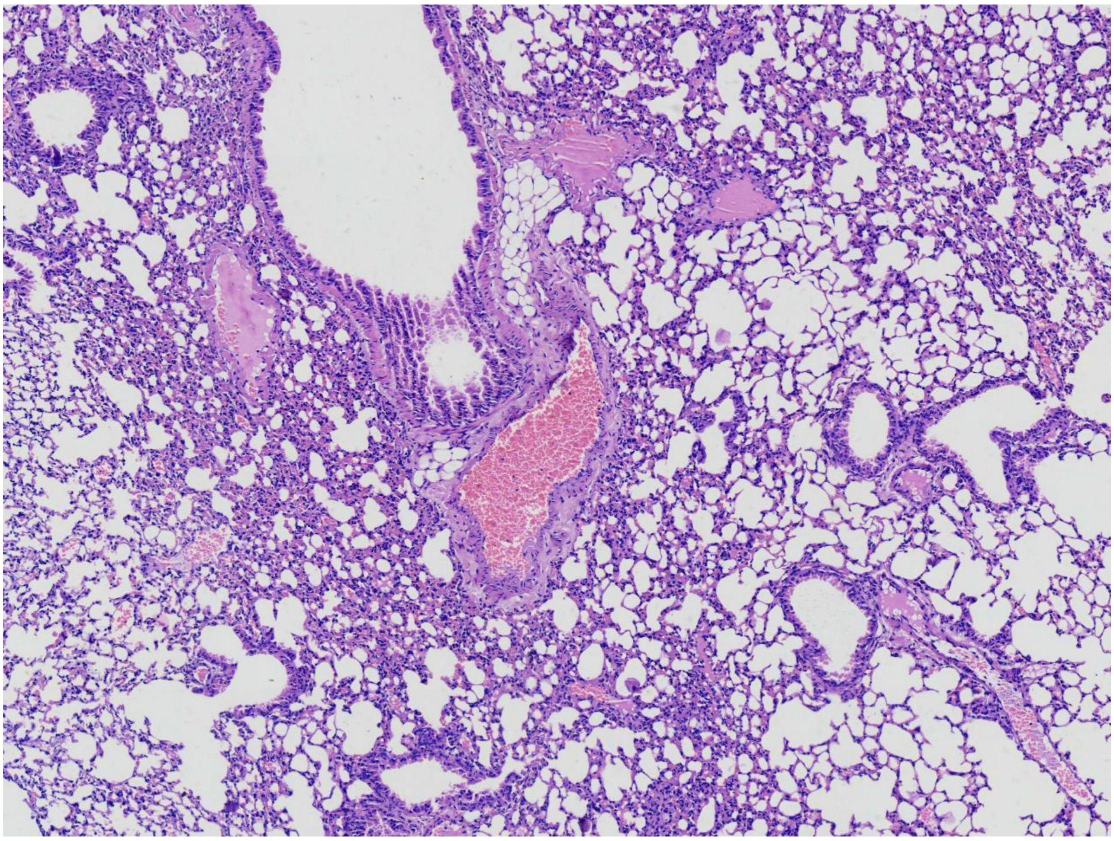

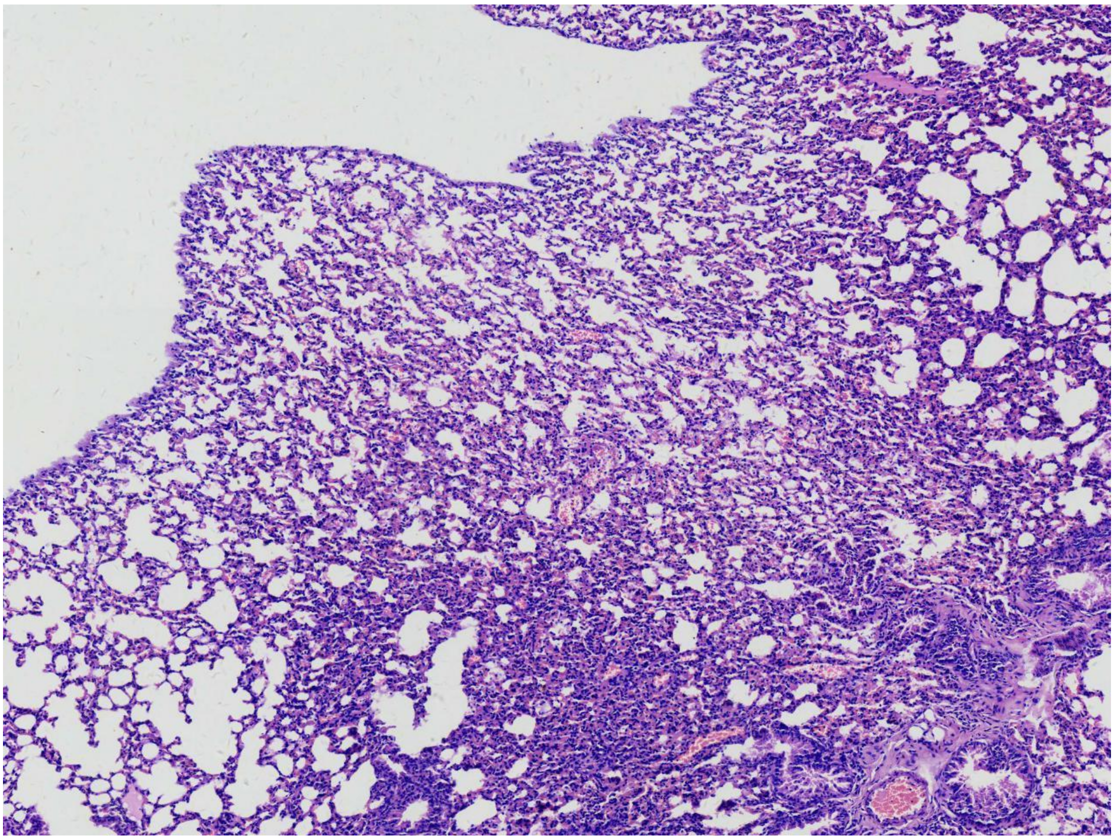

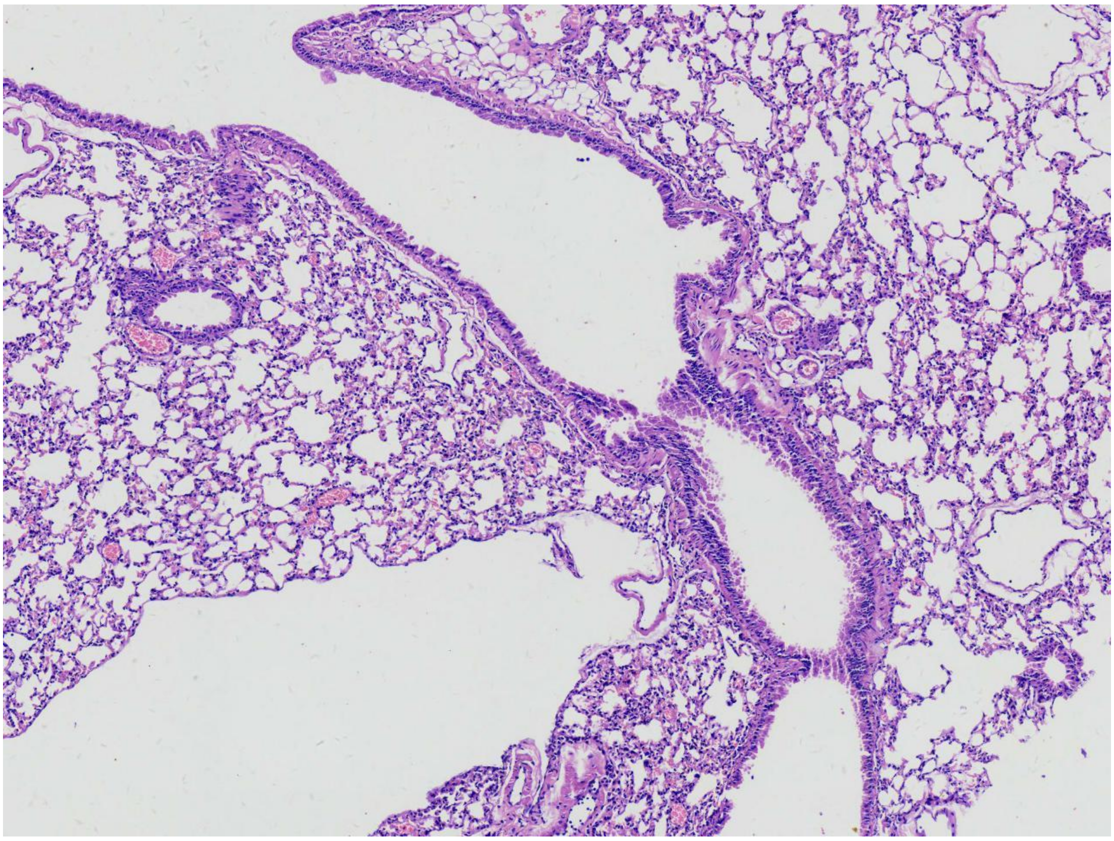

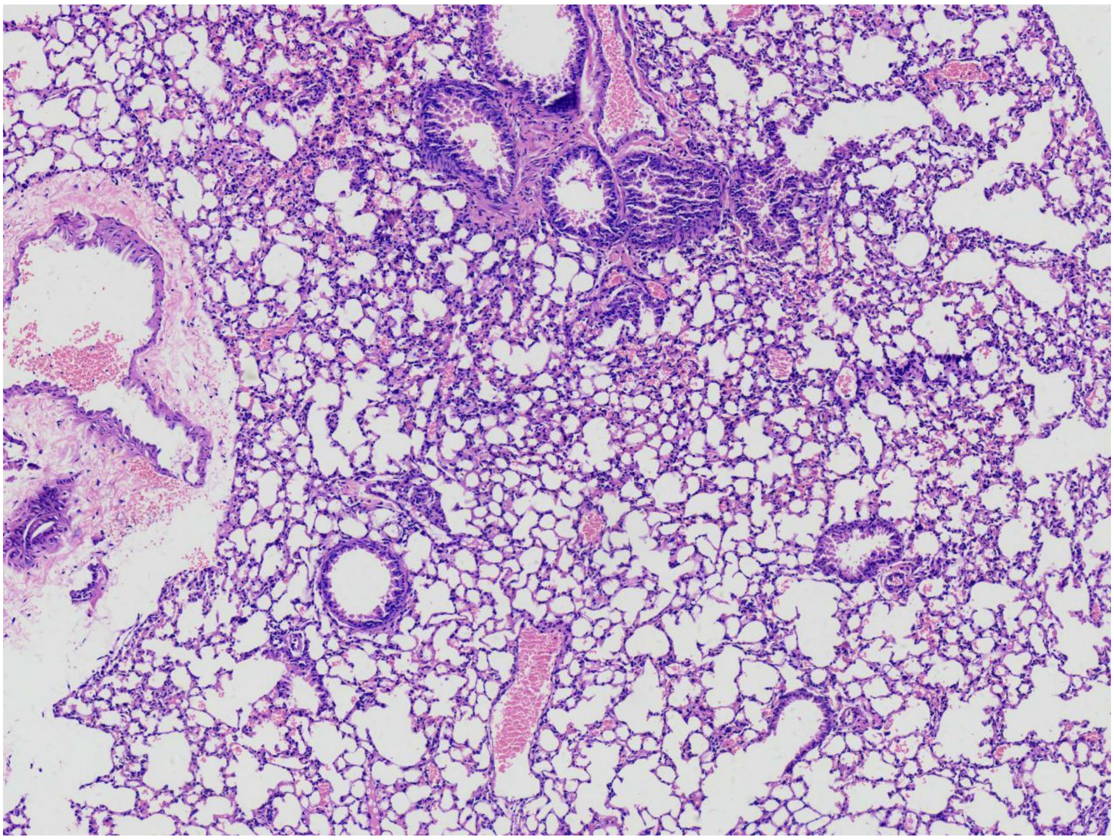

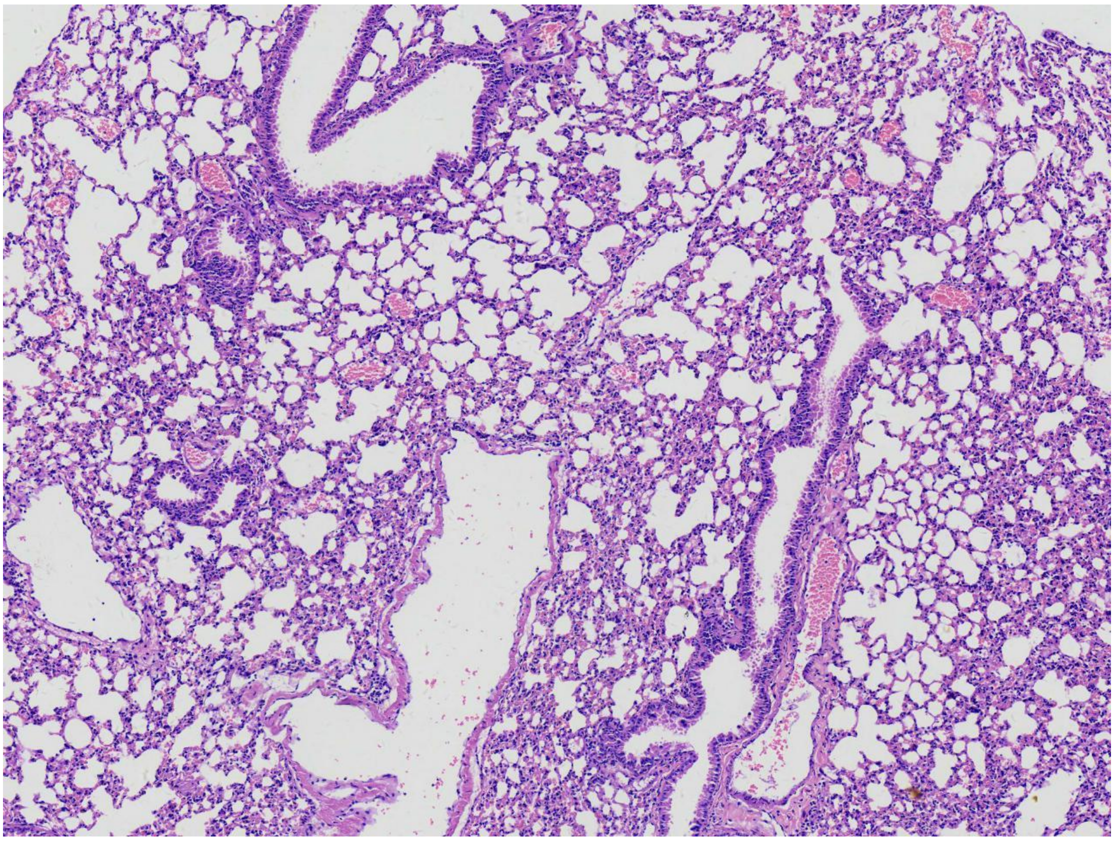

The original IHC images in Figure 1F.

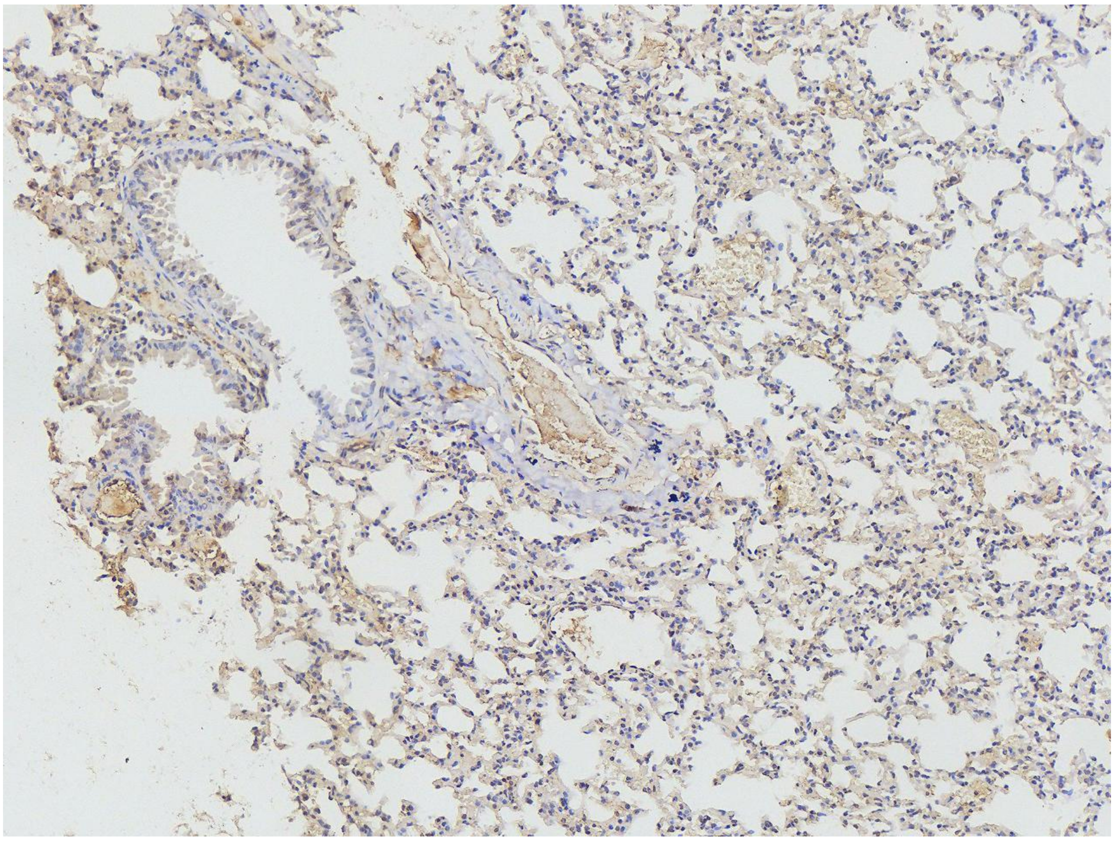

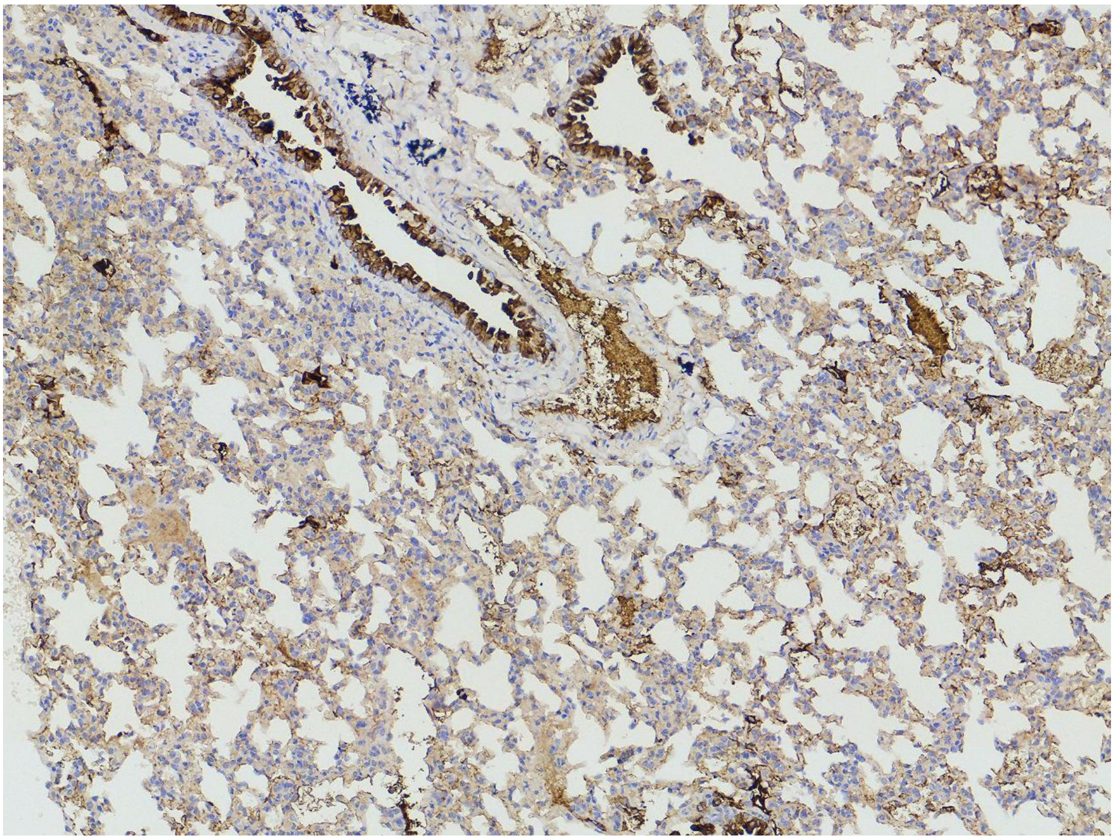

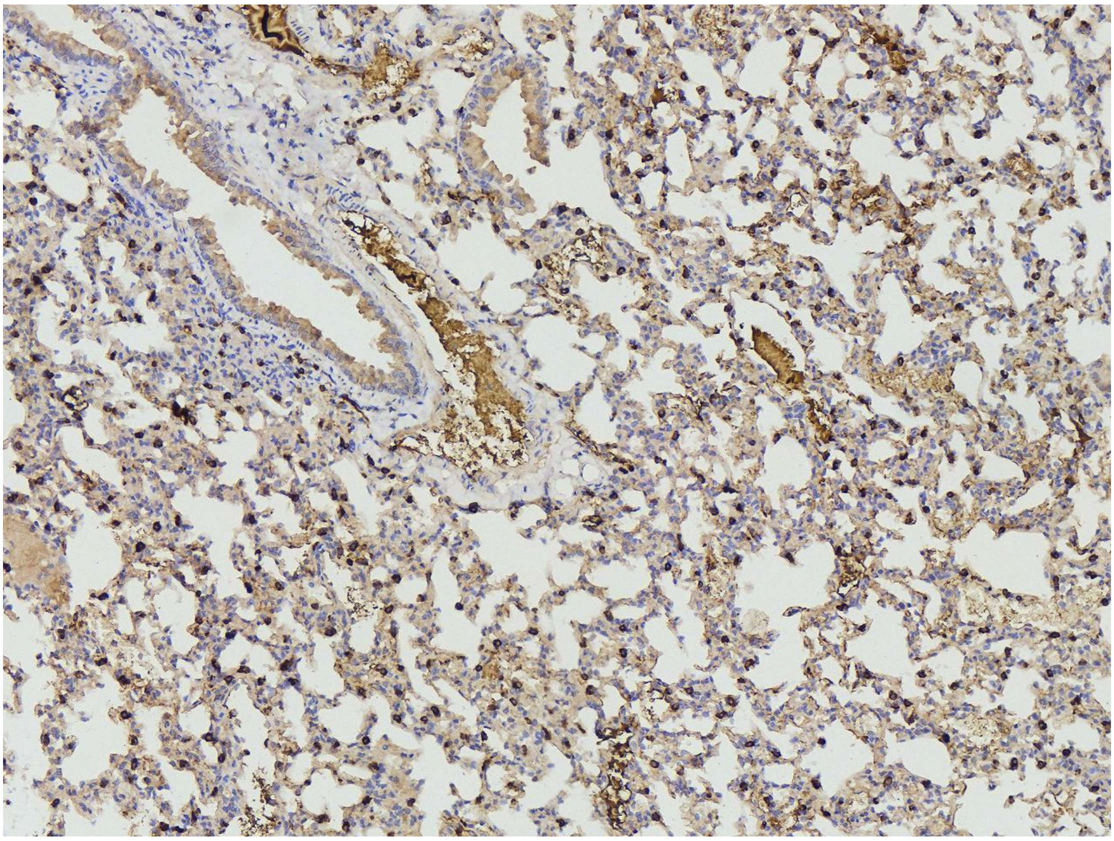

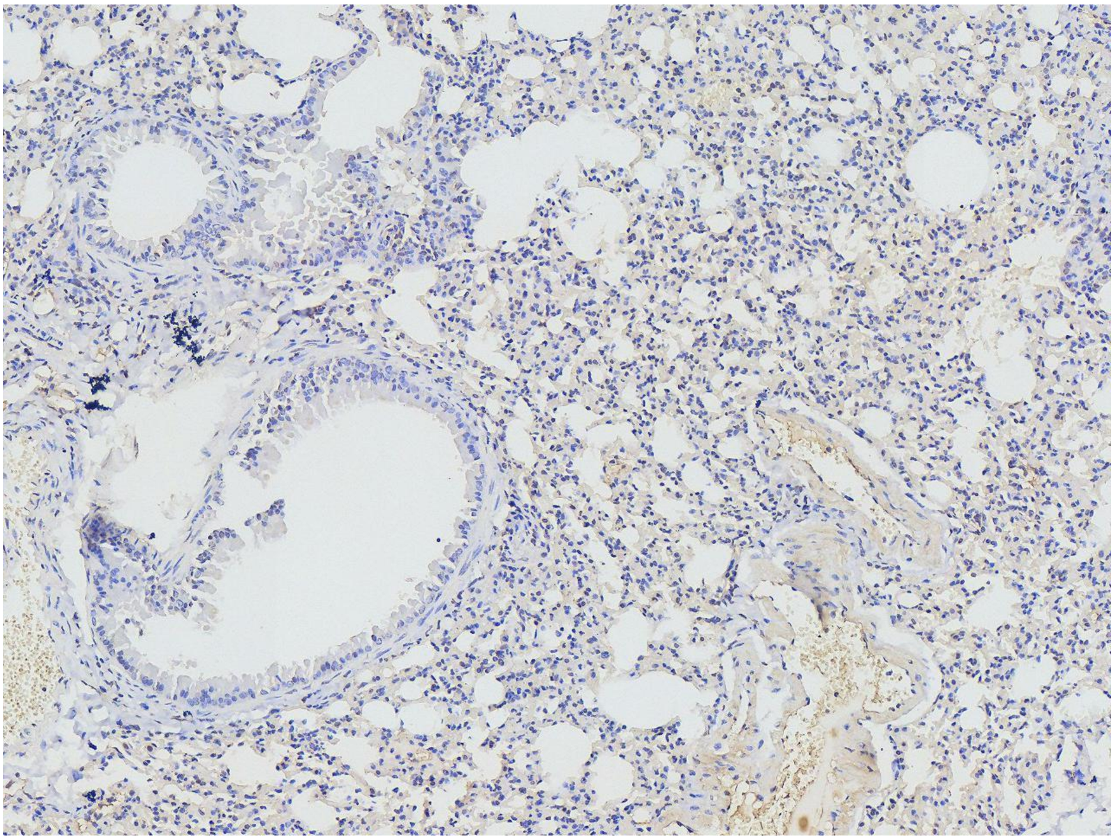

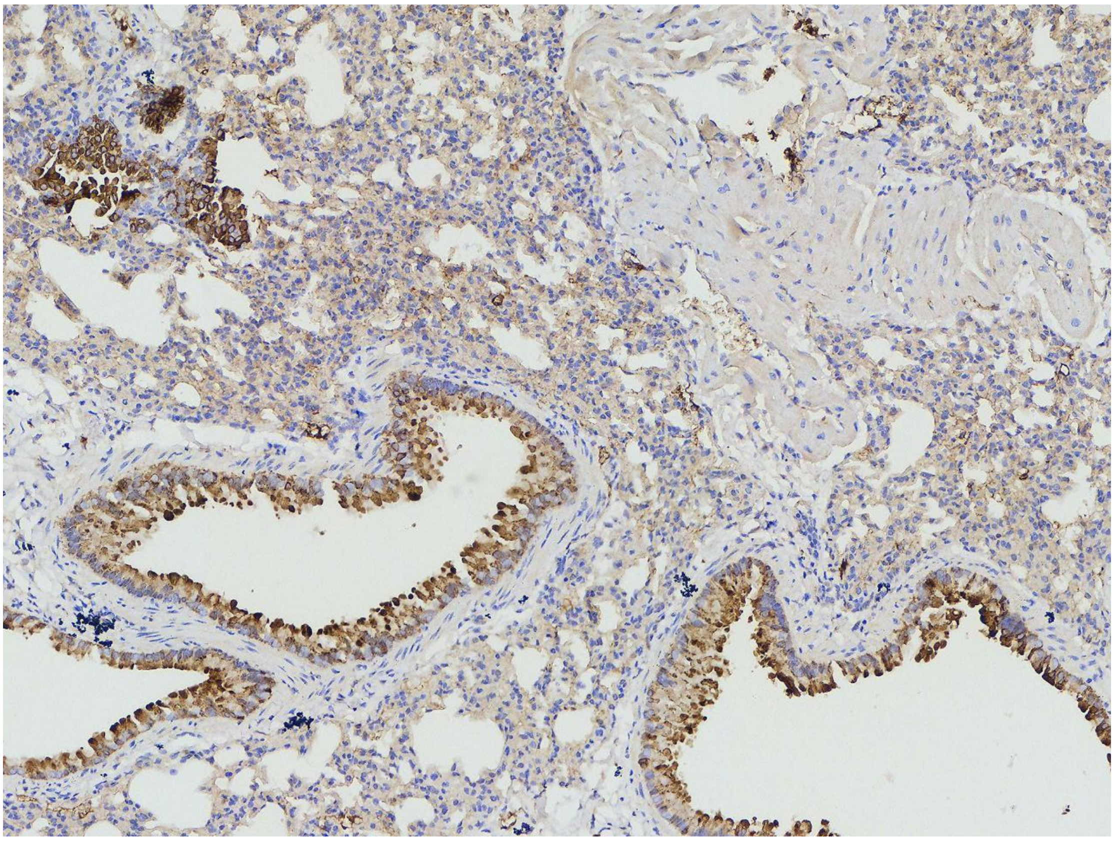

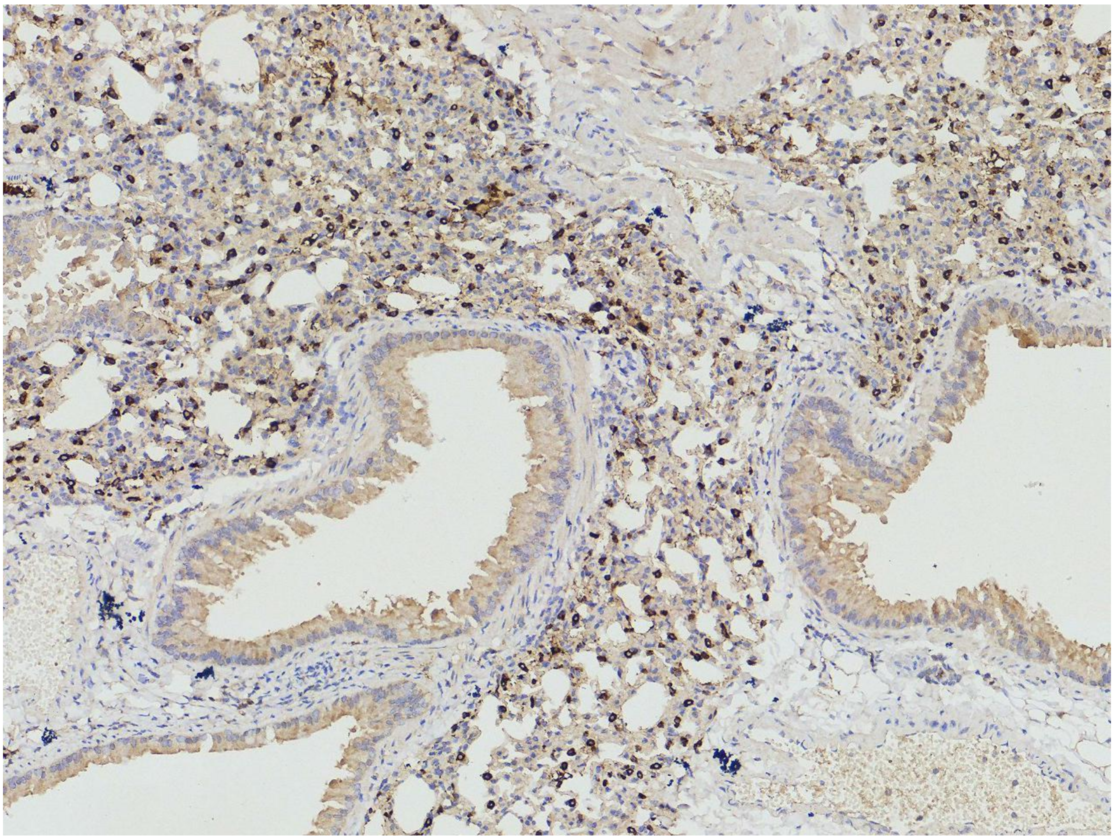

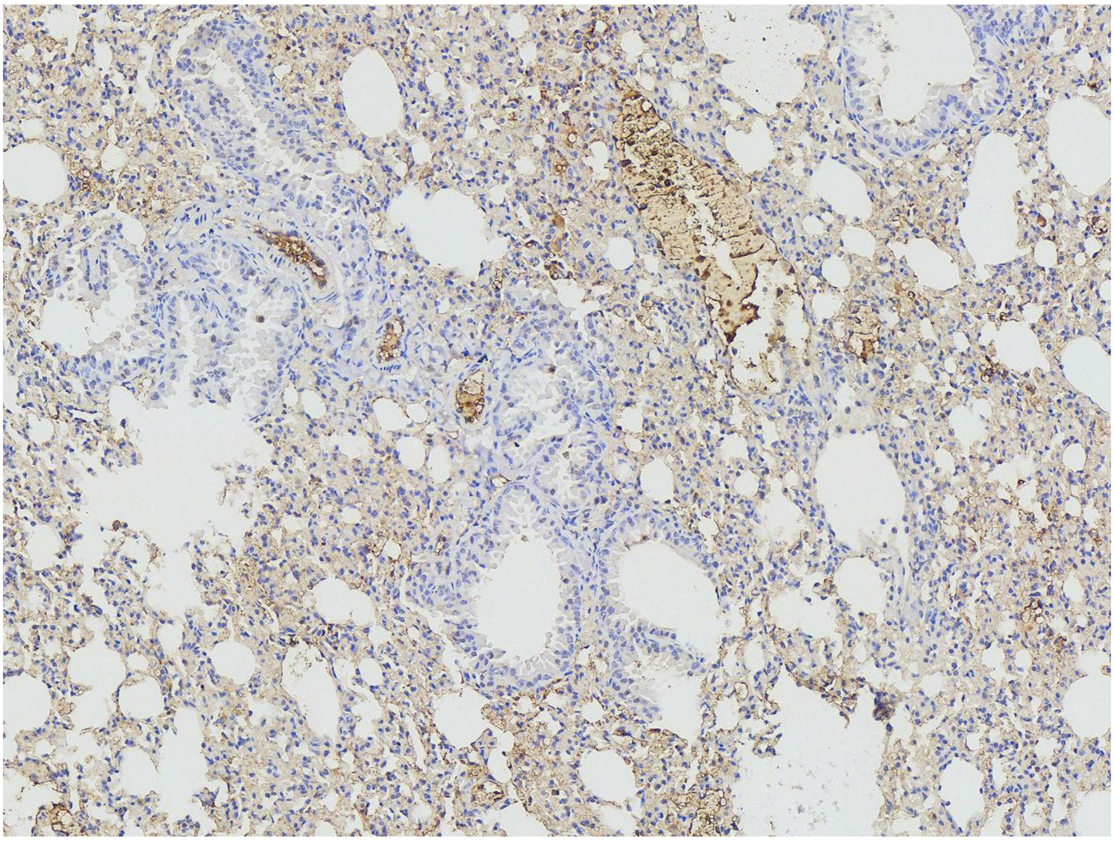

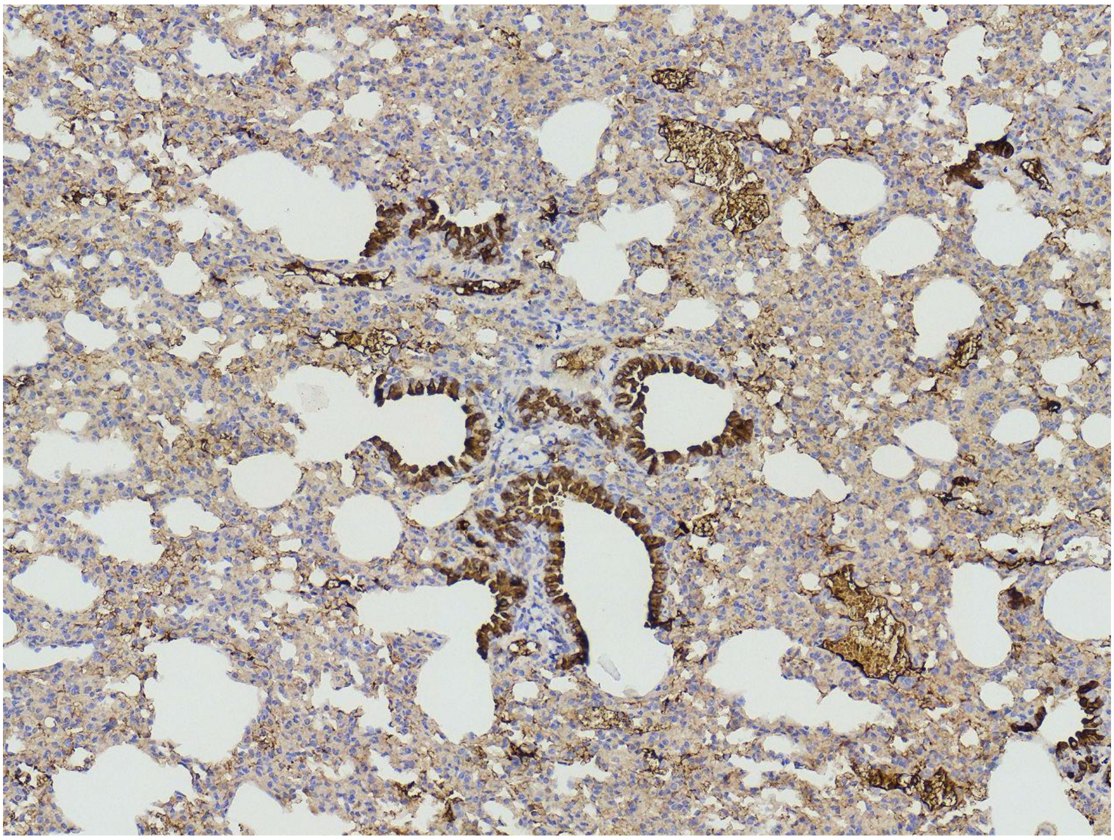

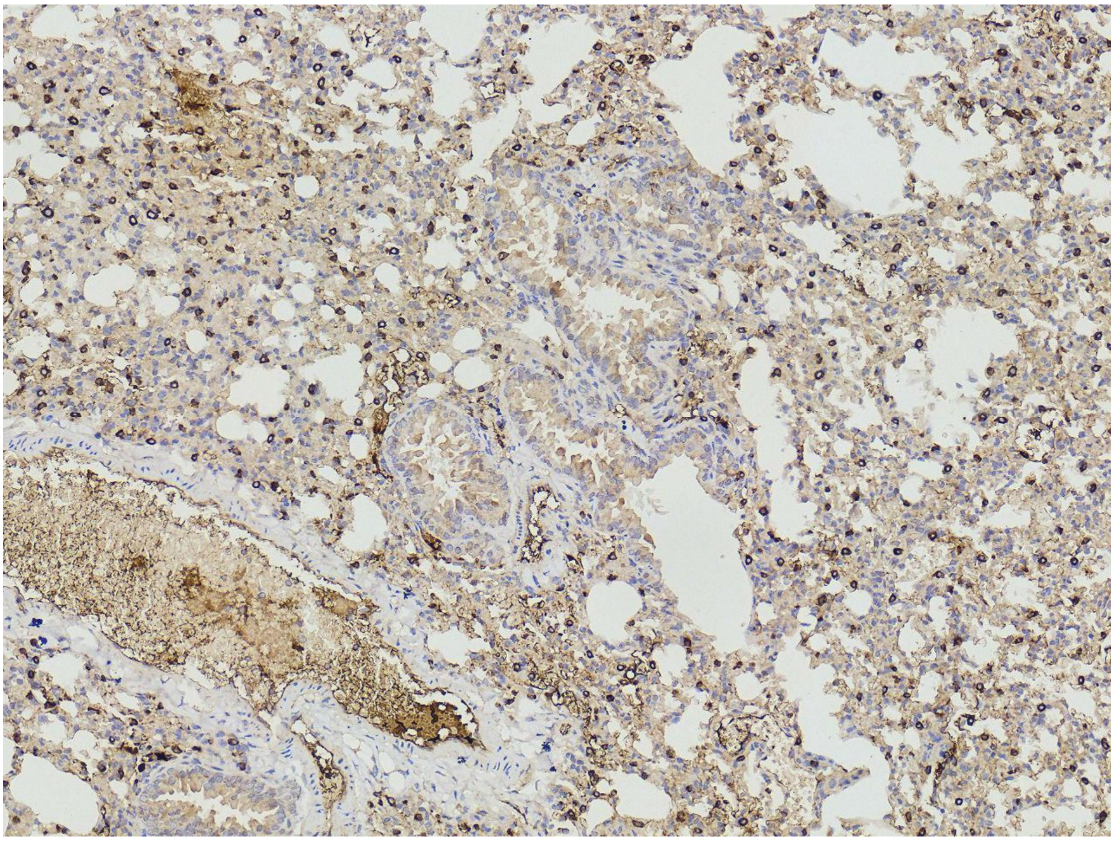

The original HE images in Figure 1J.

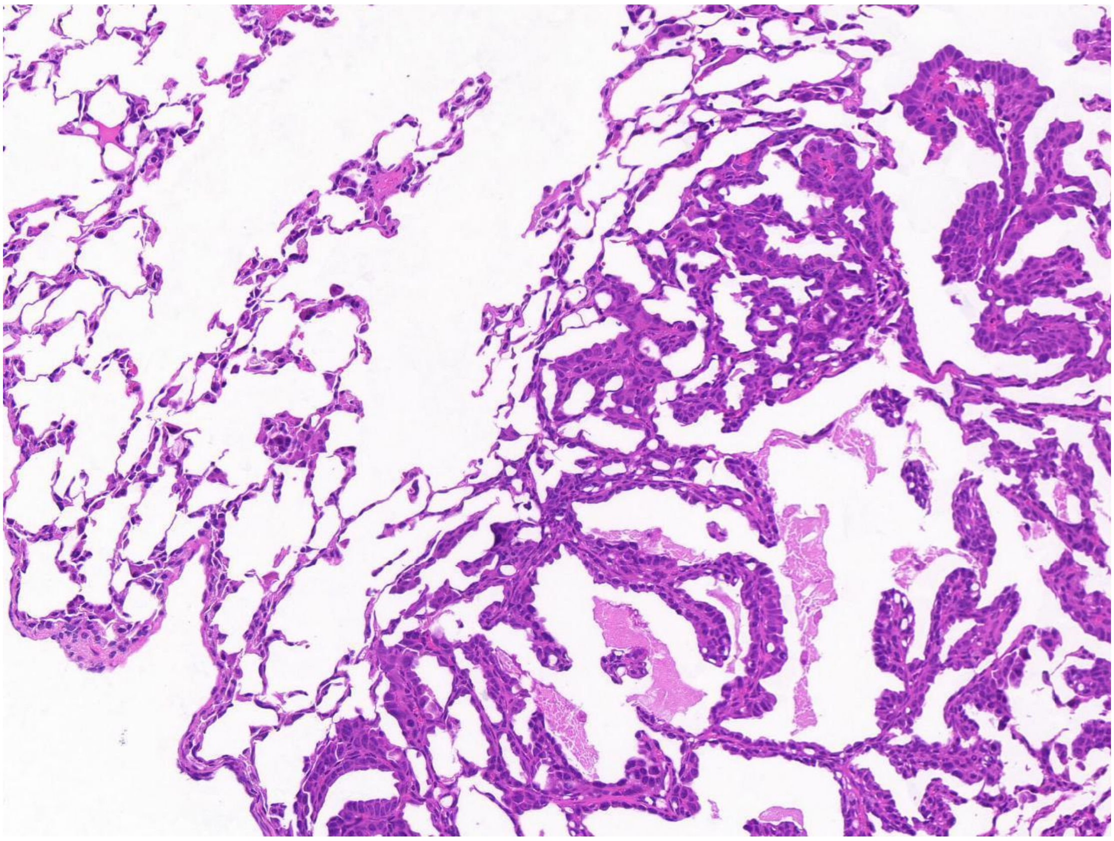

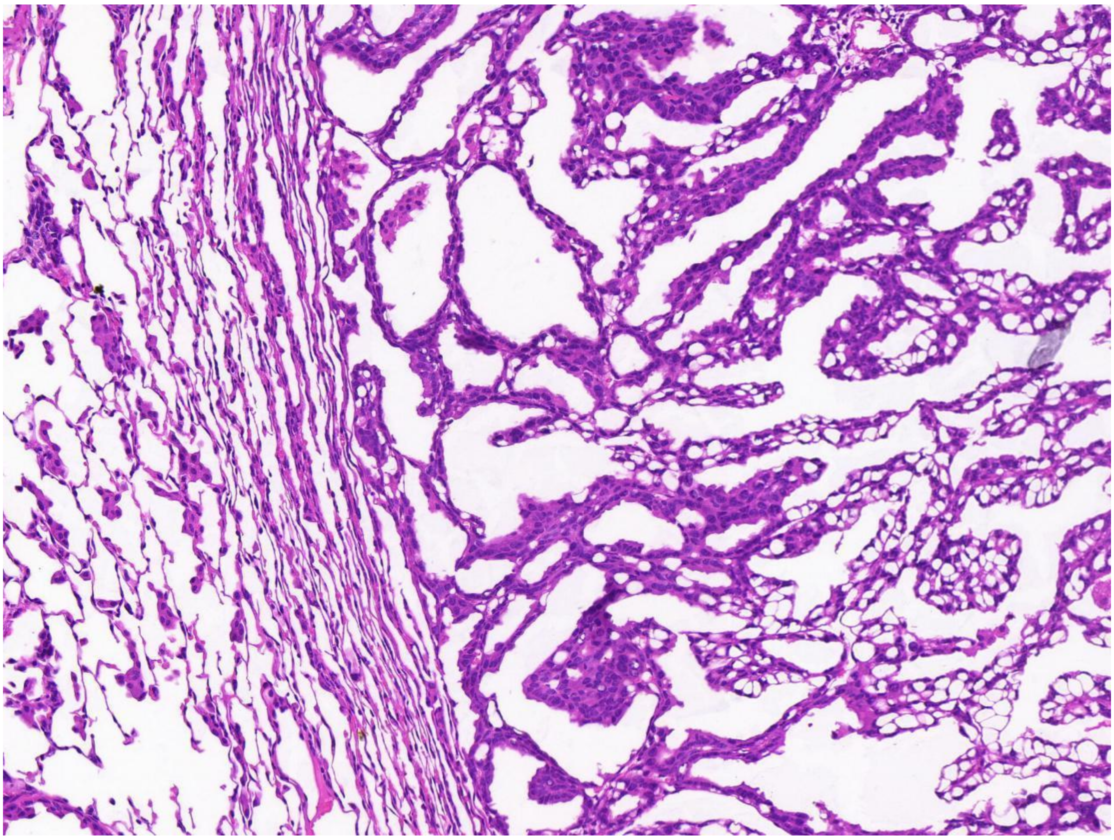

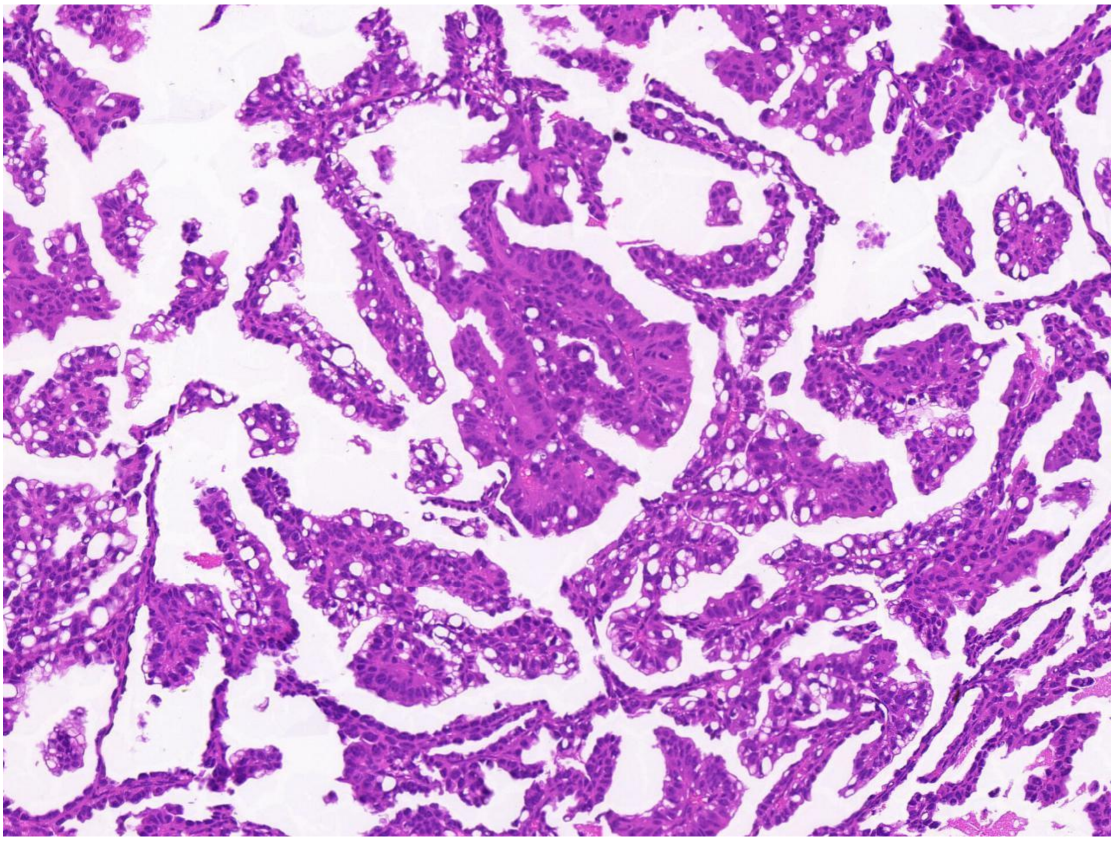

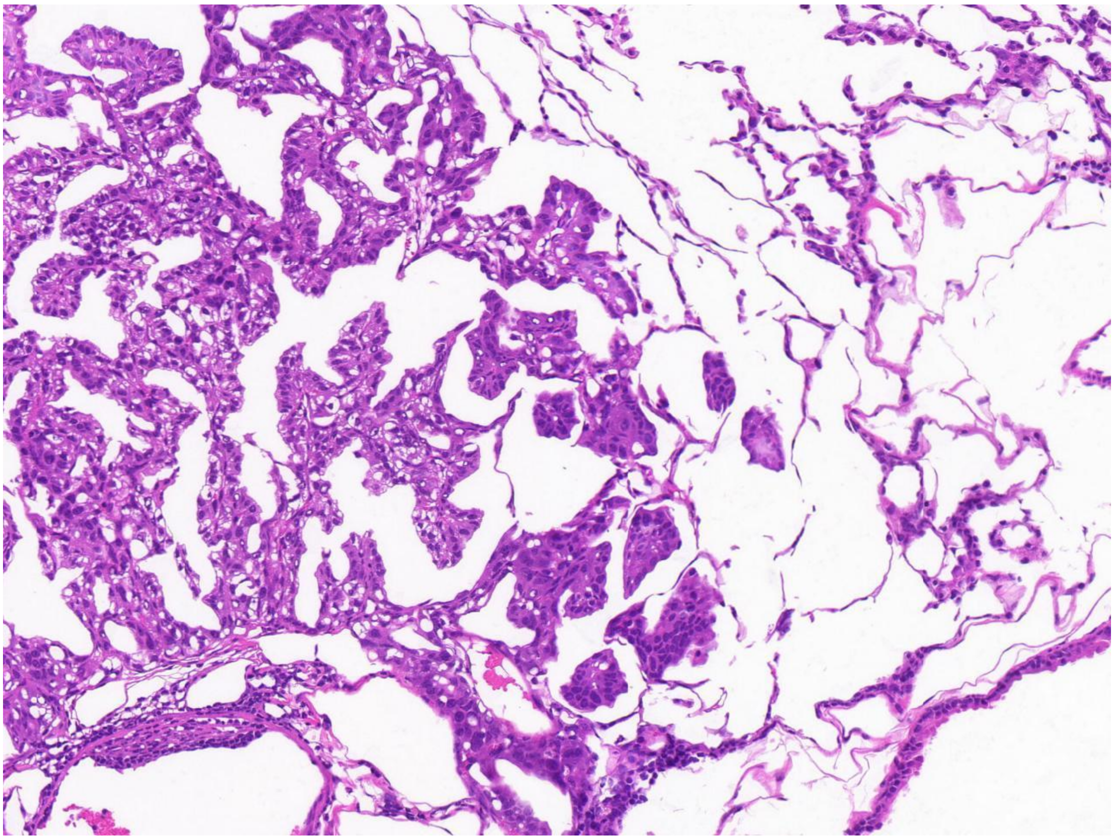

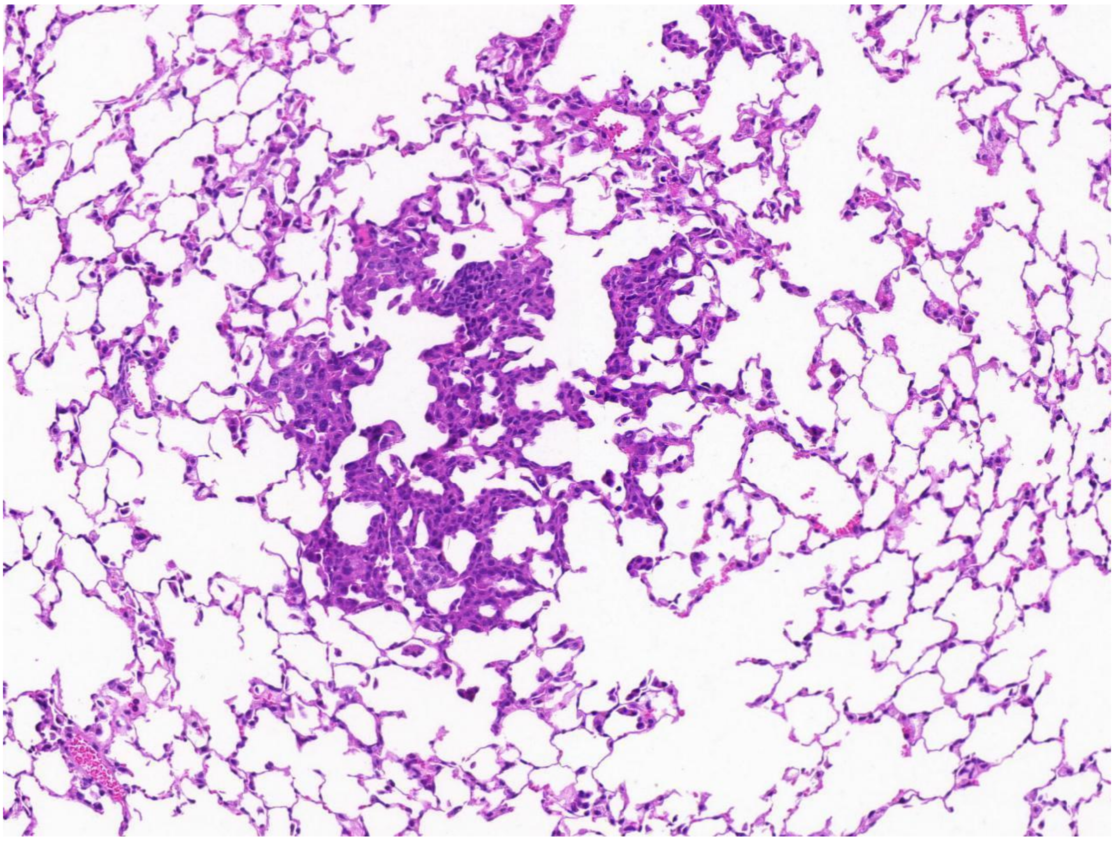

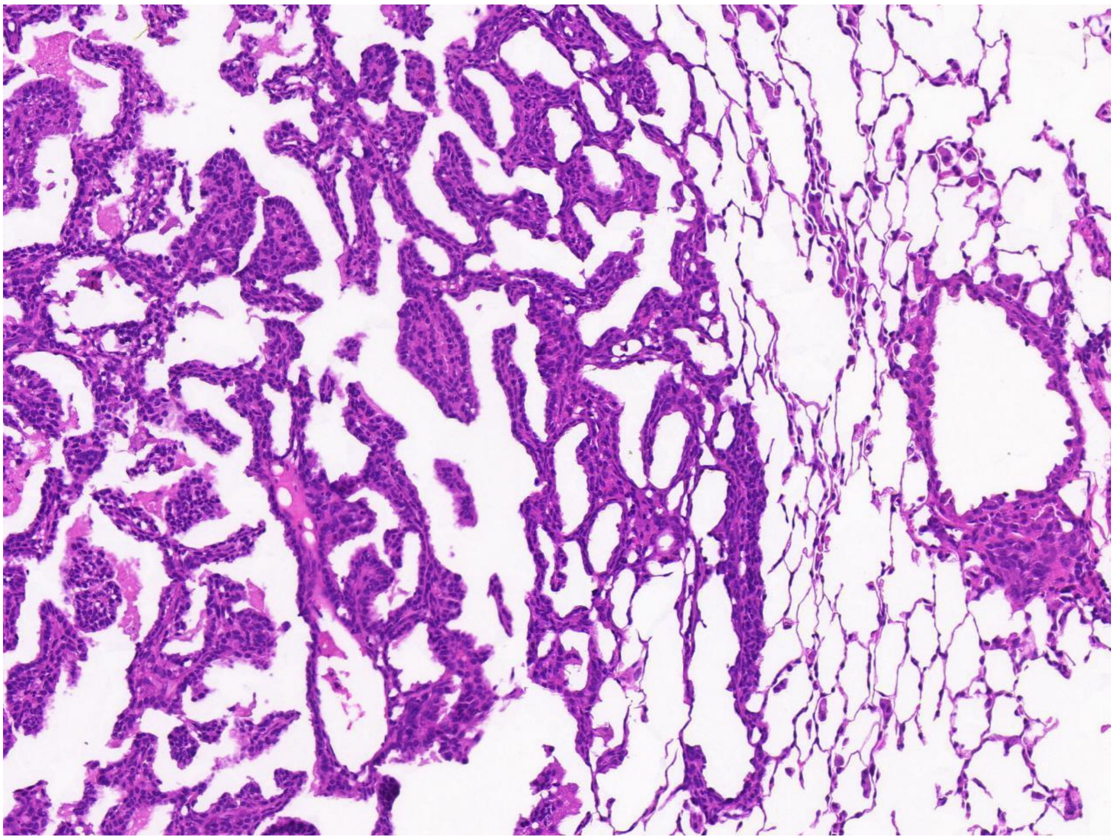

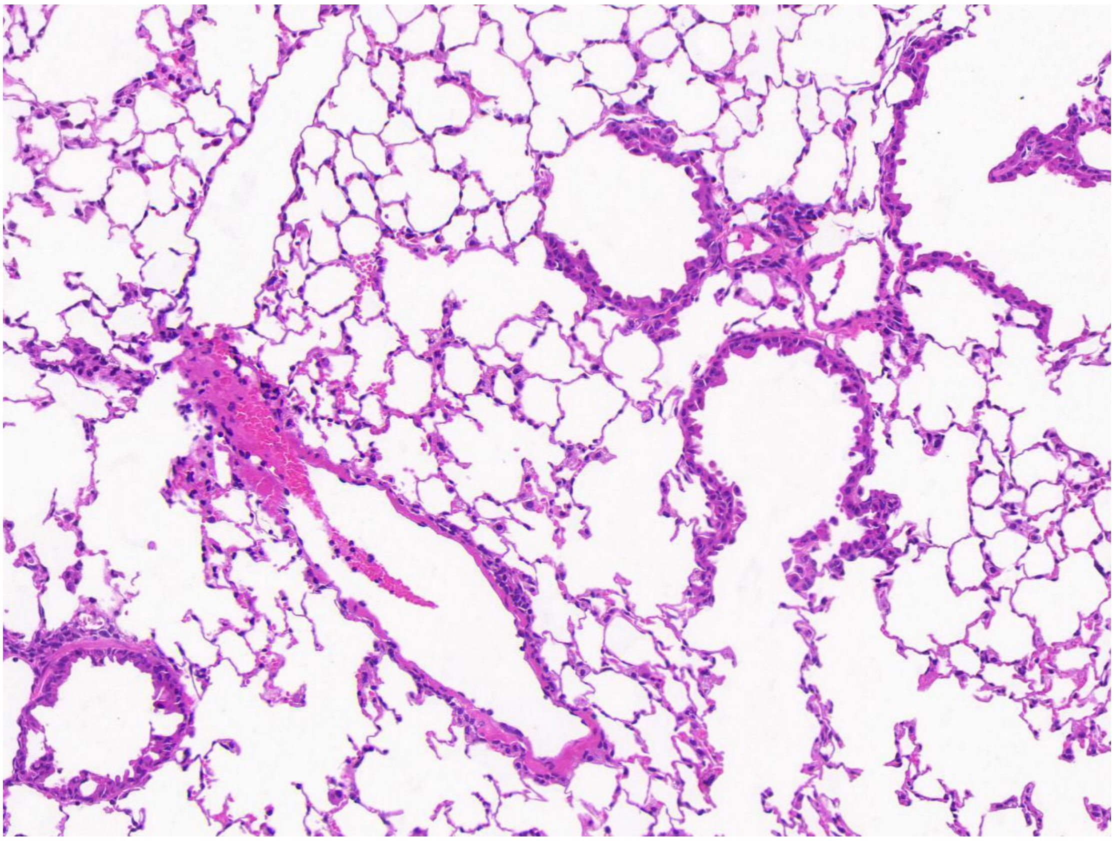
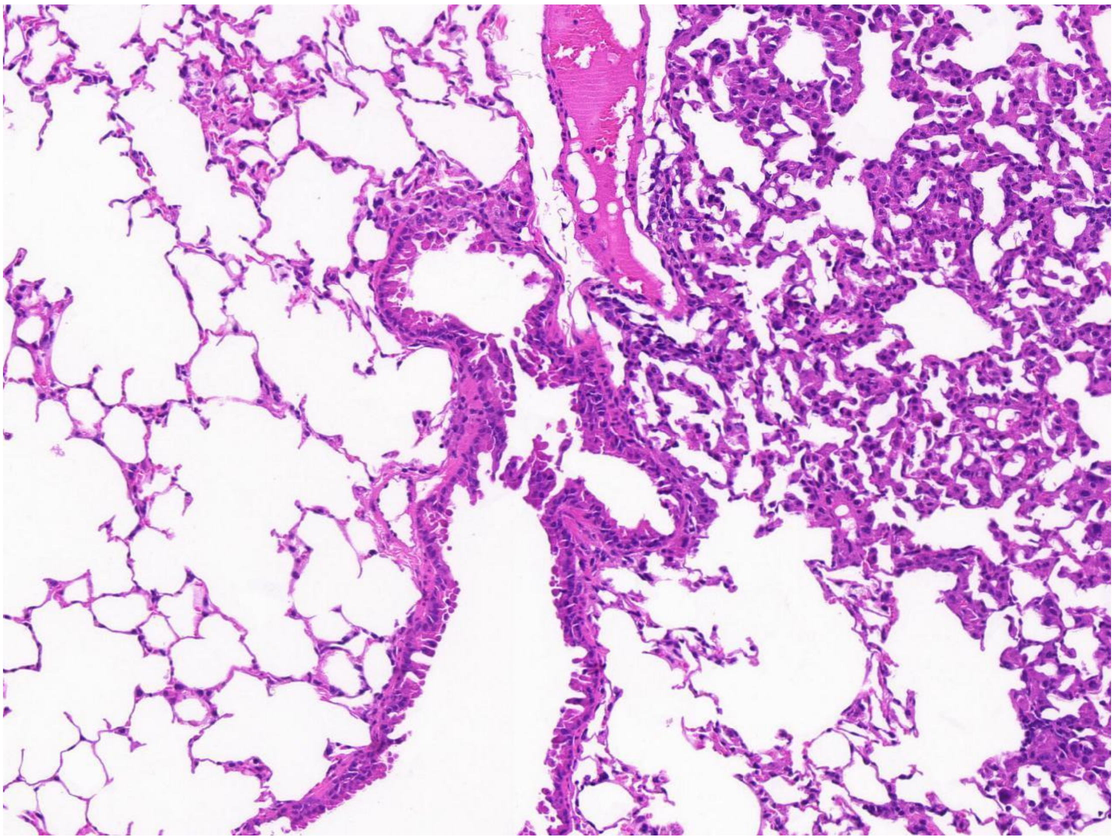

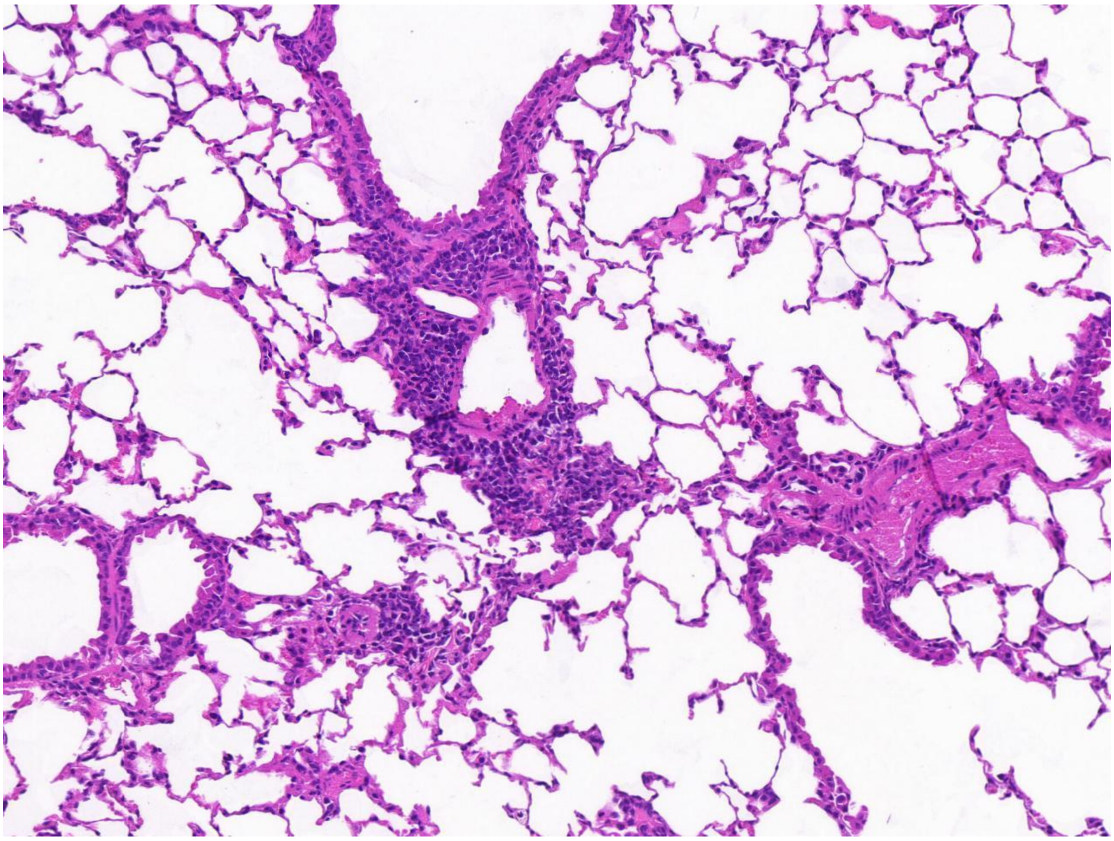

The original IHC images in Figure 2B.

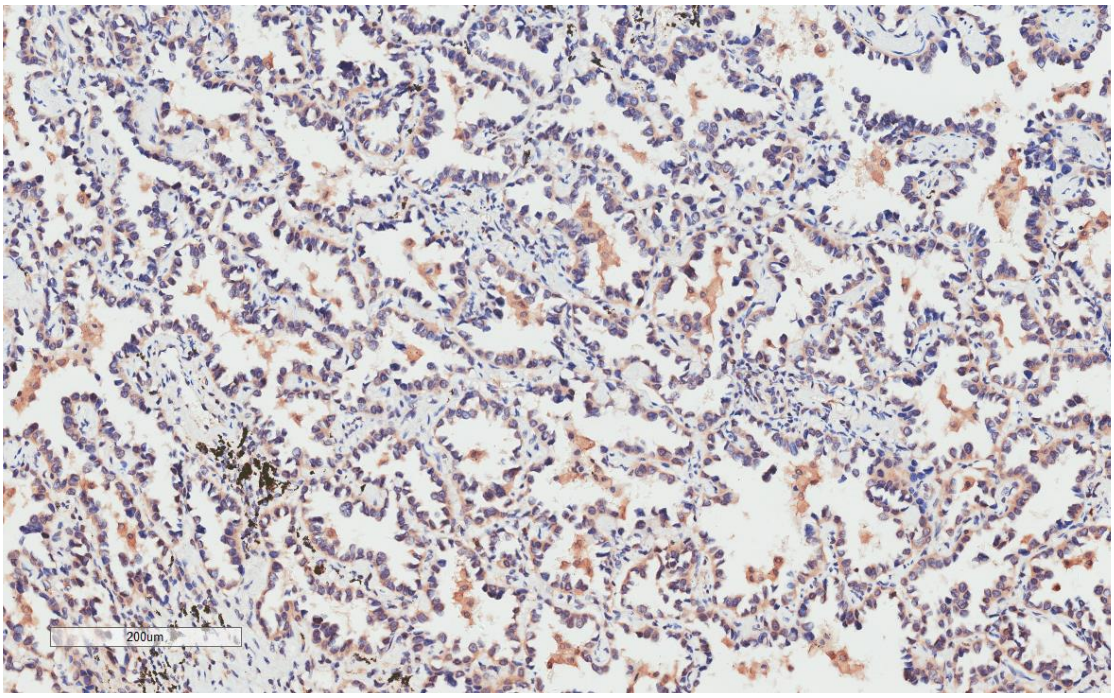

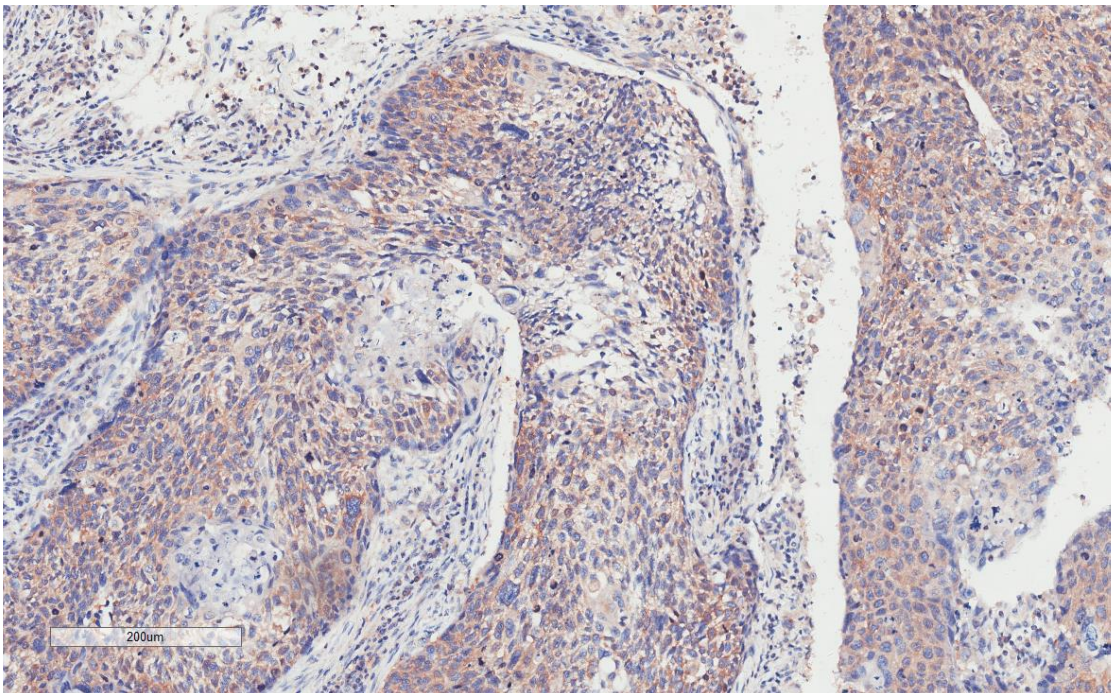

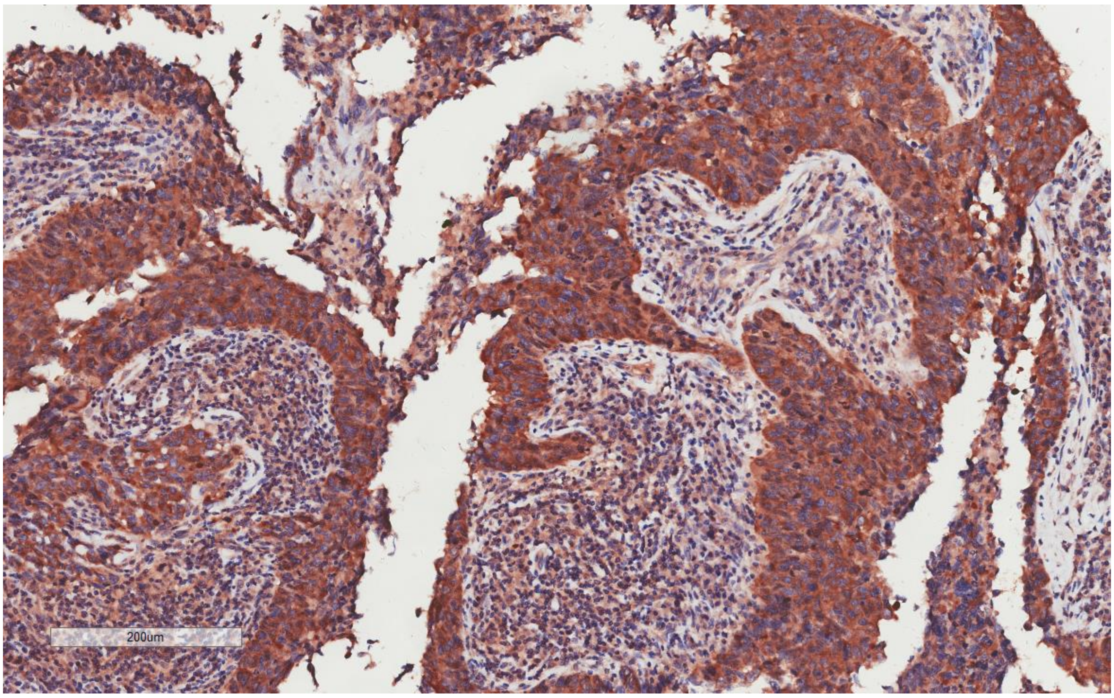
200um

The original IHC images in Figure 2C.

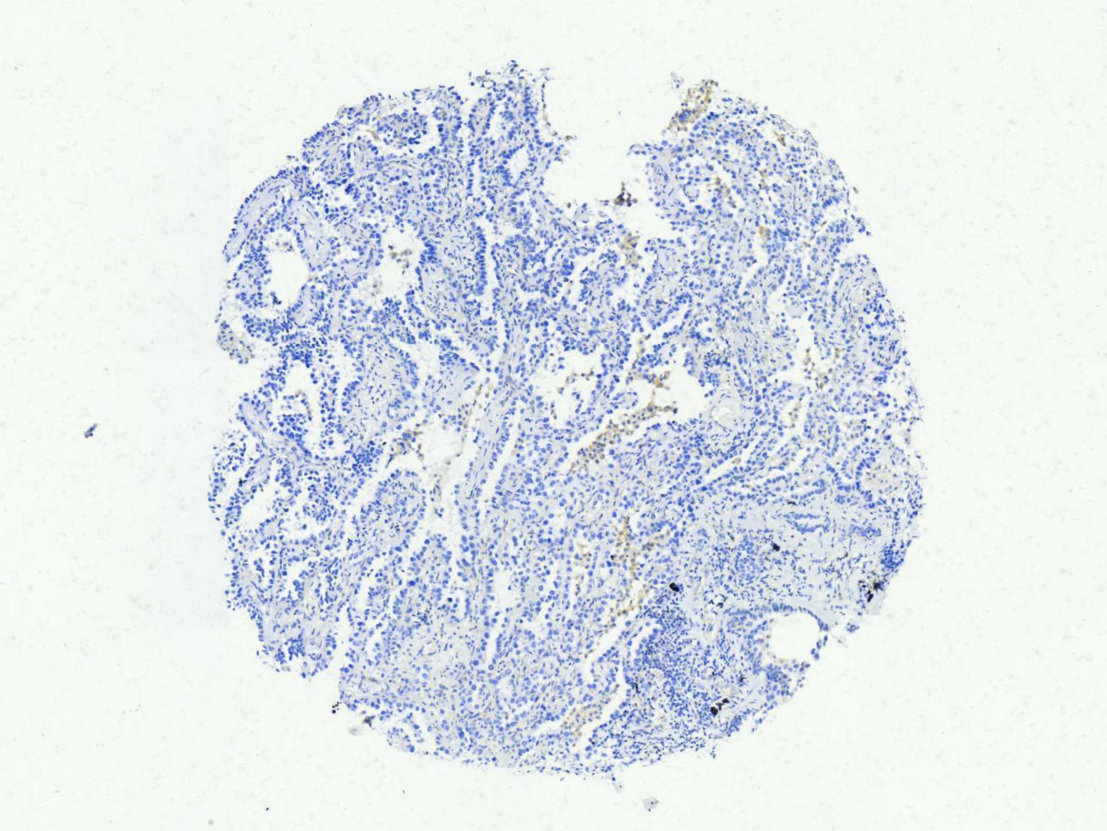

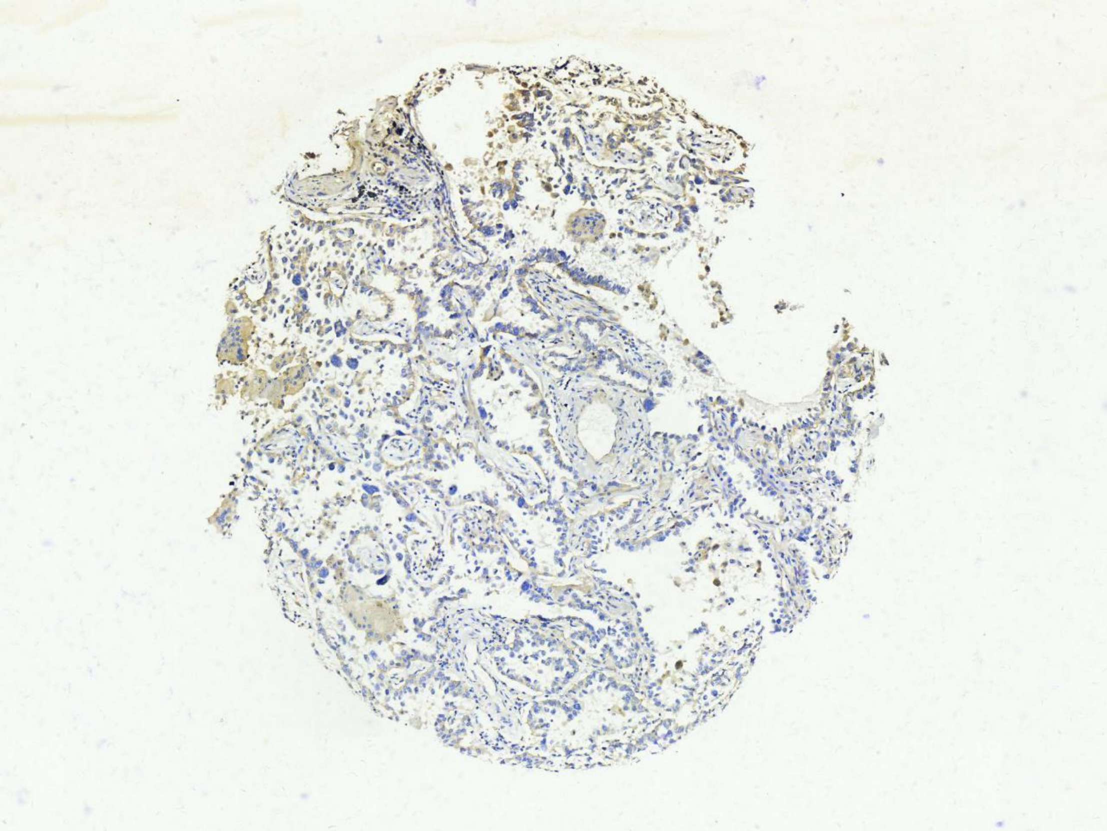

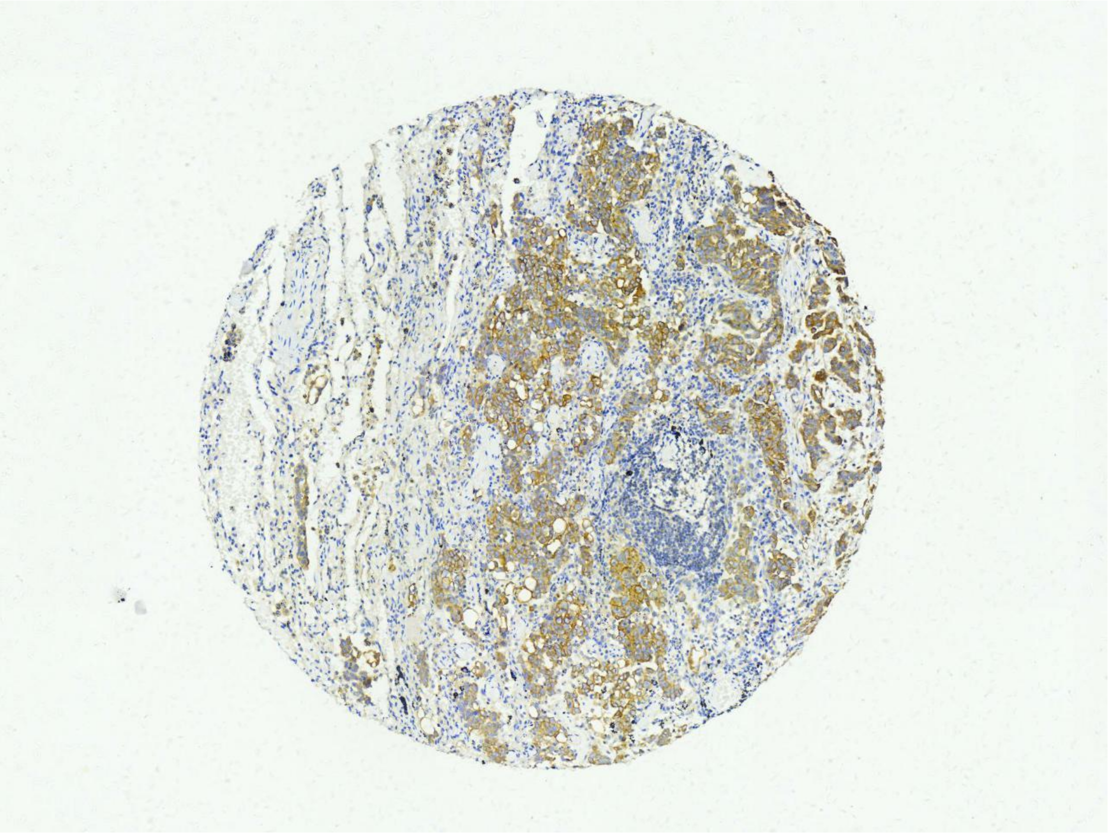

The original HE and IHC images in Figure 2H.

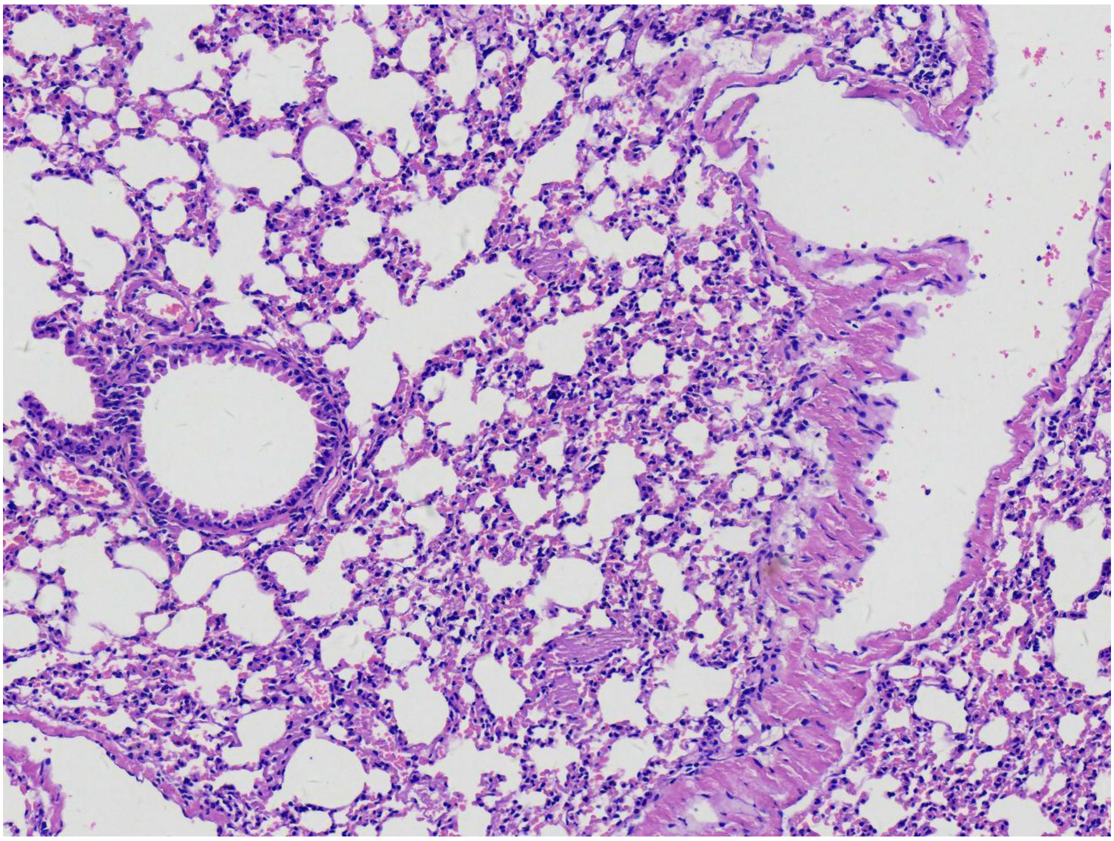

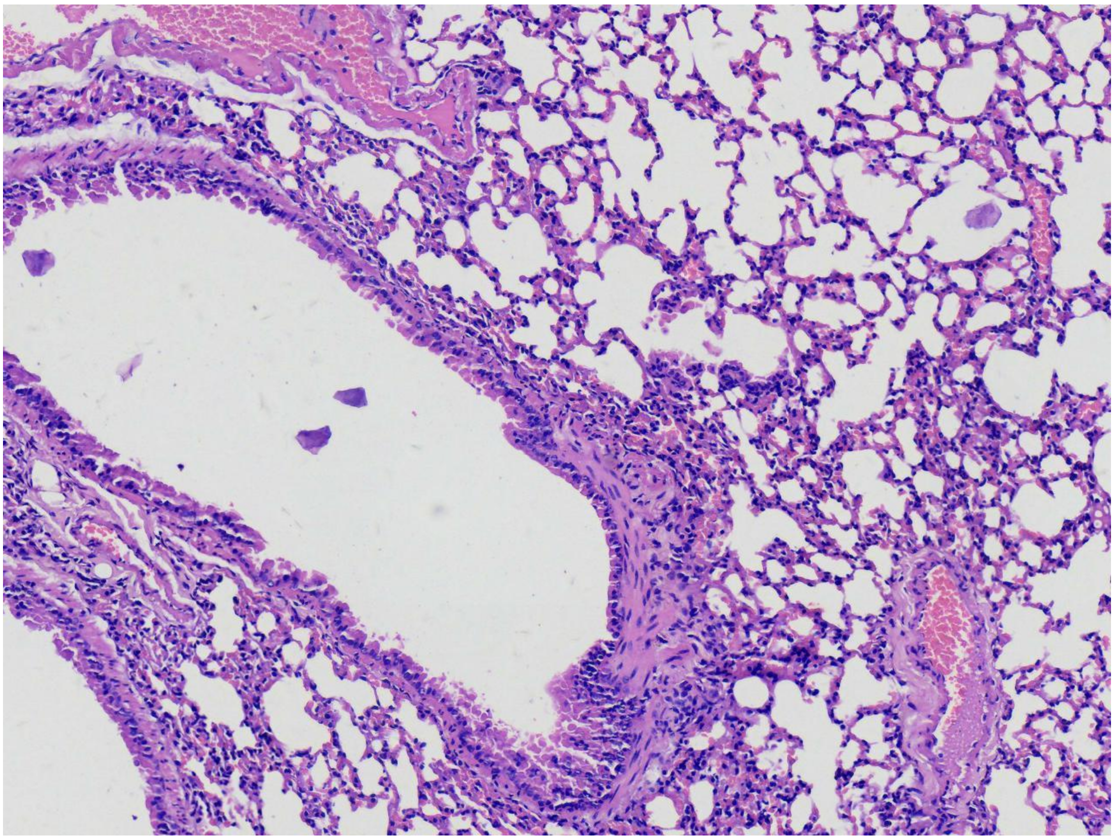

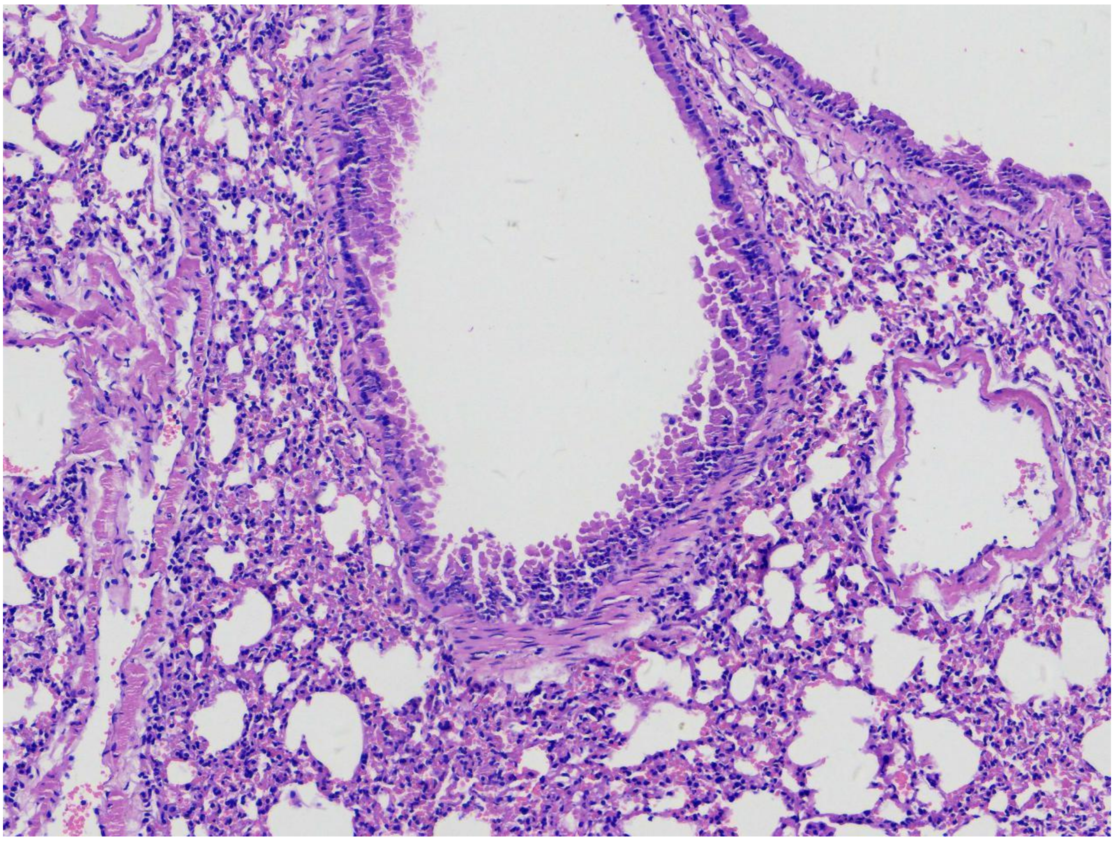

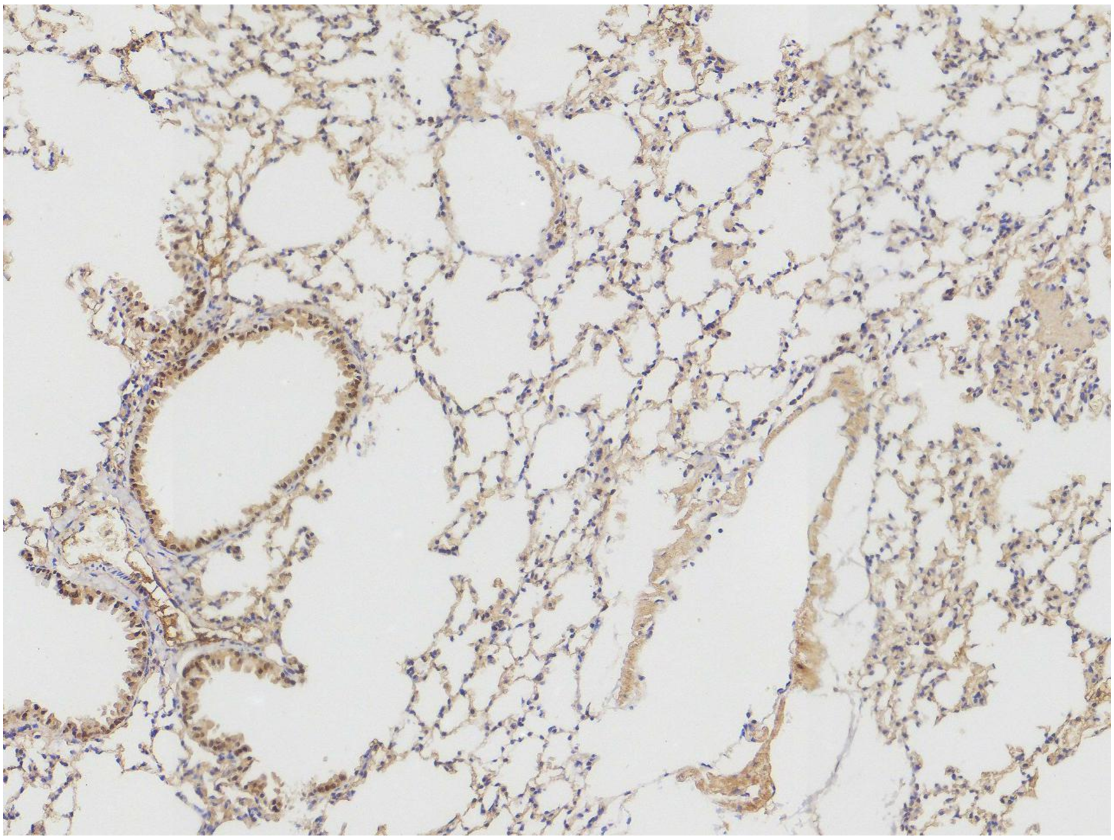

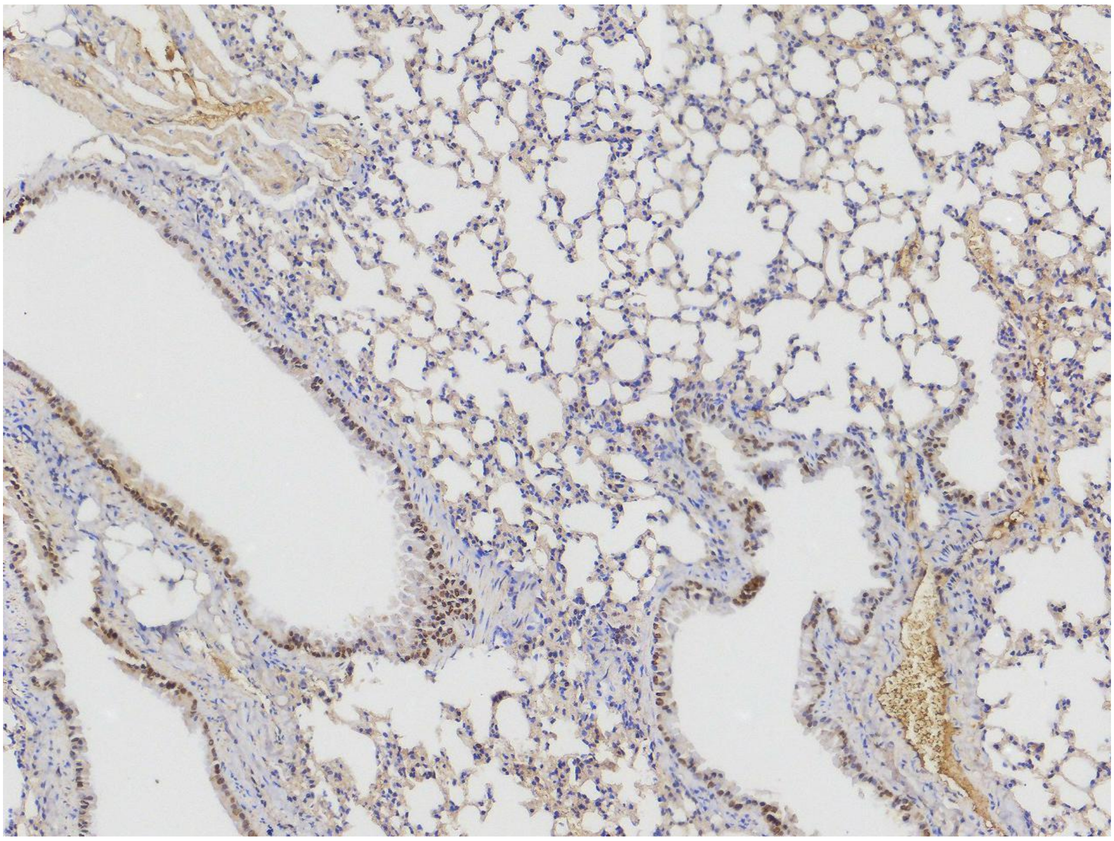

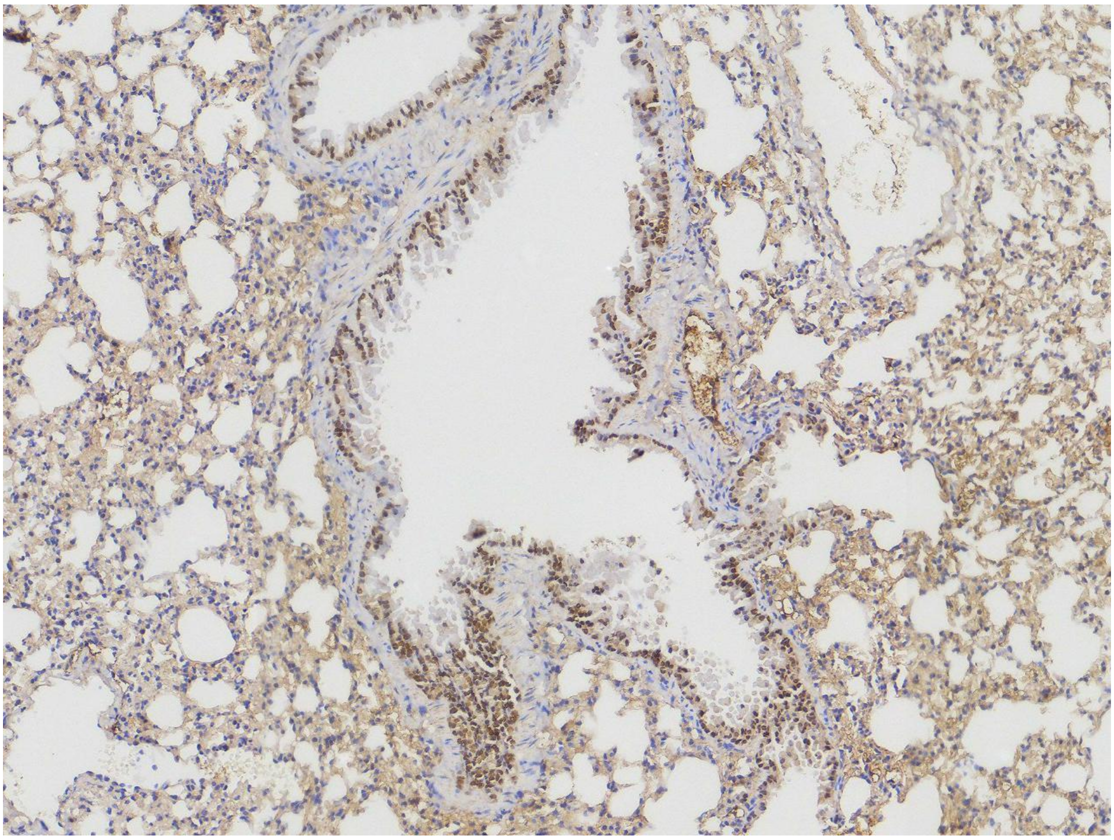

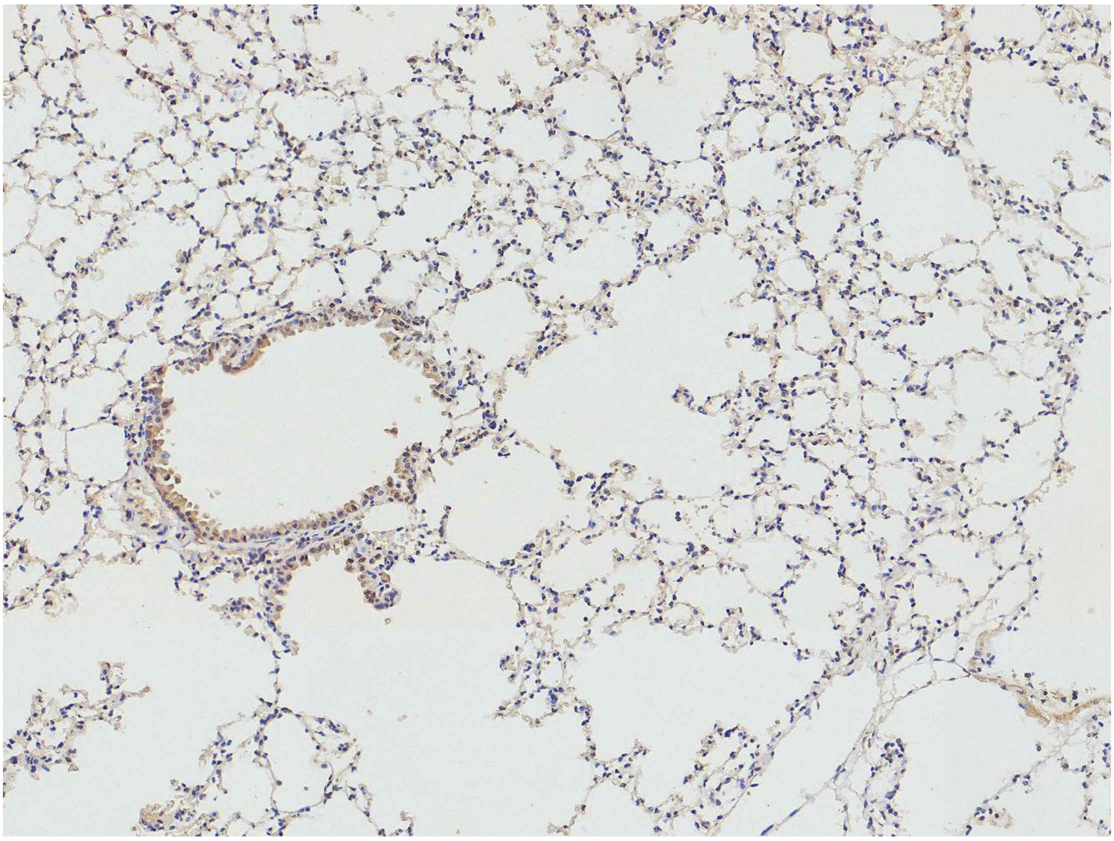
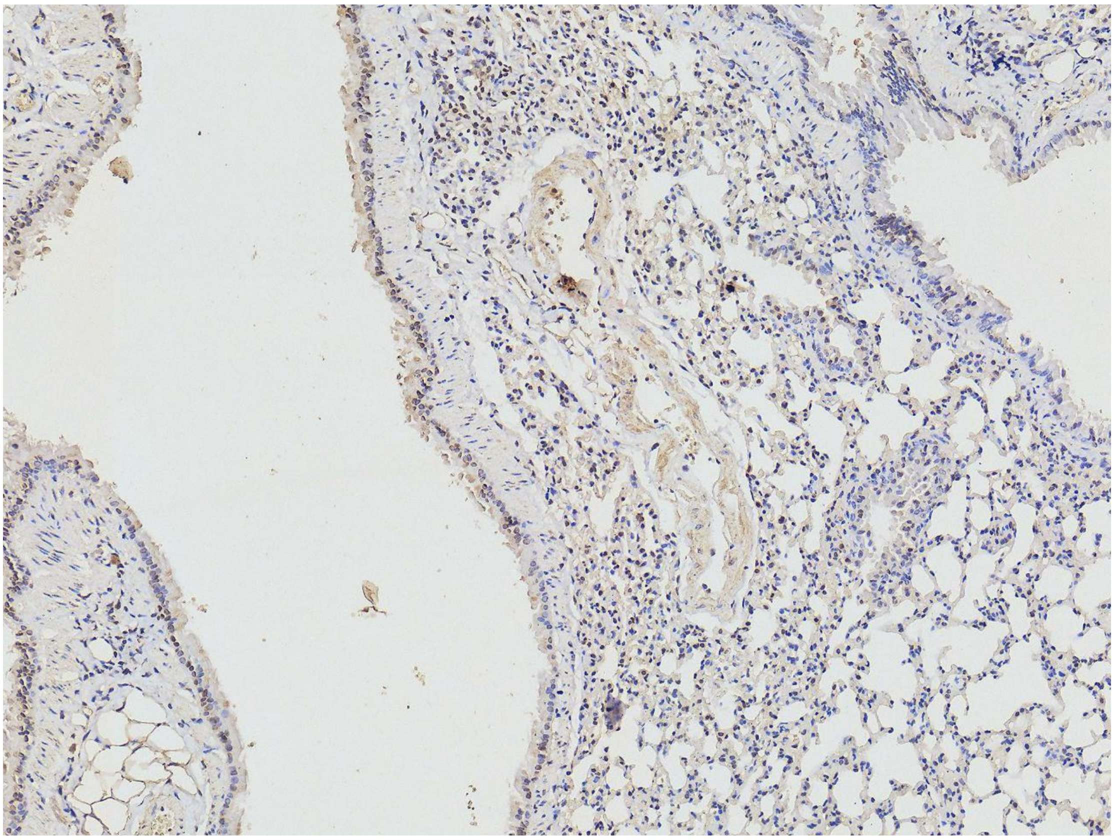

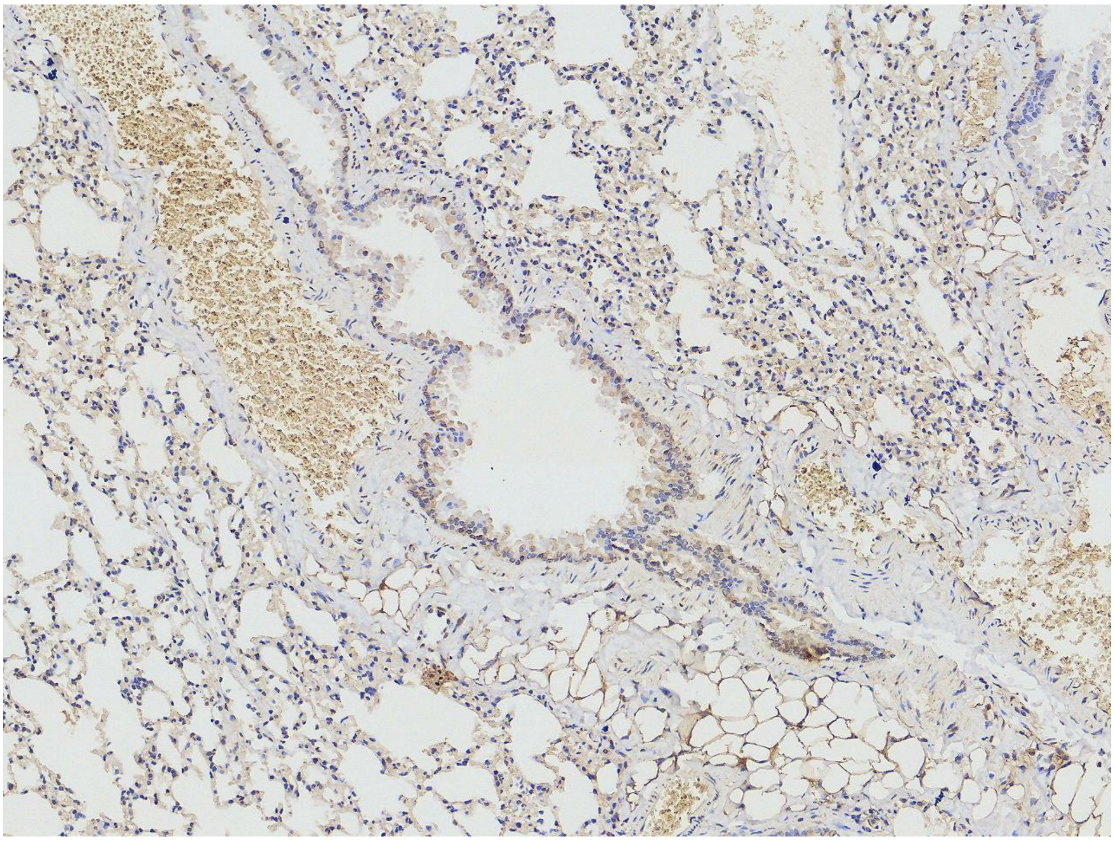

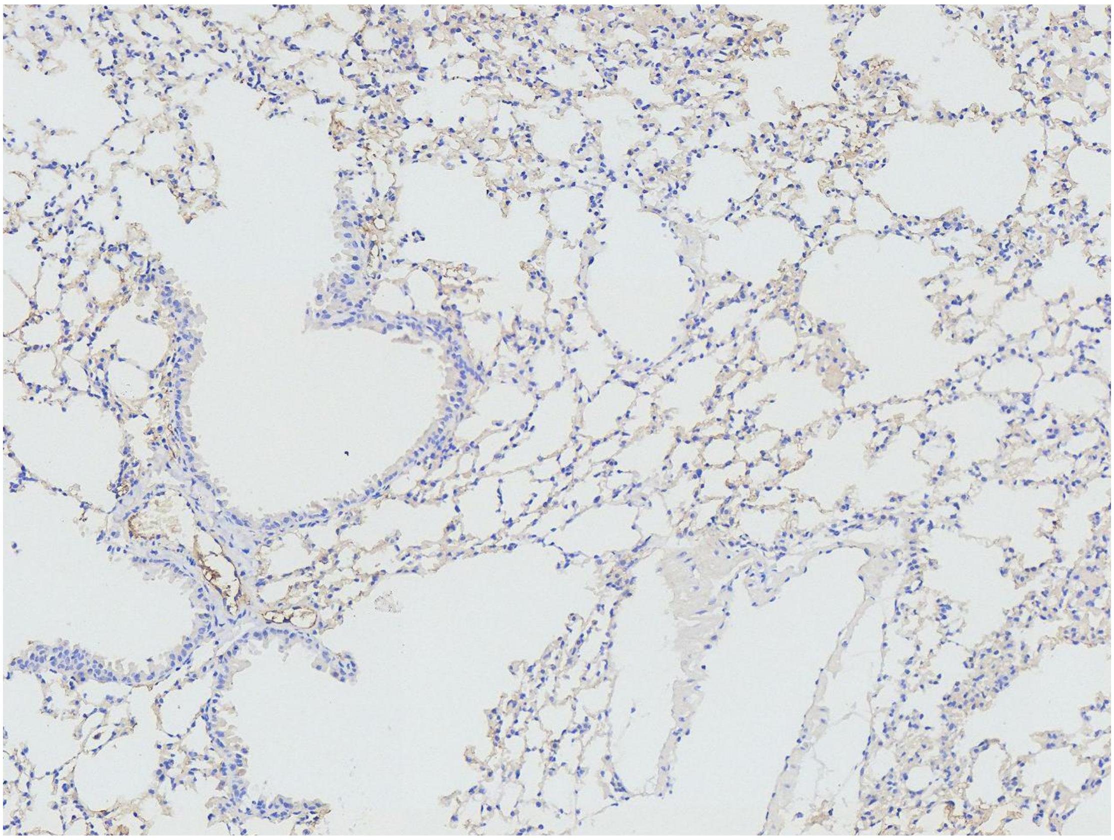

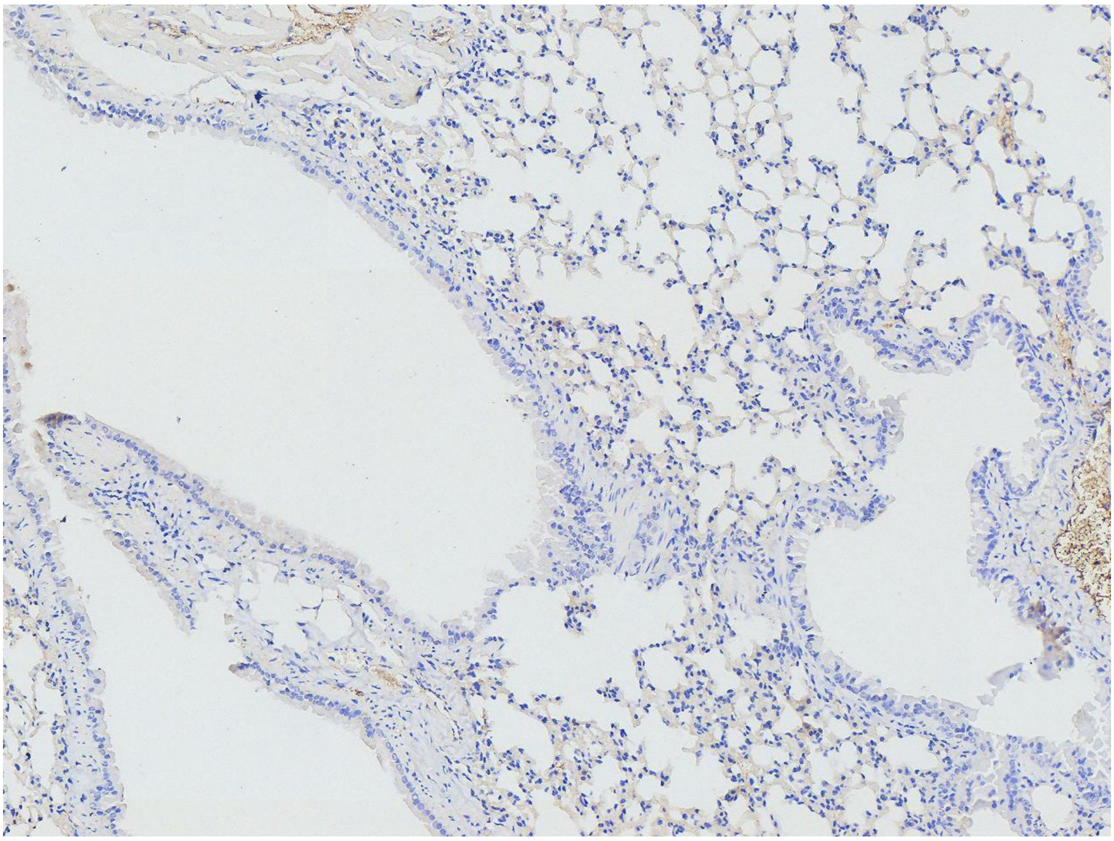

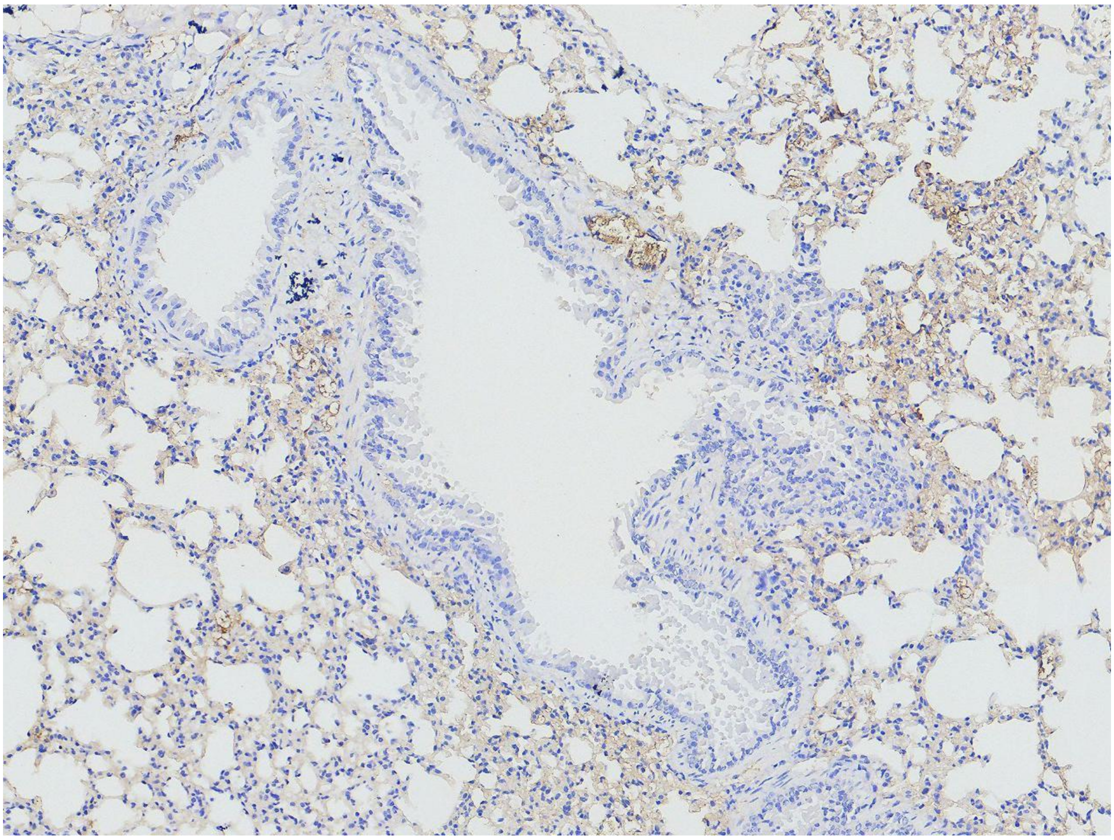

The original HE and IHC images in Figure 2I.

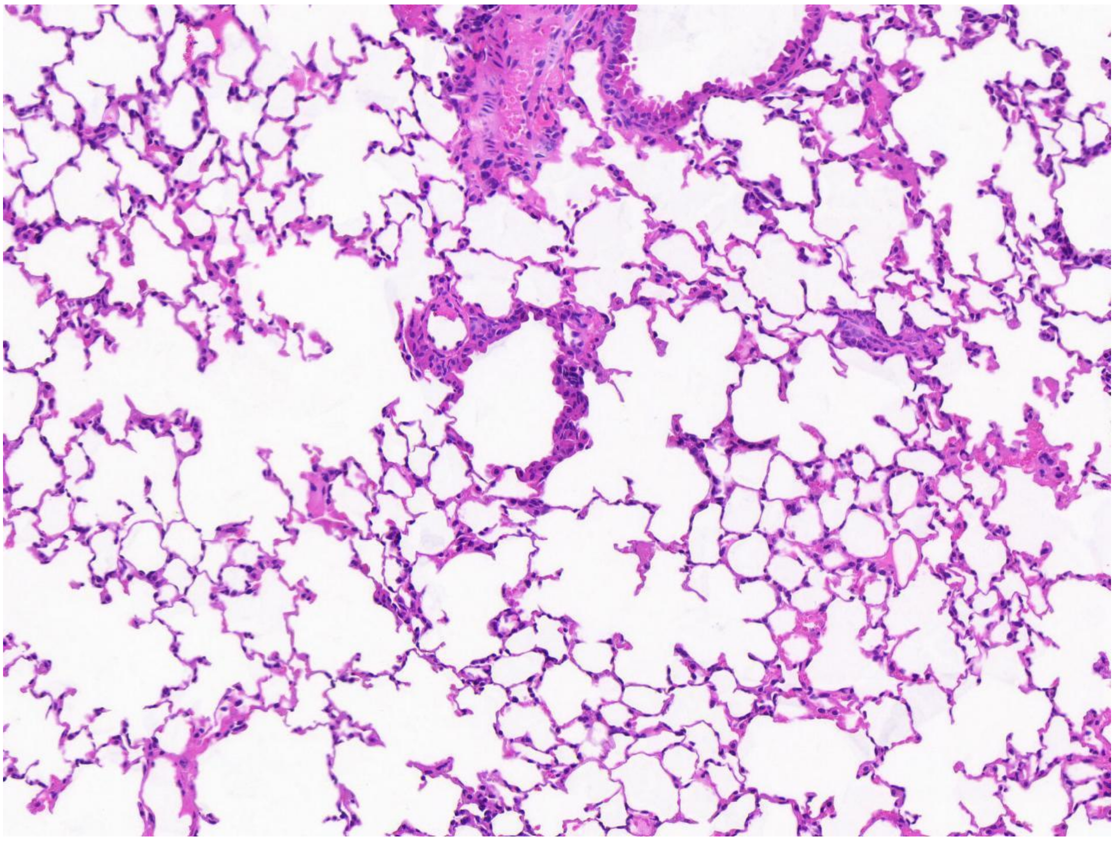

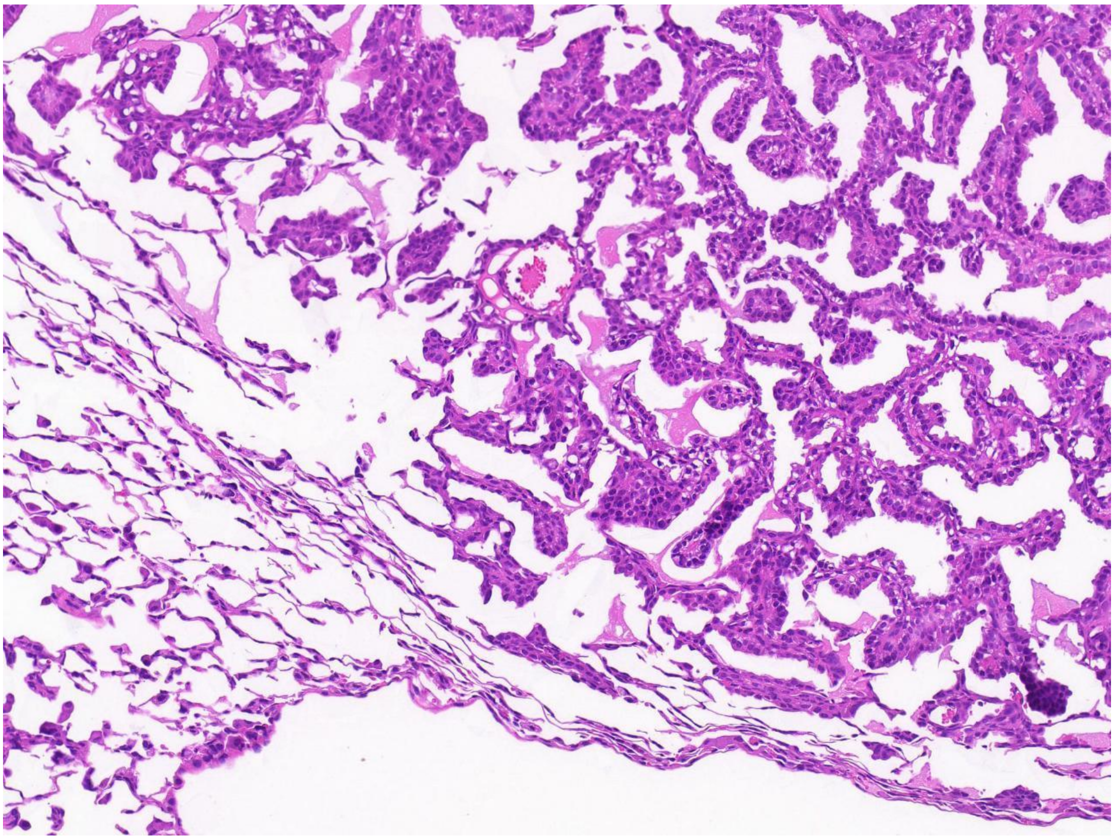

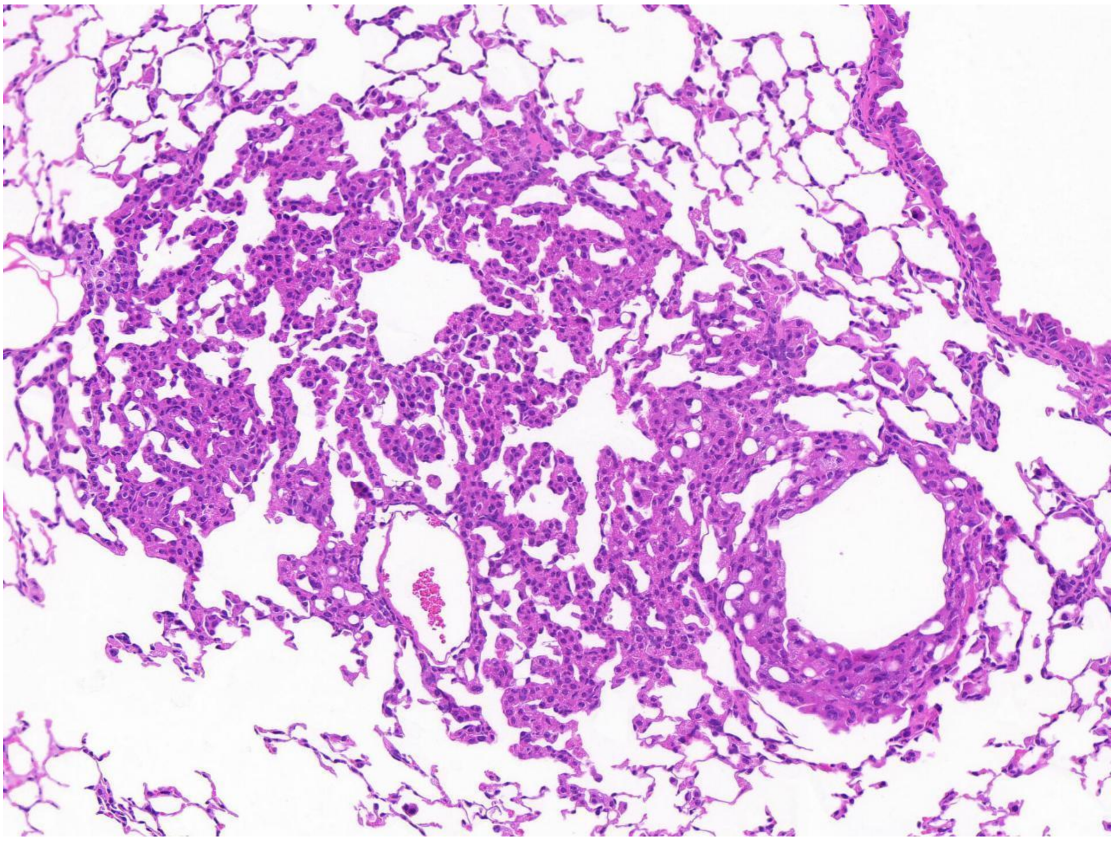

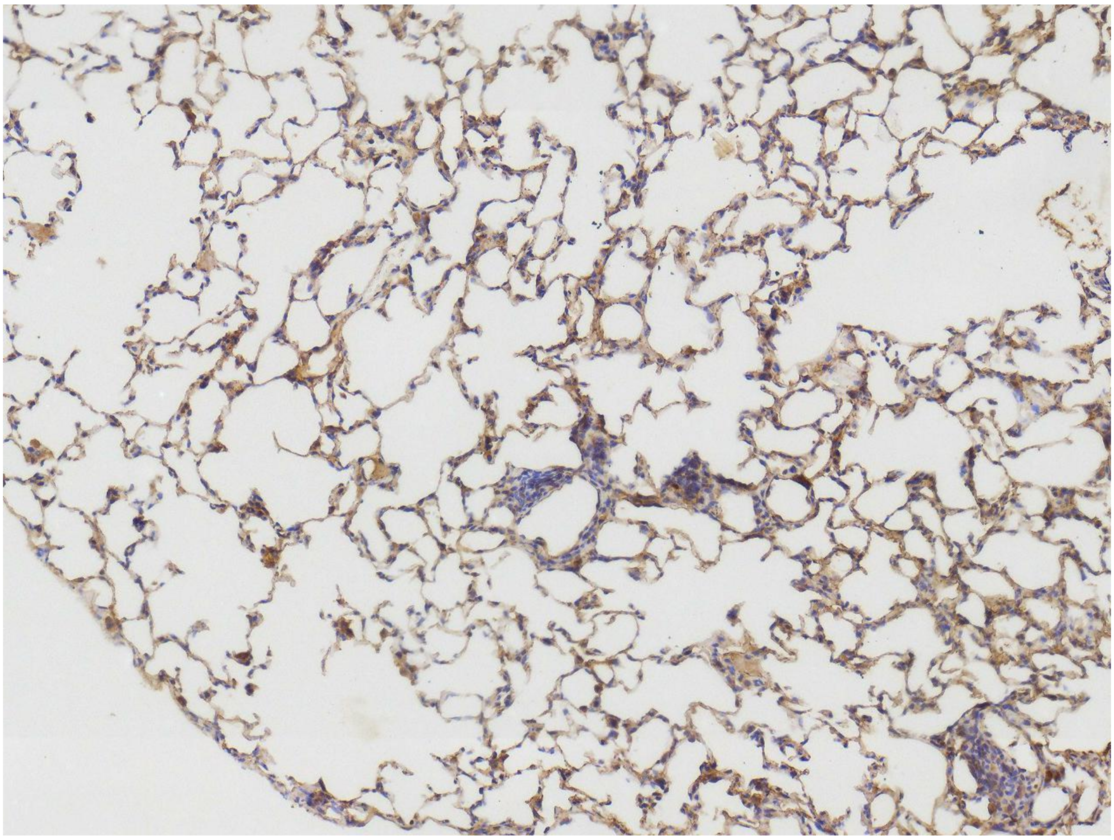

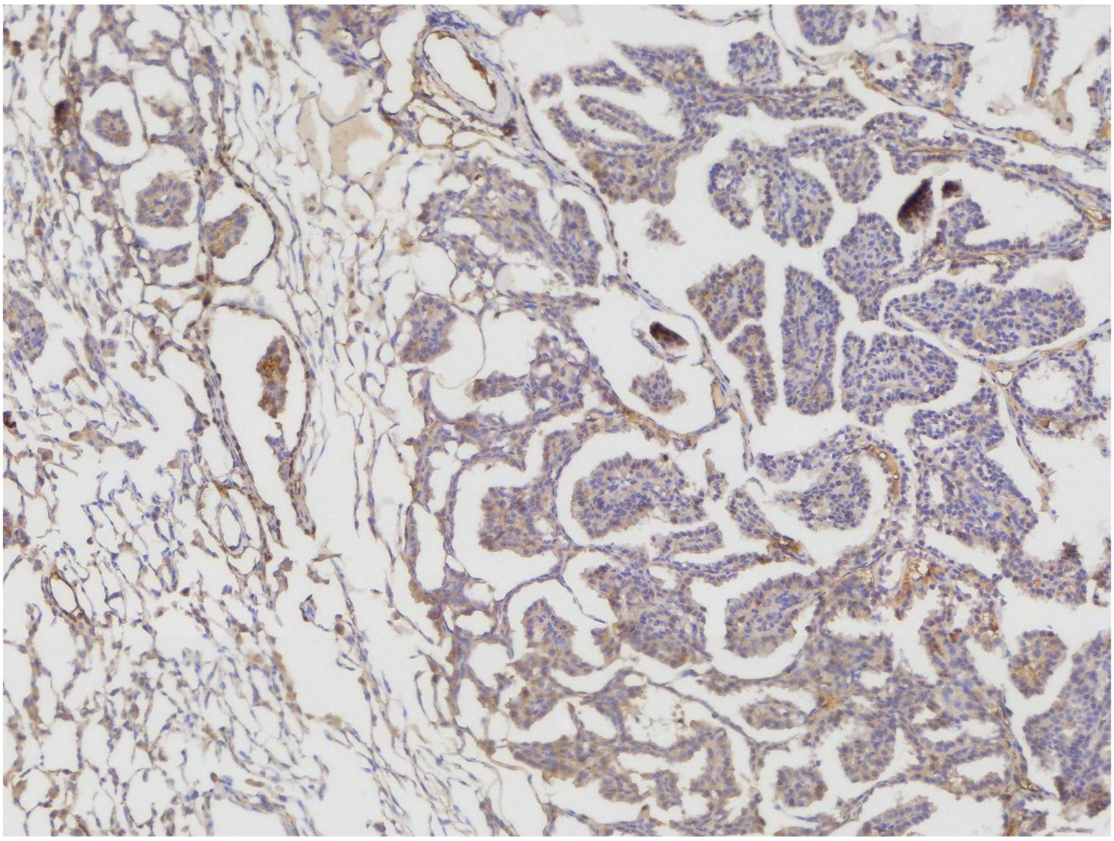

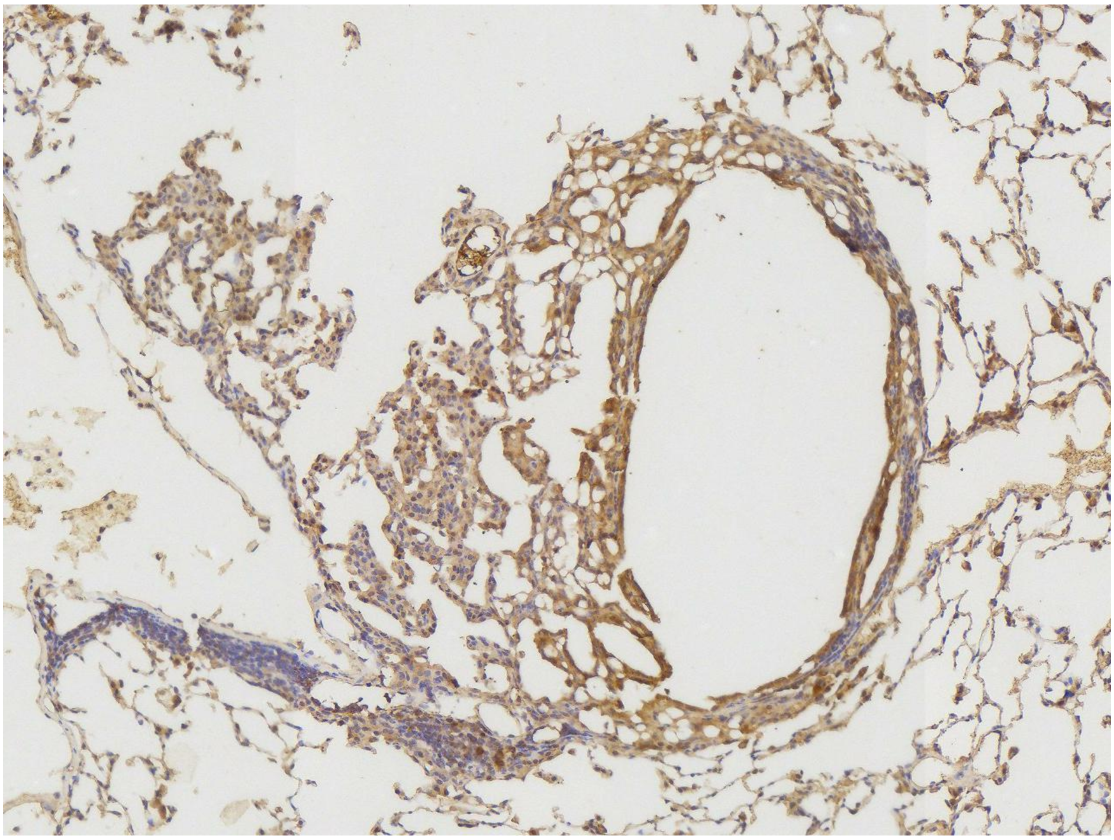

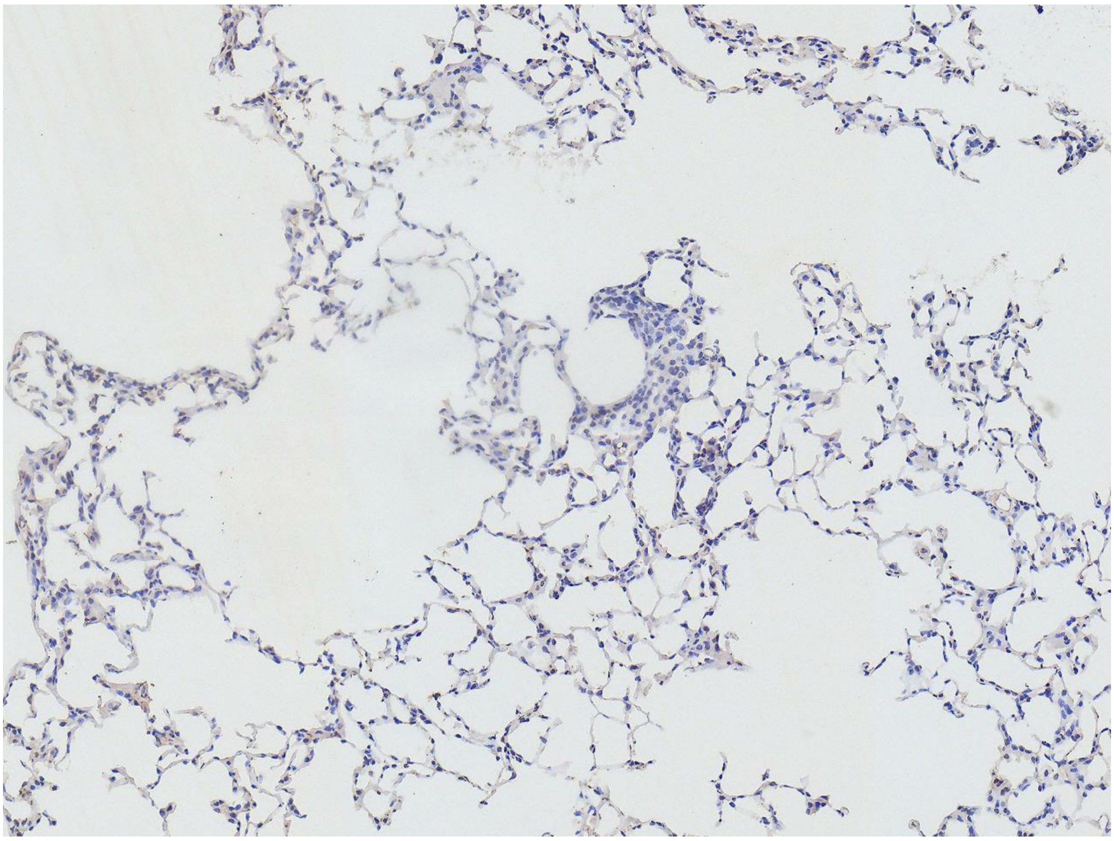

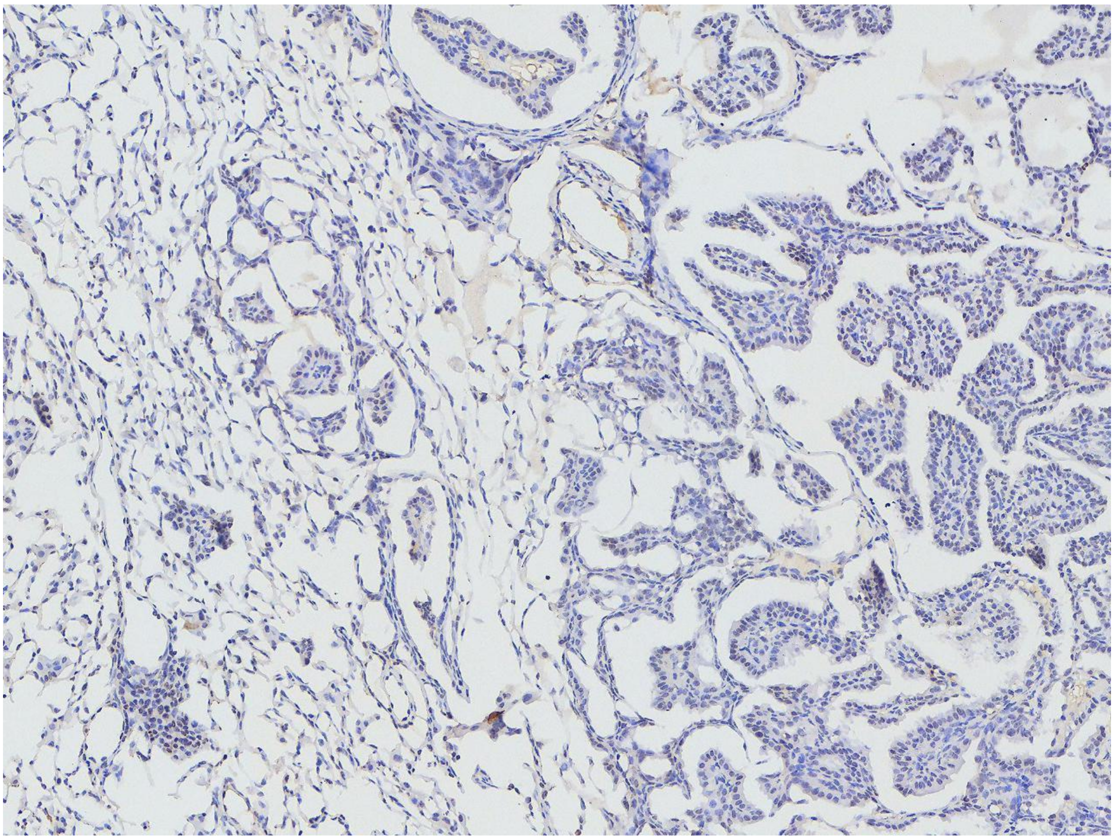

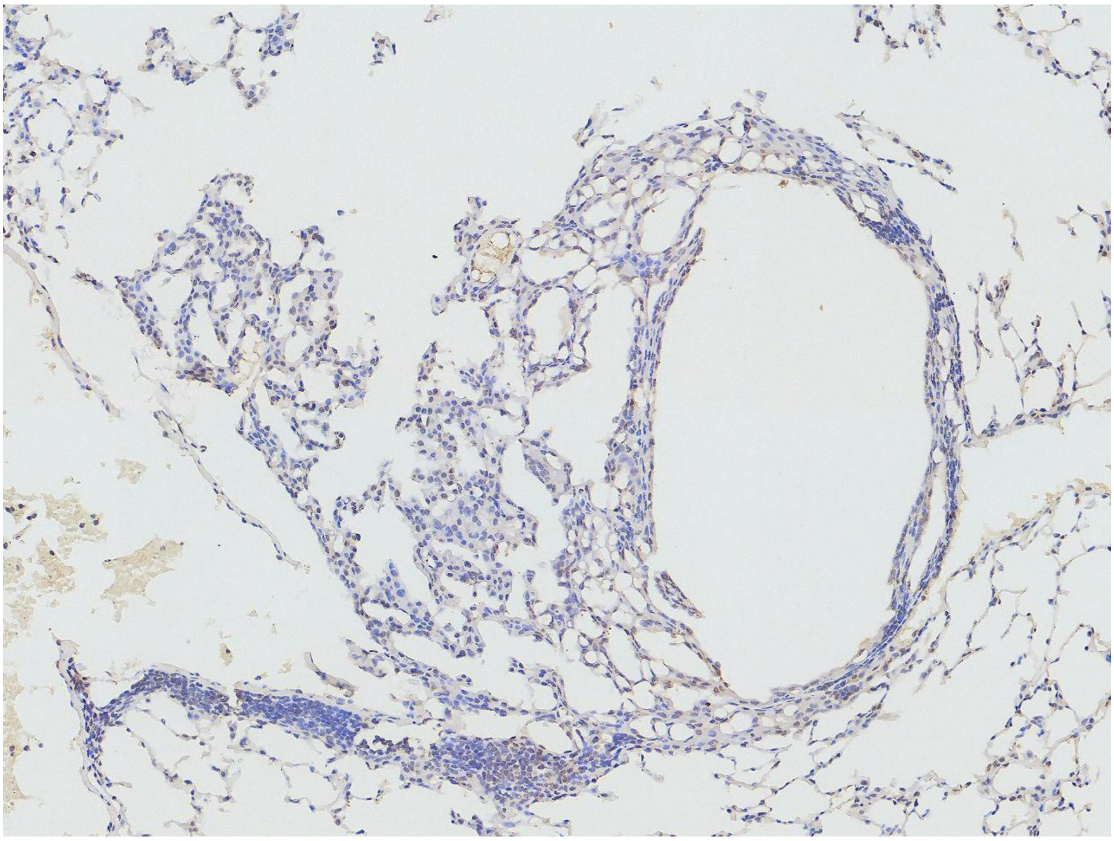

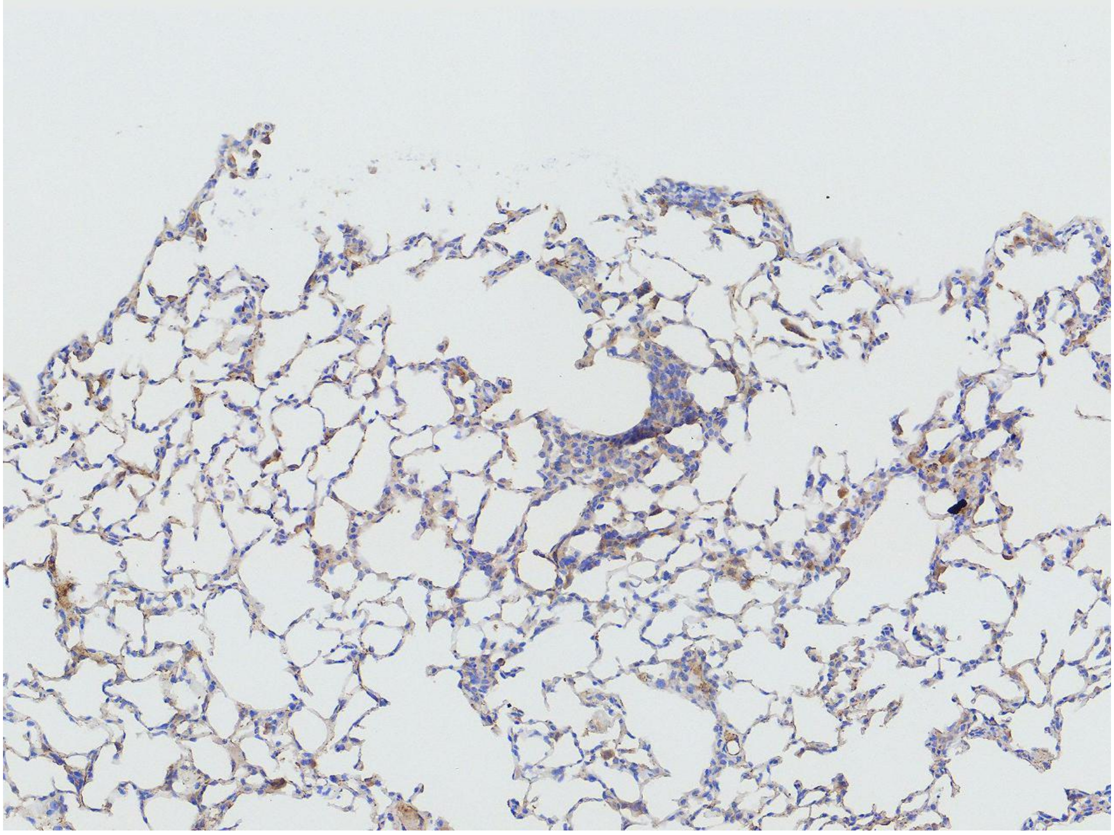

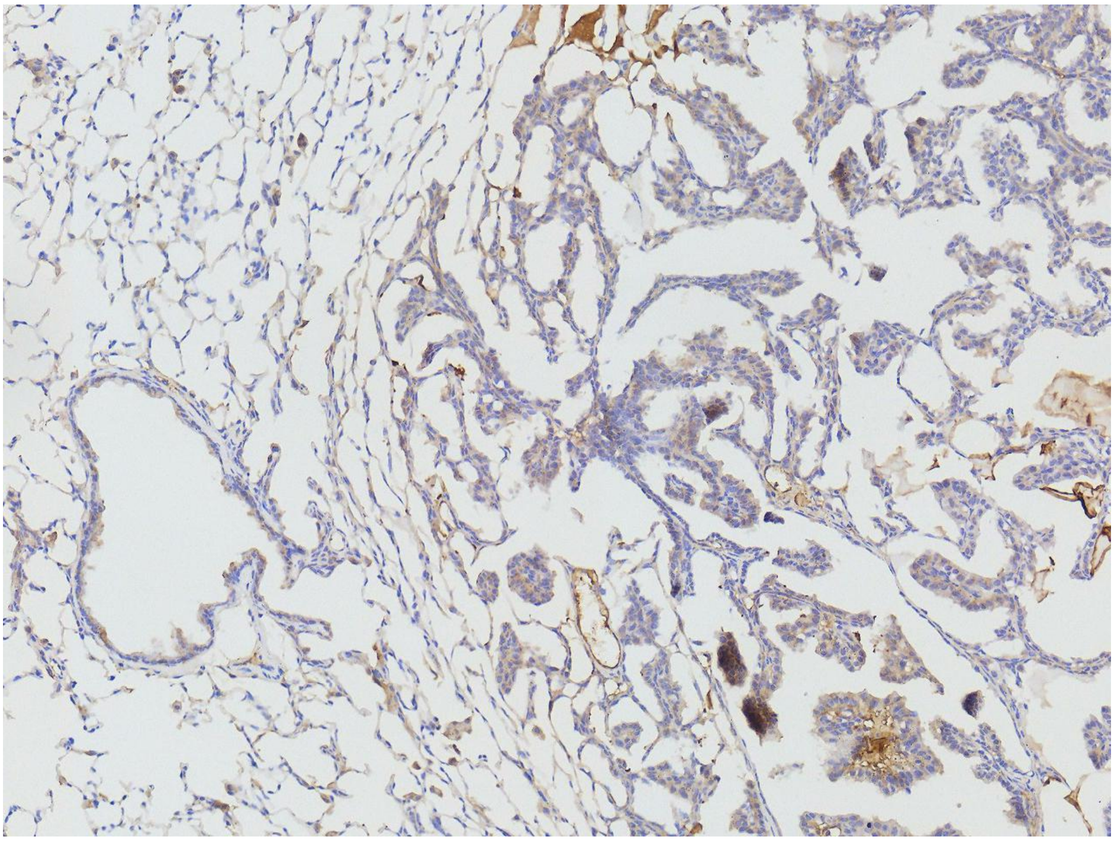

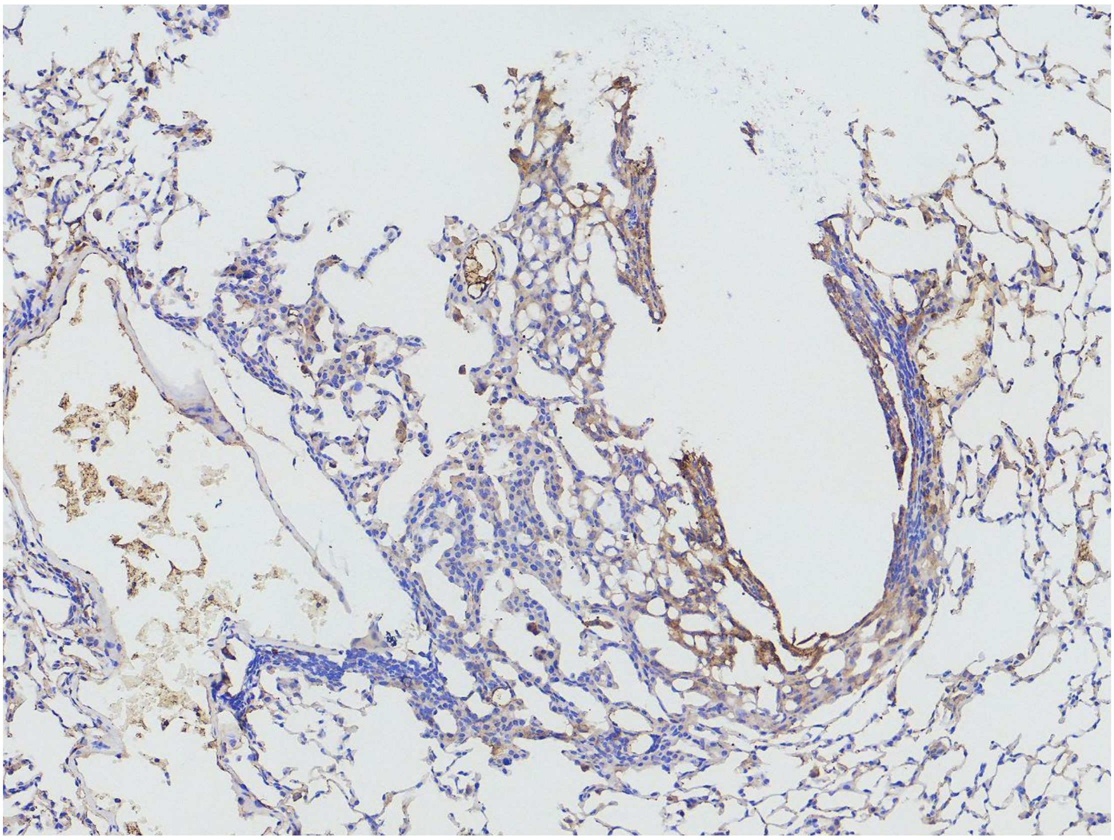

The original IHC images in Figure 6G.

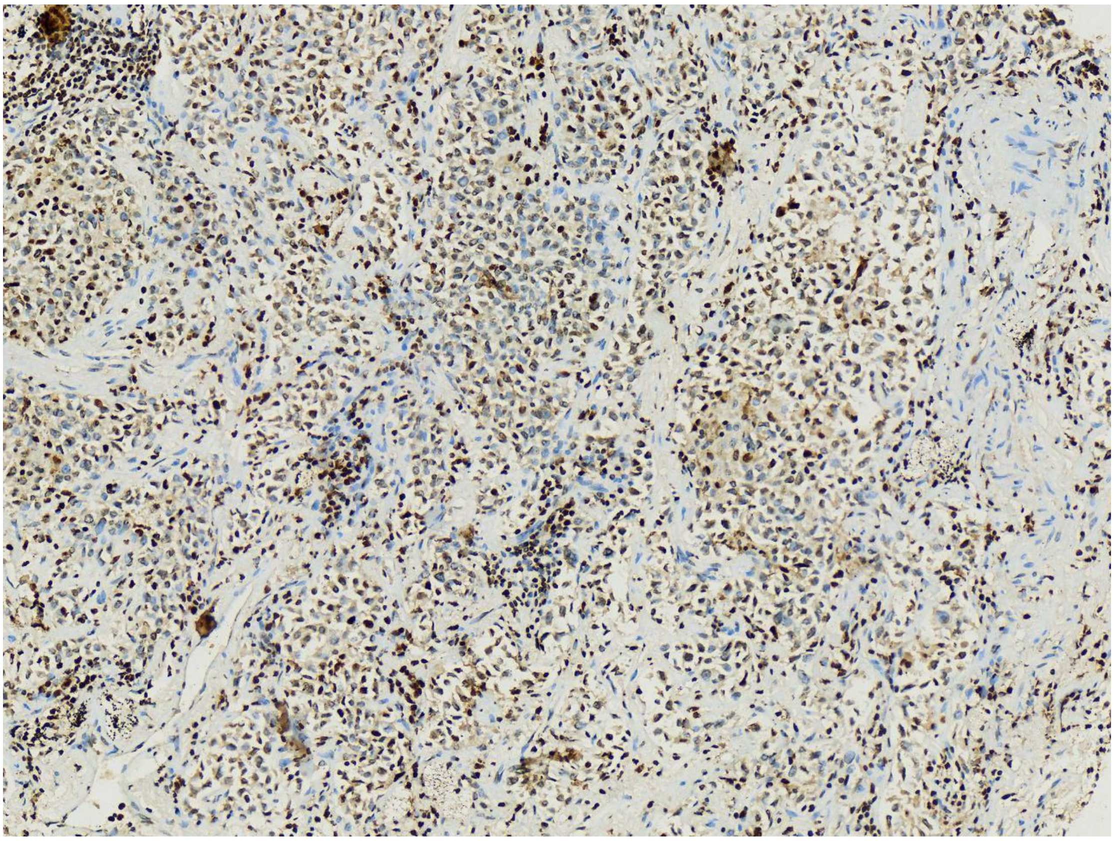

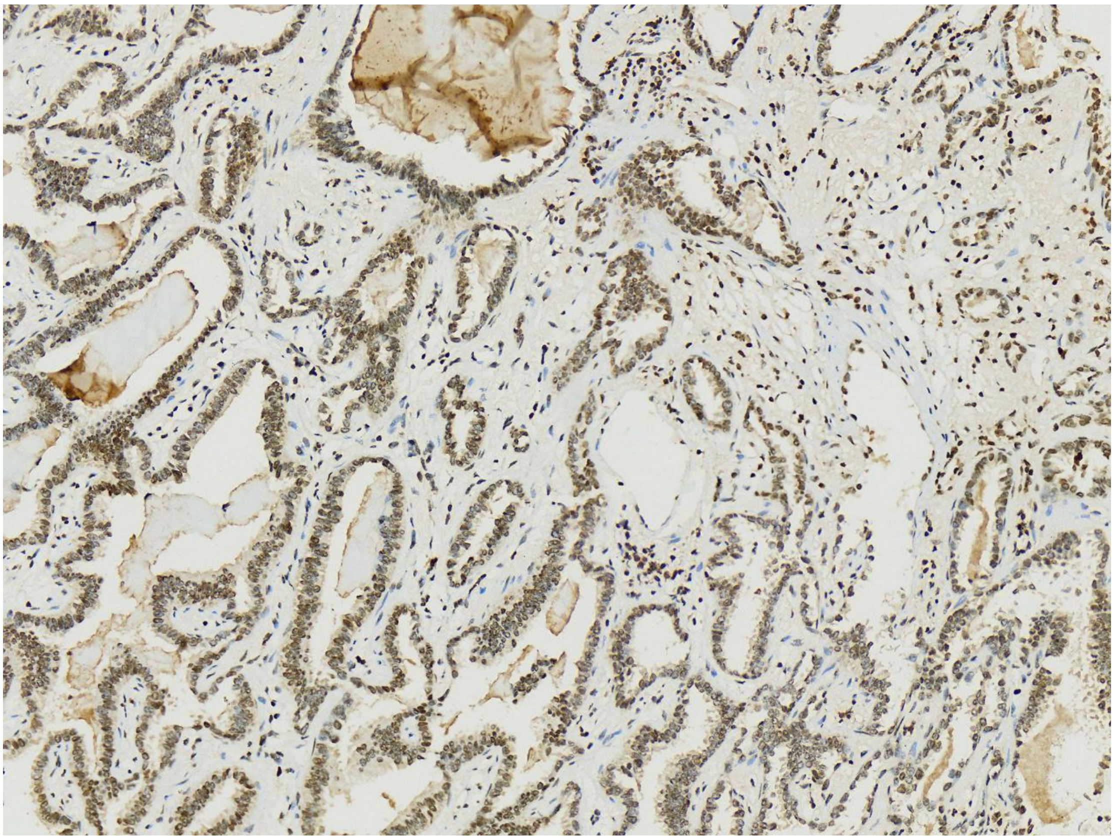

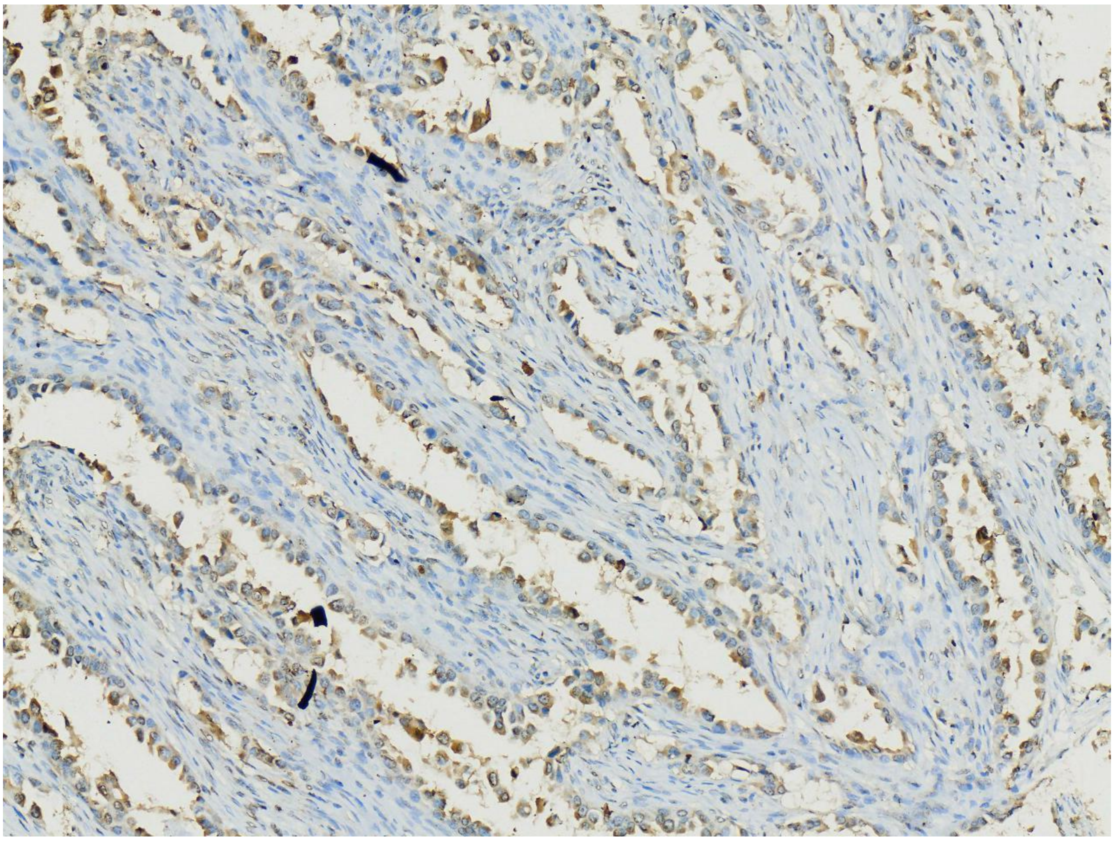

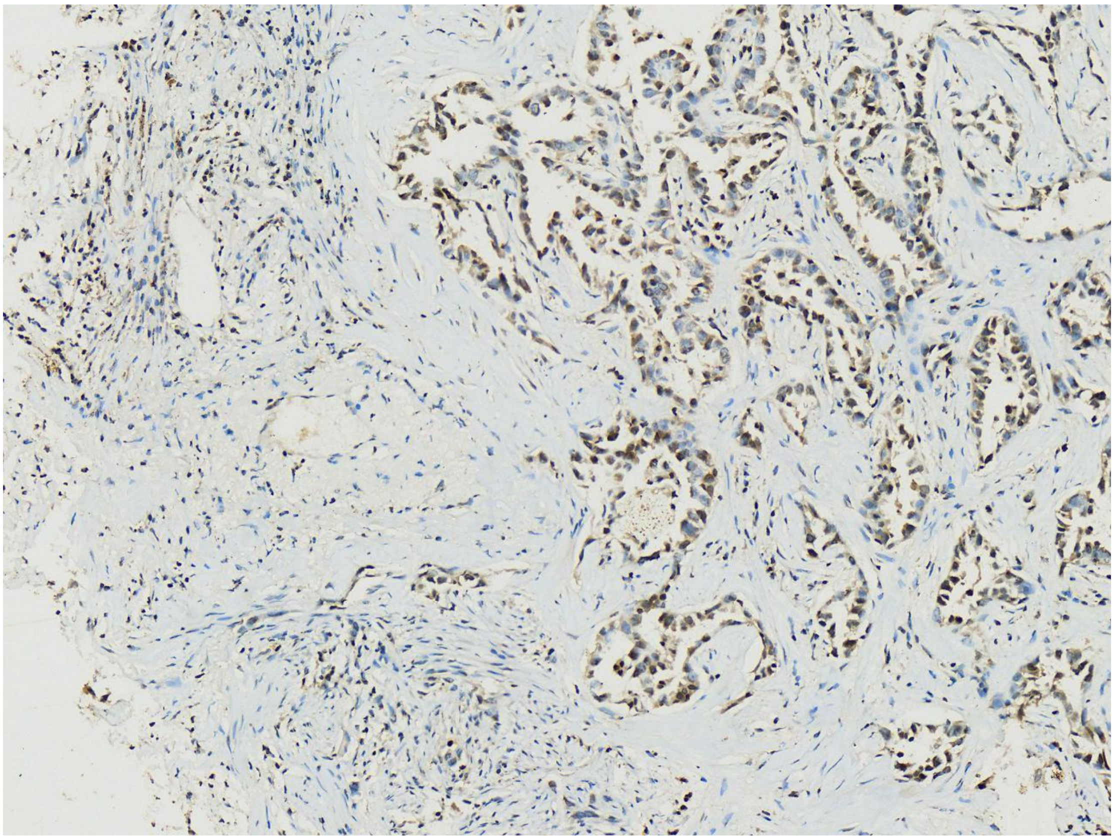

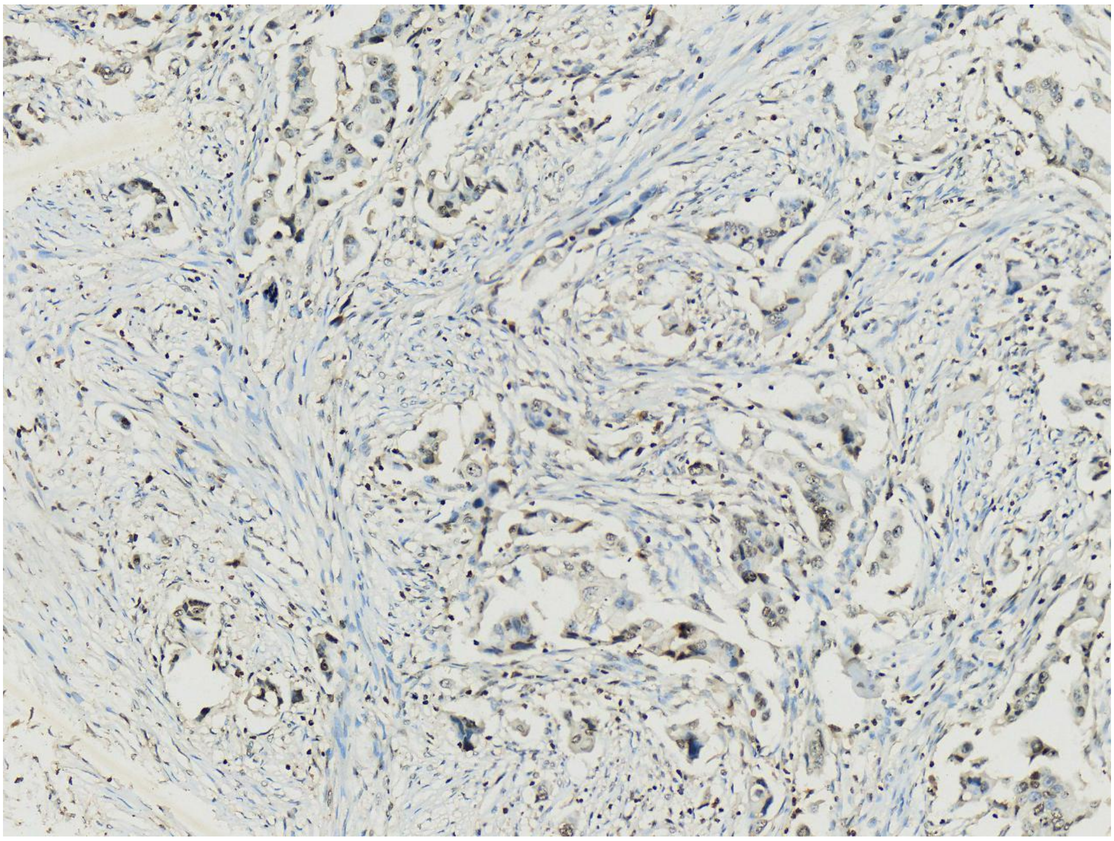

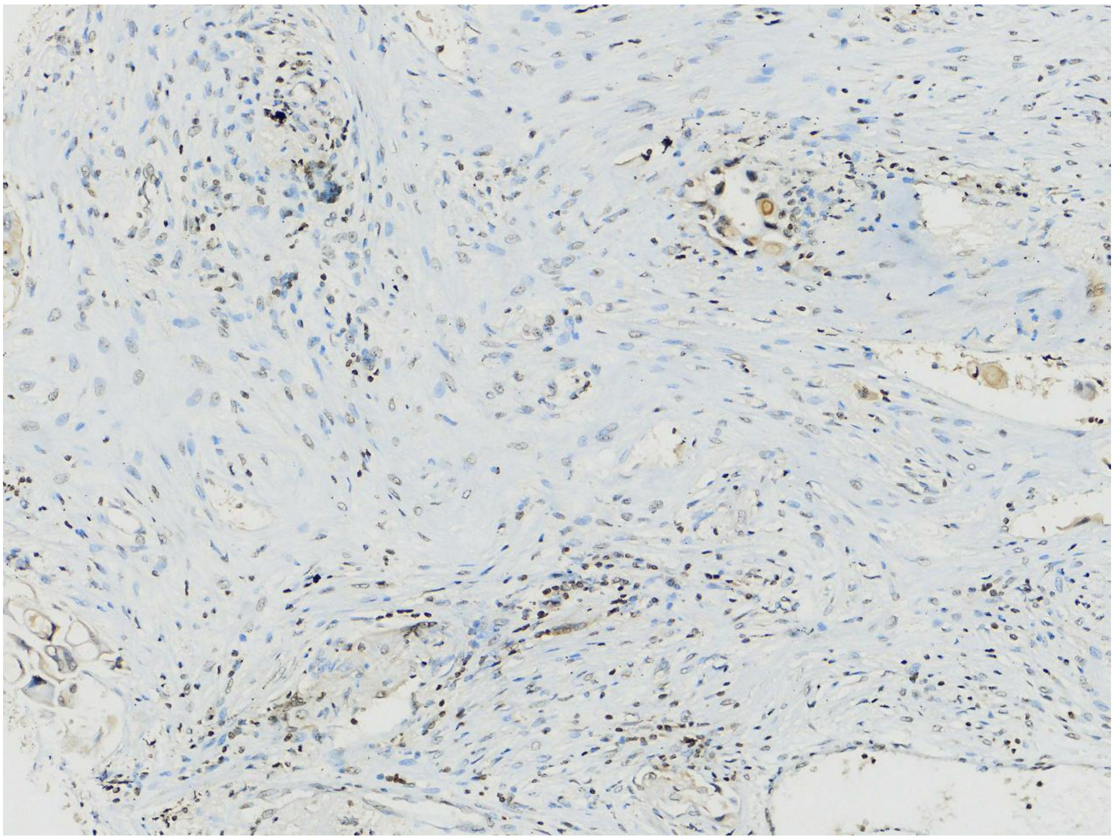

Supplement: Supplementary file 7 — Supplementary Material 7 [file 13578_2025_1348_MOESM7_ESM.pdf]
